# Supplementary material for: Breastfeeding practice, breastfeeding policy and hospitalisations for infectious diseases in early and later childhood: a register-based study in Uppsala County, Sweden
Source: BMJ Open. 2021 May 30;11(5):e046583. doi: 10.1136/bmjopen-2020-046583 (PMC8169467; doi:10.1136/bmjopen-2020-046583)
Supplement: Supplementary data [file bmjopen-2020-046583supp001.pdf]

Appendix A

Table A: Infectious disease categories and associated ICD-10 codes

Category	Group	ICD codes
Enteric infections	Enteric infections	A00, A01, A020, A029, A03, A04, A05, A06, A07,A08
	Enteric symptoms	A09, I880, K528, K529, R11
Upper respiratory tract infections	Upper RTI	J00, J01, J02, J03, J04, J05, J06, J32, J340, J36, J37, J390, J391
	Ear infections	H600, H601, H602, H603, H608, H609, H62, H65, H66, H67, H680, H70, H730, H750, H830, H940
Lower respiratory tract infections	Acute lower RTI	A481, A482, B59, J09, J10, J11, J12, J13, J14, J15, J16, J17, J18, J20, J21, J22
	Chronic lower RTI	J40, J41, J42, J440, J47, J85, J86, J988
	Tuberculosis	A15, A16, A17, A18, A19, N740, N741, J65
Other infections	Sepsis	A391, A392, A393, A394, A395, A398, A399 A40, A41, A483, A021, A327
	HIV/AIDS	B20, B21. B22, B23, B24
	STI	A50, A51, A52, A53, A54, A55, A56, A57, A58, A59, A60, A63, A64, N290
	CNS viral infections	A801, A802, A803, A804, A809, A811, A812, A818, A819, A82, A83, A84, A85, A86, A87, A88, A89
	CNS general infections	G00, G01, G02, G030, G039, G04, G05, G06, G07, G08, G09, G610, A321, A390
	Eye infections	B30, H000, H03, H043, H050, H100, H102, H103, H109, H130, H131, H160, H190, H191, H192, H220, H440, H451
	Oral infections	K02, K044, K046, K050, K052, K053, K113, K122
	Heart and Circulatory infections	B332, I00, I01, I02, I05, I06, I07, I08, I09, I301, I33, I38, I39, I400, I410, I411, I412, I430, I716, I790, I791
	Gastrointestinal tract infections	K230, K231, K25, K26, K27, K28, K293, K294, K295, K35, K36, K37, K61, K630, K632, K650, K678, K908, K930
	Hepatic infections	K750, K770, K830
	Viral Hepatitis	B15, B16, B17, B18, B19

Kidney infections	N00, N05, N10, N136, N151
Urinary tract infections	N300, N341, N351, N37, N390
Reproductive system infections, male	N410, N411, N412, N413, N431, N45, N410, N411, N412, N413, N431, N45, N481, N482, N490, N49, N51
Reproductive system infections, female	N70, N71, N72, N73, N74, N751, N764, N87
Skin infections, typical	A46, L00, L01, L02, L03, L04, L050, L08
Skin infections, other	B86, T009, T633, T634, T793
Breast infections	N61
Osteomyelitis	M462, M463, M464, M465
Joint infections	M00, M01
Connective tissue infections	M021, M023, M03, M600, M630, M631, M632, M650, M651, M680, M710, M711, M896
Neoplasms from infection	C11, C161, C162, C163, C164, C165, C166, C168, C169, C210, C211, C220, C46, C53, D002, D013, D06
Postoperative infections	T802, T814, T826, T827, T835, T836, T845, T846, T847, T857, T874
Adverse effect of ID treatment	R761, R762, T36, T37, T485, T487, T490, T495, T496, T499, T788, T789, T880, T881, T887
Other Bacterial infections	A20, A21, A22, A23, A24, A25, A26, A27, A28, A30, A31, A320, A328, A329, A33, A34, A35, A36, A37, A38, A42, A43, A44, A480, A484, A488, A49, A65, A66, A67, A68, A69, A70, A71, A74, A75, A77, A78, A79, B95, B96
Other Viral infections	A90, A91, A92, A93, A94, A95, A96, A98, A99, B00, B01, B02, B03, B04, B05, B06, B07, A08, B09, B25, B26, B27, B33, B34, B97
Other Mycoses	B35, B36, B37, B38, B39, B40, B41, B42, B43, B44, B45, B46, B47, B48, B49
Other Protozoan infections	B50, B51, B52, B53, B54, B55, B56, B57, B58, B60, B64
Other ID	B65, B66, B67, B68, B69, B70, B71, B72, B73, B74, B75, B76, B77, B78, B79, B80, B81, B82, B83, B85, B86, B87, B88, B89, B94, B99, E033, E321, F024, F071, I88, T64
Perinatal infections	P002, P027, P23, P35, P36, P37, P38, P39

ICD-10, International Classification of Disease, Tenth Revision. RTI, respiratory tract infections. CNS, central nervous system. ID, infectious diseases.
