# Supplementary material for: Breastfeeding practice, breastfeeding policy and hospitalisations for infectious diseases in early and later childhood: a register-based study in Uppsala County, Sweden
Source: BMJ Open. 2021 May 30;11(5):e046583. doi: 10.1136/bmjopen-2020-046583 (PMC8169467; doi:10.1136/bmjopen-2020-046583)
Supplement: Supplementary data [file bmjopen-2020-046583supp002.pdf]

**Appendix B**

**Table B: Regression results for all infections, early childhood (<2 years)**

| | Crude* | (95% CI) | Adjusted† | (95% CI) | 1998-2003‡ | (95% CI) | 2004-2010§ | (95% CI) | BMI¶ | (95% CI) |
|---|---|---|---|---|---|---|---|---|---|---|
| Breastfeeding: | | | | | | | | | | |
| No BF | 2.13 | (1.61–2.81) | 1.89 | (1.45–2.47) | 2.25 | (1.51–3.35) | 1.64 | (1.17–2.32) | 1.81 | (1.35–2.42) |
| EBF <4 MO with BF <6 MO | 1.46 | (1.30–1.64) | 1.41 | (1.25–1.59) | 1.34 | (1.13–1.59) | 1.48 | (1.25–1.74) | 1.40 | (1.23–1.59) |
| EBF <4 MO with BF ≥6 MO | 1.33 | (1.16–1.52) | 1.31 | (1.14–1.50) | 1.26 | (1.02–1.56) | 1.35 | (1.12–1.63) | 1.29 | (1.11–1.49) |
| EBF 4–5 MO with BF <6 MO | 1.19 | (0.95–1.50) | 1.16 | (0.92–1.46) | 1.21 | (0.86–1.71) | 1.11 | (0.84–1.47) | 1.09 | (0.84–1.40) |
| EBF 4–5 MO with BF ≥6 MO | 1.04 | (0.94–1.15) | 1.05 | (0.94–1.16) | 1.06 | (0.92–1.23) | 1.04 | (0.89–1.20) | 1.04 | (0.93–1.16) |
| EBF ≥6 MO | 1 | ref | 1 | ref | 1 | ref | 1 | ref | 1 | ref |
| SGA: | | | | | | | | | | |
| Yes | | | 1.58 | (1.14–2.19) | 1.59 | (0.98–2.57) | 1.55 | (0.99–2.43) | 1.60 | (1.12–2.28) |
| No | | | 1 | ref | 1 | ref | 1 | ref | 1 | ref |
| LGA: | | | | | | | | | | |
| Yes | | | 1.22 | (1.02–1.47) | 1.40 | (1.06–1.84) | 1.08 | (0.86–1.36) | 1.21 | (0.99–1.47) |
| No | | | 1 | ref | 1 | ref | 1 | ref | 1 | ref |
| Congenital malformation: | | | | | | | | | | |
| Yes | | | 2.25 | (1.82–2.79) | 2.18 | (1.64–2.90) | 2.32 | (1.71–3.17) | 2.27 | (1.82–2.84) |
| No | | | 1 | ref | 1 | ref | 1 | ref | 1 | ref |
| Sex: | | | | | | | | | | |
| Male | | | 1 | ref | 1 | ref | 1 | ref | 1 | ref |
| Female | | | 0.89 | (0.82–0.96) | 0.95 | (0.85–1.07) | 0.83 | (0.75–0.93) | 0.88 | (0.81–0.96) |
| Maternal age (years): | | | | | | | | | | |
| ≤19 | | | 1.00 | (0.71–1.42) | 1.03 | (0.65–1.63) | 0 | (0.59–1.63) | 1.08 | (0.75–1.55) |
| 20-24 | | | 1 | ref | 1 | ref | 1 | ref | 1 | ref |
| 25-29 | | | 1.00 | (0.86–1.15) | 1.06 | (0.85–1.32) | 0.96 | (0.79–1.17) | 1.01 | (0.86–1.18) |
| 30-34 | | | 0.95 | (0.82–1.10) | 1.01 | (0.80–1.28) | 0.91 | (0.75–1.11) | 0.95 | (0.81–1.11) |
| ≥35 | | | 0.88 | (0.74–1.05) | 1.01 | (0.77–1.32) | 0.80 | (0.64–1.00) | 0.89 | (0.74–1.07) |
| Parity: | | | | | | | | | | |
| 1 | | | 1 | ref | 1 | ref | 1 | ref | 1 | ref |
| 2 | | | 1.18 | (1.07–1.29) | 1.06 | (0.93–1.22) | 1.29 | (1.14–1.46) | 1.17 | (1.06–1.30) |
| 3 | | | 1.17 | (1.03–1.33) | 1.04 | (0.86–1.25) | 1.30 | (1.10–1.55) | 1.17 | (1.03–1.34) |
| ≥4 | | | 1.42 | (1.18–1.71) | 1.23 | (0.95–1.61) | 1.62 | (1.25–2.10) | 1.47 | (1.21–1.79) |

| | | | | | | | | | | |
|---|---|---|---|---|---|---|---|---|---|---|
| Maternal smoking: | | | | | | | | | | |
| No smoking | | | 1 | ref | 1 | ref | 1 | ref | 1 | ref |
| Smoking | | | 1.04 | (0.89–1.21) | 0.99 | (0.80–1.23) | 1.10 | (0.89–1.37) | 1.04 | (0.89–1.22) |
| Maternal education (years): | | | | | | | | | | |
| ≤9 | | | 1.07 | (0.91–1.25) | 1.09 | (0.87–1.37) | 1.03 | (0.83–1.29) | 1.05 | (0.89–1.24) |
| 10-12 | | | 1.03 | (0.93–1.13) | 1.01 | (0.86–1.17) | 1.04 | (0.91–1.18) | 1.04 | (0.93–1.16) |
| 13-14 | | | 1.01 | (0.88–1.15) | 1.00 | (0.83–1.21) | 1.00 | (0.83–1.21) | 1.02 | (0.88–1.17) |
| ≥15 | | | 1 | ref | 1 | ref | 1 | ref | 1 | ref |
| Maternal country of birth: | | | | | | | | | | |
| Sweden | | | 1 | ref | 1 | ref | 1 | ref | 1 | ref |
| Other Nordic | | | 1.06 | (0.82–1.37) | 1.09 | (0.78–1.54) | 1.03 | (0.70–1.52) | 1.07 | (0.82–1.40) |
| Europe and North America | | | 1.04 | (0.86–1.25) | 1.15 | (0.87–1.51) | 0.96 | (0.74–1.25) | 1.08 | (0.88–1.33) |
| Asia | | | 0.94 | (0.79–1.12) | 0.88 | (0.67–1.15) | 0.98 | (0.79–1.23) | 0.93 | (0.77–1.13) |
| Africa | | | 1.41 | (1.08–1.84) | 1.35 | (0.93–1.94) | 1.45 | (1.01–2.10) | 1.48 | (1.12–1.95) |
| Other | | | 0.92 | (0.58–1.45) | 1.00 | (0.48–2.10) | 0.84 | (0.49–1.45) | 0.84 | (0.54–1.30) |
| Year of birth: | | | | | | | | | | |
| 1998 | 1 | ref | 1 | ref | 1 | ref | | | 1 | ref |
| 1999 | 0.80 | (0.64–0.99) | 0.81 | (0.66–1.01) | 0.81 | (0.66–1.00) | | | 0.79 | (0.63–0.99) |
| 2000 | 0.99 | (0.82–1.21) | 1.02 | (0.84–1.24) | 1.01 | (0.83–1.22) | | | 1.03 | (0.84–1.26) |
| 2001 | 0.89 | (0.74–1.09) | 0.93 | (0.77–1.13) | 0.93 | (0.76–1.12) | | | 0.94 | (0.76–1.16) |
| 2002 | 0.87 | (0.72–1.06) | 0.90 | (0.74–1.09) | 0.89 | (0.73–1.08) | | | 0.92 | (0.75–1.12) |
| 2003 | 0.79 | (0.65–0.96) | 0.82 | (0.68–0.99) | 0.81 | (0.67–0.98) | | | 0.78 | (0.63–0.95) |
| 2004 | 0.84 | (0.70–1.02) | 0.89 | (0.73–1.07) | | | 1 | ref | 0.94 | (0.77–1.15) |
| 2005 | 0.84 | (0.70–1.02) | 0.88 | (0.73–1.06) | | | 0.99 | (0.83–1.20) | 0.90 | (0.74–1.10) |
| 2006 | 0.73 | (0.60–0.89) | 0.77 | (0.64–0.93) | | | 0.87 | (0.73–1.05) | 0.76 | (0.62–0.93) |
| 2007 | 0.69 | (0.57–0.84) | 0.73 | (0.60–0.88) | | | 0.82 | (0.68–0.99) | 0.71 | (0.58–0.87) |
| 2008 | 0.58 | (0.47–0.71) | 0.61 | (0.50–0.75) | | | 0.69 | (0.57–0.84) | 0.61 | (0.49–0.75) |
| 2009 | 0.59 | (0.48–0.73) | 0.62 | (0.51–0.77) | | | 0.70 | (0.57–0.87) | 0.62 | (0.50–0.77) |
| 2010 | 0.55 | (0.44–0.67) | 0.59 | (0.48–0.72) | | | 0.66 | (0.54–0.81) | 0.58 | (0.47–0.72) |
| Pregnancy BMI: | | | | | | | | | | |
| Underweight | | | | | | | | | 1.09 | (0.82–1.44) |
| Normal | | | | | | | | | 1 | ref |
| Overweight | | | | | | | | | 0.98 | (0.89–1.09) |
| Obese | | | | | | | | | 1.12 | (0.98–1.28) |
| Observations (N): | 37,825 | | 37,825 | | 15,553 | | 22,272 | | 33,422 | |

| | | | | | |
|---|---|---|---|---|---|
| Clusters (Maternal ID): | 25,487 | 25,487 | 12,445 | 16,998 | 23,605 |

*Adjusted for year of birth. Analysis included 37,825 children.

†Adjusted for year of birth small for gestational age, large for gestational age, congenital malformation, sex, maternal age, parity, maternal smoking, maternal education level and maternal country of birth. Analysis included 37,825 children.

‡Included children born 1998 to 2003. Adjusted for year of birth small for gestational age, large for gestational age, congenital malformation, sex, maternal age, parity, maternal smoking, maternal education level and maternal country of birth. Analysis excluded children born 2004 to 2010, leaving 15,553 children.

§Included children born 2004 to 2010. Adjusted for year of birth small for gestational age, large for gestational age, congenital malformation, sex, maternal age, parity, maternal smoking, maternal education level and maternal country of birth. Analysis excluded children born 1998 to 2003, leaving 22,272 children.

¶Adjusted for year of birth small for gestational age, large for gestational age, congenital malformation, sex, maternal age, parity, maternal smoking, maternal education level, maternal country of birth and pregnancy BMI. Analysis excluded children with missing data on pregnancy BMI, leaving 33,422 children.

Incidence rate ratios were estimated using negative binomial regression models. Correlation between siblings was accounted for using generalized estimating equations with robust standard errors. CI, confidence intervals. BF, breastfeeding. EBF, exclusive breastfeeding. MO, months. SGA, small for gestation age. LGA, large for gestation age. BMI, body mass index.

**Table C: Regression results for all infections, later childhood (2–4 years)**

| | Crude* | (95% CI) | Adjusted† | (95% CI) | Adjusted 2‡ | (95% CI) | 1998-2003§ | (95% CI) | 2004-2010¶ | (95% CI) | BMI# | (95% CI) |
|---|---|---|---|---|---|---|---|---|---|---|---|---|
| Breastfeeding: | | | | | | | | | | | | |
| No BF | 1.98 | (1.27–3.09) | 1.73 | (1.12–2.66) | 1.52 | (1.00–2.33) | 2.51 | (1.54–4.09) | 0.82 | (0.37–1.82) | 1.54 | (0.98–2.40) |
| EBF <4 MO with BF <6 MO | 1.60 | (1.29–1.98) | 1.52 | (1.23–1.87) | 1.43 | (1.16–1.75) | 1.29 | (0.97–1.71) | 1.54 | (1.13–2.08) | 1.46 | (1.17–1.82) |
| EBF <4 MO with BF ≥6 MO | 1.35 | (1.07–1.71) | 1.31 | (1.03–1.66) | 1.27 | (1.00–1.61) | 1.39 | (1.01–1.91) | 1.22 | (0.86–1.73) | 1.32 | (1.03–1.69) |
| EBF 4–5 MO with BF <6 MO | 0.98 | (0.69–1.38) | 0.97 | (0.69–1.38) | 0.97 | (0.68–1.37) | 0.90 | (0.59–1.38) | 1.08 | (0.61–1.89) | 1.01 | (0.70–1.47) |
| EBF 4–5 MO with BF ≥6 MO | 1.05 | (0.88–1.24) | 1.05 | (0.88–1.24) | 1.05 | (0.88–1.24) | 1.07 | (0.86–1.34) | 1.04 | (0.81–1.35) | 1.04 | (0.86–1.24) |
| EBF ≥6 MO | 1 | ref | 1 | ref | 1 | ref | 1 | ref | 1 | ref | 1 | ref |
| SGA: | | | | | | | | | | | | |
| Yes | | | 2.23 | (1.43–3.46) | 2.12 | (1.37–3.29) | 1.68 | (0.88–3.23) | 2.49 | (1.41–4.40) | 2.26 | (1.45–3.53) |
| No | | | 1 | ref | 1 | ref | 1 | ref | 1 | ref | 1 | ref |
| LGA: | | | | | | | | | | | | |
| Yes | | | 1.21 | (0.91–1.60) | 1.17 | (0.89–1.54) | 1.03 | (0.68–1.53) | 1.33 | (0.92–1.93) | 1.08 | (0.80–1.45) |
| No | | | 1 | ref | 1 | ref | 1 | ref | 1 | ref | 1 | ref |
| Congenital malformation: | | | | | | | | | | | | |
| Yes | | | 3.44 | (2.36–5.03) | 2.89 | (2.03–4.11) | 2.37 | (1.66–3.39) | 3.42 | (1.97–5.95) | 2.96 | (2.04–4.29) |
| No | | | 1 | ref | 1 | ref | 1 | ref | 1 | ref | 1 | ref |
| Sex | | | | | | | | | | | | |
| Male | | | 1 | ref | 1 | ref | 1 | ref | 1 | ref | 1 | ref |
| Female | | | 0.90 | (0.78–1.04) | 0.91 | (0.79–1.05) | 0.94 | (0.78–1.12) | 0.87 | (0.71–1.07) | 0.92 | (0.79–1.07) |
| Maternal age (years): | | | | | | | | | | | | |
| ≤19 | | | 0.64 | (0.33–1.24) | 0.65 | (0.33–1.26) | 0.67 | (0.27–1.53) | 0.67 | (0.24–1.88) | 0.67 | (0.33–1.35) |
| 20–24 | | | 1 | ref | 1 | ref | 1 | ref | 1 | ref | 1 | ref |
| 25–29 | | | 0.98 | (0.78–1.25) | 0.98 | (0.77–1.23) | 0.96 | (0.71–1.28) | 0.99 | (0.70–1.39) | 1.00 | (0.78–1.27) |
| 30–34 | | | 0.82 | (0.65–1.04) | 0.82 | (0.65–1.04) | 1.02 | (0.75–1.39) | 0.66 | (0.47–0.93) | 0.81 | (0.63–1.04) |
| ≥35 | | | 0.96 | (0.73–1.25) | 0.98 | (0.75–1.27) | 1.22 | (0.86–1.74) | 0.81 | (0.56–1.18) | 0.97 | (0.73–1.29) |
| Parity: | | | | | | | | | | | | |
| 1 | | | 1 | ref | 1 | ref | 1 | ref | 1 | ref | 1 | ref |
| 2 | | | 0.98 | (0.84–1.14) | 0.96 | (0.83–1.12) | 0.90 | (0.73–1.12) | 1.04 | (0.84–1.30) | 0.98 | (0.83–1.15) |
| 3 | | | 0.99 | (0.80–1.23) | 0.97 | (0.78–1.20) | 0.85 | (0.63–1.15) | 1.11 | (0.82–1.50) | 0.97 | (0.78–1.23) |
| ≥4 | | | 0.99 | (0.67–1.48) | 0.94 | (0.64–1.36) | 0.60 | (0.39–0.92) | 1.33 | (0.81–2.18) | 0.96 | (0.65–1.43) |
| Maternal smoking: | | | | | | | | | | | | |
| No smoking | | | 1 | ref | 1 | ref | 1 | ref | 1 | ref | 1 | ref |

| | | | | | | | | | | | | |
|---|---|---|---|---|---|---|---|---|---|---|---|---|
| Smoking | | | 1.04 | (0.80–1.37) | 1.04 | (0.80–1.34) | 0.92 | (0.68–1.25) | 1.16 | (0.78–1.73) | 0.96 | (0.73–1.27) |
| Maternal education (years): | | | | | | | | | | | | |
| ≤9 | | | 1.15 | (0.87–1.53) | 1.13 | (0.86–1.49) | 1.24 | (0.89–1.73) | 0.99 | (0.65–1.53) | 1.19 | (0.89–1.57) |
| 10–12 | | | 0.90 | (0.76–1.07) | 0.90 | (0.76–1.07) | 0.98 | (0.77–1.26) | 0.81 | (0.64–1.03) | 0.91 | (0.75–1.09) |
| 13–14 | | | 1.11 | (0.91–1.37) | 1.12 | (0.92–1.37) | 1.10 | (0.82–1.46) | 1.13 | (0.84–1.51) | 1.10 | (0.89–1.37) |
| ≥15 | | | 1 | ref | 1 | ref | 1 | ref | 1 | ref | 1 | ref |
| Maternal country of birth: | | | | | | | | | | | | |
| Sweden | | | 1 | ref | 1 | ref | 1 | ref | 1 | ref | 1 | ref |
| Other Nordic | | | 0.91 | (0.47–1.78) | 0.89 | (0.46–1.72) | 0.66 | (0.33–1.31) | 1.20 | (0.43–3.37) | 0.90 | (0.44–1.84) |
| Europe and North America | | | 0.99 | (0.72–1.37) | 0.98 | (0.71–1.35) | 1.09 | (0.68–1.72) | 0.90 | (0.58–1.38) | 0.86 | (0.60–1.24) |
| Asia | | | 1.04 | (0.77–1.40) | 1.05 | (0.78–1.40) | 0.88 | (0.60–1.27) | 1.17 | (0.77–1.76) | 1.12 | (0.82–1.51) |
| Africa | | | 1.23 | (0.70–2.18) | 1.14 | (0.66–1.97) | 1.19 | (0.67–2.12) | 1.12 | (0.48–2.61) | 1.14 | (0.64–2.02) |
| Other | | | 1.05 | (0.54–2.05) | 1.03 | (0.56–1.89) | 1.33 | (0.58–3.04) | 0.76 | (0.32–1.80) | 0.90 | (0.49–1.66) |
| Year of birth: | | | | | | | | | | | | |
| 1998 | 1 | ref | 1 | ref | 1 | ref | | | 1 | ref | 1 | ref |
| 1999 | 1.01 | (0.74–1.38) | 1.05 | (0.74–1.38) | 1.10 | (0.80–1.50) | 1.07 | (0.79–1.46) | | | 1.18 | (0.85–1.62) |
| 2000 | 0.99 | (0.72–1.36) | 1.04 | (0.72–1.36) | 1.03 | (0.76–1.41) | 1.02 | (0.75–1.39) | | | 1.00 | (0.72–1.39) |
| 2001 | 0.93 | (0.67–1.29) | 0.99 | (0.67–1.29) | 0.99 | (0.72–1.36) | 0.97 | (0.71–1.32) | | | 0.91 | (0.64–1.30) |
| 2002 | 0.97 | (0.71–1.31) | 1.01 | (0.71–1.31) | 1.03 | (0.76–1.39) | 1.00 | (0.74–1.35) | | | 1.05 | (0.76–1.44) |
| 2003 | 0.94 | (0.69–1.29) | 0.98 | (0.69–1.29) | 1.01 | (0.74–1.37) | 0.97 | (0.72–1.33) | | | 1.06 | (0.76–1.48) |
| 2004 | 0.72 | (0.52–1.00) | 0.77 | (0.52–1.00) | 0.78 | (0.56–1.07) | 1 | ref | | | 0.88 | (0.63–1.23) |
| 2005 | 0.79 | (0.55–1.12) | 0.83 | (0.55–1.12) | 0.83 | (0.59–1.17) | | | 1.07 | (0.76–1.50) | 0.77 | (0.54–1.11) |
| 2006 | 0.56 | (0.40–0.80) | 0.60 | (0.40–0.80) | 0.62 | (0.44–0.87) | | | 0.80 | (0.57–1.12) | 0.62 | (0.43–0.90) |
| 2007 | 0.70 | (0.50–1.00) | 0.74 | (0.50–1.00) | 0.78 | (0.55–1.10) | | | 1.01 | (0.72–1.44) | 0.79 | (0.55–1.14) |
| 2008 | 0.66 | (0.46–0.95) | 0.71 | (0.46–0.95) | 0.76 | (0.53–1.09) | | | 1.00 | (0.70–1.43) | 0.79 | (0.54–1.15) |
| 2009 | 0.68 | (0.46–1.02) | 0.72 | (0.46–1.02) | 0.76 | (0.52–1.13) | | | 0.98 | (0.68–1.42) | 0.77 | (0.52–1.16) |
| 2010 | 0.53 | (0.38–0.74) | 0.58 | (0.38–0.74) | 0.63 | (0.45–0.87) | | | 0.83 | (0.59–1.15) | 0.65 | (0.46–0.91) |
| Previous admissions: | | | | | | | | | | | | |
| Yes | | | | | 3.39 | (2.78–4.12) | 2.86 | (2.24–3.65) | 3.94 | (2.94–5.28) | 3.46 | (2.81–4.26) |
| No | | | | | 1 | ref | 1 | ref | 1 | ref | 1 | ref |
| Pregnancy BMI: | | | | | | | | | | | | |
| Underweight | | | | | | | | | | | 0.74 | (0.45–1.22) |
| Normal | | | | | | | | | | | 1 | ref |
| Overweight | | | | | | | | | | | 1.01 | (0.84–1.20) |
| Obese | | | | | | | | | | | 1.03 | (0.83–1.29) |

| | | | | | | |
|---|---|---|---|---|---|---|
| Observations (N): | 37,621 | 37,621 | 37,621 | 15,466 | 22,155 | 33,236 |
| Clusters (Maternal ID): | 25,344 | 25,344 | 25,344 | 12,377 | 16,910 | 23,471 |

*Adjusted for year of birth. Analysis included 37,621 children.

†Adjusted for year of birth small for gestational age, large for gestational age, congenital malformation, sex, maternal age, parity, maternal smoking, maternal education level and maternal country of birth. Analysis included 37,621 children.

‡Adjusted for year of birth small for gestational age, large for gestational age, congenital malformation, sex, maternal age, parity, maternal smoking, maternal education level, maternal country of birth and previous admissions. Analysis included 37,621 children.

§Included children born 1998 to 2003. Adjusted for year of birth small for gestational age, large for gestational age, congenital malformation, sex, maternal age, parity, maternal smoking, maternal education level, maternal country of birth and previous admissions. Analysis excluded children born 2004 to 2010, leaving 15,466 children.

¶Included children born 2004 to 2010. Adjusted for year of birth small for gestational age, large for gestational age, congenital malformation, sex, maternal age, parity, maternal smoking, maternal education level, maternal country of birth and previous admissions. Analysis excluded children born 1998 to 2003, leaving 22,155 children.

#Adjusted for year of birth small for gestational age, large for gestational age, congenital malformation, sex, maternal age, parity, maternal smoking, maternal education level, maternal country of birth, previous admissions and pregnancy BMI. Analysis excluded children with missing data on pregnancy BMI, leaving 33,236 children.

Incidence rate ratios were estimated using negative binomial regression models. Correlation between siblings was accounted for using generalized estimating equations with robust standard errors. BF, breastfeeding. EBF exclusive breastfeeding. MO, months. SGA, small for gestation age. LGA, large for gestation age. BMI, body mass index.
