# Supplementary material for: Breastfeeding practice, breastfeeding policy and hospitalisations for infectious diseases in early and later childhood: a register-based study in Uppsala County, Sweden
Source: BMJ Open. 2021 May 30;11(5):e046583. doi: 10.1136/bmjopen-2020-046583 (PMC8169467; doi:10.1136/bmjopen-2020-046583)
Supplement: Supplementary data [file bmjopen-2020-046583supp003.pdf]

Appendix C

Table D: Regression results for respiratory infections, early childhood (<2 years)

	Crude*(95% CI)		Adjusted†(95% CI)		1998-2003‡(95% CI)		2004-2010§(95% CI)		BMI¶(95% CI)	
Breastfeeding :										
No BF	1.93	(1.30–2.87)	1.70	(1.15–2.52)	2.18	(1.21–3.93)	1.37	(0.82–2.29)	1.69	(1.11–2.57)
EBF <4 MO with BF <6 MO	1.43	(1.20–1.69)	1.39	(1.17–1.65)	1.38	(1.07–1.78)	1.40	(1.10–1.79)	1.32	(1.10–1.60)
EBF <4 MO with BF ≥6 MO	1.28	(1.04–1.58)	1.29	(1.05–1.59)	1.29	(0.93–1.78)	1.28	(0.98–1.69)	1.21	(0.97–1.51)
EBF 4–5 MO with BF <6 MO	1.02	(0.72–1.44)	0.99	(0.70–1.40)	1.04	(0.62–1.75)	0.95	(0.61–1.49)	0.88	(0.59–1.30)
EBF 4–5 MO with BF ≥6 MO	1.08	(0.93–1.26)	1.09	(0.93–1.26)	1.09	(0.88–1.36)	1.08	(0.88–1.34)	1.04	(0.89–1.22)
EBF ≥6 MO	1	ref	1	ref	1	ref	1	ref	1	ref
SGA:										
Yes			1.85	(1.15–2.97)	1.78	(0.99–3.21)	1.89	(0.94–3.81)	1.92	(1.16–3.20)
No			1	ref	1	ref	1	ref	1	ref
LGA:										
Yes			1.29	(1.02–1.64)	1.50	(1.06–2.10)	1.14	(0.82–1.58)	1.27	(0.98–1.64)
No			1	ref	1	ref	1	ref	1	ref
Congenital malformation:										
Yes			2.35	(1.81–3.05)	2.63	(1.89–3.65)	1.97	(1.29–3.01)	2.36	(1.80–3.11)
No			1	ref	1	ref	1	ref	1	ref
Sex										
Male			1	ref	1	ref	1	ref	1	ref
Female			0.70	(0.62–0.79)	0.78	(0.65–0.93)	0.64	(0.55–0.75)	0.69	(0.61–0.78)
Maternal age (years):										
≤19			0.99	(0.55–1.79)	0.70	(0.28–1.76)	0	(0.61–2.80)	1.10	(0.60–2.04)
20-24			1	ref	1	ref	1	ref	1	ref
25-29			1.05	(0.85–1.30)	1.10	(0.80–1.53)	1.02	(0.77–1.36)	1.06	(0.84–1.34)
30-34			1.05	(0.84–1.31)	1.03	(0.73–1.45)	1.08	(0.81–1.43)	1.06	(0.84–1.35)
≥35			0.97	(0.76–1.25)	1.11	(0.75–1.65)	0.89	(0.64–1.23)	1.00	(0.76–1.31)
Parity										
1			1	ref	1	ref	1	ref	1	ref
2			1.47	(1.28–1.68)	1.31	(1.06–1.61)	1.61	(1.34–1.93)	1.44	(1.24–1.67)
3			1.37	(1.13–1.66)	1.11	(0.81–1.51)	1.63	(1.28–2.07)	1.36	(1.11–1.66)
≥4			1.63	(1.25–2.13)	1.51	(1.01–2.24)	1.75	(1.22–2.50)	1.64	(1.24–2.17)

BMJ Publishing Group Limited (BMJ) disclaims all liability and responsibility arising from any reliance placed on this supplemental material which has been supplied by the author(s)

Maternal smoking:										
No smoking			1	ref	1	ref	1	ref	1	ref
Smoking			1.17	(0.93–1.46)	1.18	(0.86–1.64)	1.14	(0.83–1.55)	1.15	(0.90–1.47)
Maternal education (years):										
≤9			1.00	(0.79–1.26)	0.99	(0.69–1.41)	1.01	(0.74–1.38)	0.99	(0.77–1.27)
10-12			0.98	(0.85–1.13)	0.98	(0.78–1.22)	0.99	(0.83–1.19)	0.99	(0.85–1.15)
13-14			0.93	(0.77–1.13)	0.93	(0.70–1.23)	0.94	(0.72–1.23)	0.94	(0.77–1.15)
≥15			1	ref	1	ref	1	ref	1	ref
Maternal country of birth:										
Sweden			1	ref	1	ref	1	ref	1	ref
Other Nordic			1.03	(0.72–1.48)	1.04	(0.62–1.74)	1.02	(0.61–1.71)	1.05	(0.72–1.52)
Europe and North America			0.68	(0.49–0.95)	0.72	(0.45–1.15)	0.67	(0.42–1.06)	0.75	(0.53–1.06)
Asia			0.81	(0.62–1.06)	0.73	(0.48–1.12)	0.86	(0.61–1.21)	0.84	(0.63–1.12)
Africa			1.17	(0.78–1.74)	0.87	(0.46–1.66)	1.36	(0.82–2.26)	1.25	(0.82–1.90)
Other			0.85	(0.43–1.66)	0.84	(0.24–2.93)	0.85	(0.41–1.77)	0.65	(0.33–1.28)
Year of birth:										
1998	1	ref	1	ref	1	ref			1	ref
1999	0.82	(0.60–1.13)	0.84	(0.62–1.16)	0.84	(0.61–1.15)			0.84	(0.60–1.17)
2000	0.85	(0.62–1.16)	0.88	(0.65–1.20)	0.87	(0.64–1.18)			0.95	(0.69–1.31)
2001	0.94	(0.72–1.24)	0.99	(0.75–1.30)	0.98	(0.75–1.30)			0.97	(0.72–1.30)
2002	0.87	(0.66–1.15)	0.91	(0.69–1.20)	0.90	(0.68–1.19)			0.90	(0.67–1.22)
2003	0.73	(0.55–0.97)	0.77	(0.58–1.02)	0.76	(0.57–1.01)			0.74	(0.54–1.00)
2004	0.71	(0.54–0.93)	0.75	(0.57–0.98)			1	ref	0.81	(0.61–1.08)
2005	0.91	(0.69–1.19)	0.94	(0.72–1.23)			1.26	(0.97–1.66)	1.01	(0.76–1.33)
2006	0.74	(0.56–0.97)	0.78	(0.59–1.03)			1.04	(0.79–1.38)	0.74	(0.55–1.00)
2007	0.85	(0.65–1.11)	0.90	(0.69–1.17)			1.20	(0.92–1.57)	0.89	(0.67–1.18)
2008	0.58	(0.44–0.77)	0.62	(0.47–0.83)			0.83	(0.63–1.11)	0.60	(0.44–0.81)
2009	0.71	(0.54–0.95)	0.76	(0.57–1.01)			1.02	(0.77–1.36)	0.77	(0.57–1.03)
2010	0.64	(0.48–0.85)	0.70	(0.53–0.92)			0.93	(0.70–1.24)	0.67	(0.50–0.91)
Pregancy BMI:										
Underweight									1.02	(0.67–1.56)
Normal									1	ref
Overweight									0.94	(0.81–1.09)
Obese									1.16	(0.96–1.41)
Observations (N):	37,825		37,825		15,553		22,272		33,422	

Clusters (Maternal ID):	25,487	25,487	12,445	16,998	23,605

*Adjusted for year of birth. Analysis included 37,825 children.

†Adjusted for year of birth small for gestational age, large for gestational age, congenital malformation, sex, maternal age, parity, maternal smoking, maternal education level and maternal country of birth. Analysis included 37,825 children.

‡Included children born 1998 to 2003. Adjusted for year of birth small for gestational age, large for gestational age, congenital malformation, sex, maternal age, parity, maternal smoking, maternal education level and maternal country of birth. Analysis excluded children born 2004 to 2010, leaving 15,553 children.

§Included children born 2004 to 2010. Adjusted for year of birth small for gestational age, large for gestational age, congenital malformation, sex, maternal age, parity, maternal smoking, maternal education level and maternal country of birth. Analysis excluded children born 1998 to 2003, leaving 22,272 children.

¶Adjusted for year of birth small for gestational age, large for gestational age, congenital malformation, sex, maternal age, parity, maternal smoking, maternal education level, maternal country of birth and pregnancy BMI. Analysis excluded children with missing data on pregnancy BMI, leaving 33,422 children.

Incidence rate ratios were estimated using negative binomial regression models. Correlation between siblings was accounted for using generalized estimating equations with robust standard errors. CI, confidence intervals. BF, breastfeeding. EBF, exclusive breastfeeding. MO, months. SGA, small for gestation age. LGA, large for gestation age. BMI, body mass index.

Table E: Regression results for respiratory infections, later childhood (2–4 years)

	Crude* (95% CI)		Adjusted† (95% CI)		Adjusted 2‡ (95% CI)		1998-2003§ (95% CI)		2004-2010¶ (95% CI)		BMI# (95% CI)	
Breastfeeding:												
No BF	3.03	(1.78–5.13)	2.70	(1.62–4.50)	2.53	(1.51–4.24)	4.60	(2.49–8.48)	1.06	(0.41–2.76)	2.60	(1.52–4.45)
EBF <4 MO with BF <6 MO	1.56	(1.15–2.11)	1.55	(1.13–2.12)	1.47	(1.08–2.01)	1.58	(1.04–2.40)	1.37	(0.87–2.15)	1.50	(1.07–2.10)
EBF <4 MO with BF ≥6 MO	1.35	(0.98–1.87)	1.32	(0.95–1.83)	1.28	(0.92–1.77)	1.44	(0.89–2.33)	1.18	(0.77–1.83)	1.35	(0.96–1.90)
EBF 4–5 MO with BF <6 MO	0.99	(0.59–1.67)	1.03	(0.61–1.73)	1.03	(0.61–1.73)	0.86	(0.43–1.73)	1.24	(0.59–2.61)	1.11	(0.65–1.91)
EBF 4–5 MO with BF ≥6 MO	1.07	(0.83–1.37)	1.08	(0.84–1.38)	1.07	(0.84–1.38)	1.10	(0.78–1.56)	1.04	(0.73–1.48)	1.08	(0.83–1.40)
EBF ≥6 MO	1	ref	1	ref	1	ref	1	ref	1	ref	1	ref
SGA												
Yes			2.26	(1.26–4.04)	2.26	(1.25–4.07)	1.45	(0.57–3.68)	3.10	(1.50–6.43)	2.44	(1.32–4.50)
No			1	ref	1	ref	1	ref	1	ref	1	ref
LGA												
Yes			1.17	(0.79–1.74)	1.16	(0.78–1.72)	1.05	(0.57–1.92)	1.24	(0.75–2.05)	1.15	(0.76–1.75)
No			1	ref	1	ref	1	ref	1	ref	1	ref
Congenital malformation:												
Yes			3.75	(2.59–5.43)	3.22	(2.24–4.62)	2.82	(1.71–4.66)	3.44	(2.07–5.73)	3.11	(2.11–4.58)
No			1	ref	1	ref	1	ref	1	ref	1	ref
Sex												
Male			1	ref	1	ref	1	ref	1	ref	1	ref
Female			0.79	(0.65–0.95)	0.82	(0.67–0.99)	0.91	(0.69–1.19)	0.73	(0.56–0.95)	0.84	(0.68–1.02)
Maternal age (years):												
≤19			0.58	(0.21–1.61)	0.61	(0.22–1.69)	0.72	(0.13–2.29)	0.70	(0.16–3.06)	0.72	(0.25–2.04)
20-24			1	ref	1	ref	1	ref	1	ref	1	ref
25-29			1.19	(0.85–1.66)	1.19	(0.85–1.66)	1.17	(0.72–1.88)	1.20	(0.75–1.91)	1.24	(0.87–1.78)
30-34			1.06	(0.76–1.48)	1.06	(0.76–1.48)	1.51	(0.94–2.45)	0.75	(0.47–1.19)	1.14	(0.79–1.63)
≥35			1.39	(0.95–2.03)	1.39	(0.95–2.03)	1.88	(1.10–3.22)	1.05	(0.62–1.77)	1.46	(0.97–2.19)
Parity:												
1			1	ref	1	ref	1	ref	1	ref	1	ref
2			1.04	(0.83–1.29)	1.01	(0.81–1.25)	0.85	(0.62–1.16)	1.22	(0.91–1.64)	1.01	(0.80–1.27)
3			0.88	(0.63–1.23)	0.86	(0.62–1.19)	0.56	(0.35–0.92)	1.25	(0.81–1.93)	0.83	(0.58–1.19)
≥4			0.92	(0.60–1.41)	0.88	(0.58–1.36)	0.62	(0.35–1.12)	1.24	(0.67–2.30)	0.94	(0.61–1.47)
Maternal smoking:												

No smoking			1	ref	1	ref	1	ref	1	ref	1	ref
Smoking			0.91	(0.64–1.31)	0.89	(0.63–1.28)	0.93	(0.61–1.44)	0.84	(0.45–1.54)	0.78	(0.52–1.15)
Maternal education (years):												
≤9			1.12	(0.77–1.63)	1.11	(0.76–1.61)	1.36	(0.85–2.17)	0.90	(0.49–1.66)	1.18	(0.80–1.73)
10-12			0.96	(0.76–1.21)	0.96	(0.76–1.22)	1.15	(0.82–1.64)	0.82	(0.60–1.13)	1.02	(0.79–1.31)
13-14			1.13	(0.84–1.51)	1.14	(0.86–1.52)	1.05	(0.69–1.59)	1.28	(0.87–1.90)	1.20	(0.88–1.62)
≥15			1	ref	1	ref	1	ref	1	ref	1	ref
Maternal country of birth:												
Sweden			1	ref	1	ref	1	ref	1	ref	1	ref
Other Nordic			0.82	(0.44–1.52)	0.80	(0.43–1.48)	0.80	(0.33–1.91)	0.77	(0.32–1.86)	0.78	(0.40–1.50)
Europe and North America			0.58	(0.33–1.03)	0.60	(0.34–1.07)	0.74	(0.32–1.75)	0.50	(0.24–1.07)	0.48	(0.25–0.89)
Asia			1.05	(0.67–1.62)	1.08	(0.70–1.68)	1.05	(0.62–1.78)	1.04	(0.54–1.99)	1.15	(0.75–1.78)
Africa			1.15	(0.46–2.85)	1.10	(0.44–2.77)	1.21	(0.53–2.79)	1.00	(0.23–4.32)	1.10	(0.41–2.98)
Other			1.48	(0.58–3.80)	1.38	(0.59–3.20)	2.25	(0.89–5.65)	0.61	(0.15–2.41)	1.00	(0.42–2.40)
Year of birth:												
1998	1	ref	1	ref	1	ref			1	ref	1	ref
1999	1.02	(0.63–1.64)	1.07	(0.63–1.64)	1.11	(0.69–1.78)	1.10	(0.68–1.76)			1.18	(0.72–1.94)
2000	1.18	(0.74–1.88)	1.25	(0.74–1.88)	1.27	(0.80–2.02)	1.25	(0.79–1.97)			1.32	(0.80–2.15)
2001	1.02	(0.63–1.66)	1.08	(0.63–1.66)	1.07	(0.67–1.71)	1.02	(0.64–1.62)			1.02	(0.61–1.71)
2002	0.85	(0.53–1.36)	0.88	(0.53–1.36)	0.89	(0.56–1.43)	0.87	(0.54–1.40)			0.84	(0.50–1.41)
2003	0.92	(0.57–1.49)	0.96	(0.57–1.49)	0.97	(0.60–1.56)	0.94	(0.58–1.52)			1.04	(0.62–1.74)
2004	0.94	(0.59–1.52)	1.00	(0.59–1.52)	1.04	(0.64–1.67)	1	ref			1.16	(0.69–1.93)
2005	0.76	(0.47–1.23)	0.79	(0.47–1.23)	0.79	(0.49–1.27)			0.76	(0.48–1.21)	0.76	(0.46–1.27)
2006	0.72	(0.43–1.22)	0.77	(0.43–1.22)	0.78	(0.47–1.31)			0.75	(0.47–1.21)	0.80	(0.46–1.40)
2007	0.86	(0.54–1.37)	0.91	(0.54–1.37)	0.92	(0.58–1.46)			0.90	(0.57–1.41)	1.00	(0.62–1.62)
2008	0.57	(0.33–0.96)	0.60	(0.33–0.96)	0.64	(0.38–1.08)			0.62	(0.38–1.03)	0.66	(0.38–1.15)
2009	0.89	(0.55–1.42)	0.96	(0.55–1.42)	0.99	(0.62–1.59)			0.98	(0.61–1.55)	0.97	(0.59–1.61)
2010	0.56	(0.34–0.91)	0.62	(0.34–0.91)	0.64	(0.40–1.05)			0.64	(0.40–1.03)	0.69	(0.41–1.14)
Previous admissions:												
Yes					4.48	(3.28–6.11)	4.68	(3.14–6.98)	4.20	(2.63–6.71)	4.47	(3.20–6.25)
No					1	ref	1	ref	1	ref	1	ref
Pregancy BMI:												
Underweight											0.55	(0.22–1.36)
Normal											1	ref
Overweight											0.85	(0.66–1.10)

Obese						1.05	(0.78–1.43)
Observations (N):	37,621	37,621	37,621	15,466	22,155	33,236	
Clusters (Maternal ID):	25,344	25,344	25,344	12,377	16,910	23,471	

*Adjusted for year of birth. Analysis included 37,621 children.

†Adjusted for year of birth small for gestational age, large for gestational age, congenital malformation, sex, maternal age, parity, maternal smoking, maternal education level and maternal country of birth. Analysis included 37,621 children.

‡Adjusted for year of birth small for gestational age, large for gestational age, congenital malformation, sex, maternal age, parity, maternal smoking, maternal education level, maternal country of birth and previous admissions. Analysis included 37,621 children.

§Included children born 1998 to 2003. Adjusted for year of birth small for gestational age, large for gestational age, congenital malformation, sex, maternal age, parity, maternal smoking, maternal education level, maternal country of birth and previous admissions. Analysis excluded children born 2004 to 2010, leaving 15,466 children.

¶Included children born 2004 to 2010. Adjusted for year of birth small for gestational age, large for gestational age, congenital malformation, sex, maternal age, parity, maternal smoking, maternal education level, maternal country of birth and previous admissions. Analysis excluded children born 1998 to 2003, leaving 22,155 children.

#Adjusted for year of birth small for gestational age, large for gestational age, congenital malformation, sex, maternal age, parity, maternal smoking, maternal education level, maternal country of birth, previous admissions and pregnancy BMI. Analysis excluded children with missing data on pregnancy BMI, leaving 33,236 children.

Incidence rate ratios were estimated using negative binomial regression models. Correlation between siblings was accounted for using generalized estimating equations with robust standard errors. CI, confidence intervals. BF, breastfeeding. EBF, exclusive breastfeeding. MO, months. SGA, small for gestation age. LGA, large for gestation age. BMI, body mass index.

Table F: Regression results for enteric infections, early childhood (<2 years)

	Crude* (95% CI)		Adjusted† (95% CI)		1998-2003‡ (95% CI)		2004-2010§ (95% CI)		BMI¶ (95% CI)	
Breastfeeding :										
No BF	3.56	(2.30–5.52)	3.32	(2.14–5.14)	3.54	(1.85–6.78)	3.34	(1.85–6.01)	3.17	(1.99–5.05)
EBF <4 MO with BF <6 MO	1.55	(1.24–1.94)	1.50	(1.19–1.89)	1.50	(1.09–2.07)	1.56	(1.11–2.20)	1.59	(1.24–2.05)
EBF <4 MO with BF ≥6 MO	1.44	(1.09–1.91)	1.37	(1.03–1.82)	1.26	(0.82–1.94)	1.52	(1.03–2.25)	1.47	(1.09–1.98)
EBF 4–5 MO with BF <6 MO	1.46	(0.99–2.15)	1.43	(0.97–2.10)	0.99	(0.53–1.86)	2.01	(1.22–3.32)	1.38	(0.89–2.13)
EBF 4–5 MO with BF ≥6 MO	1.03	(0.83–1.27)	1.03	(0.83–1.27)	1.07	(0.81–1.40)	1.03	(0.74–1.43)	1.07	(0.85–1.34)
EBF ≥6 MO	1	ref	1	ref	1	ref	1	ref	1	ref
SGA:										
Yes			1.47	(0.90–2.40)	1.34	(0.63–2.83)	1.58	(0.82–3.03)	1.69	(1.04–2.76)
No			1	ref	1	ref	1	ref	1	ref
LGA:										
Yes			1.01	(0.70–1.44)	1.15	(0.71–1.86)	0.87	(0.51–1.46)	0.91	(0.61–1.35)
No			1	ref	1	ref	1	ref	1	ref
Congenital malformation:										
Yes			2.16	(1.47–3.17)	1.70	(1.02–2.84)	2.87	(1.66–4.95)	2.30	(1.54–3.41)
No			1	ref	1	ref	1	ref	1	ref
Sex										
Male			1	ref	1	ref	1	ref	1	ref
Female			1.05	(0.90–1.23)	0.95	(0.76–1.18)	1.15	(0.92–1.44)	1.02	(0.86–1.20)
Maternal age (years):										
≤19			0.64	(0.29–1.41)	0.76	(0.26–2.18)	0	(0.17–1.75)	0.68	(0.29–1.59)
20-24			1	ref	1	ref	1	ref	1	ref
25-29			1.04	(0.79–1.36)	1.34	(0.93–1.93)	0.83	(0.56–1.22)	1.02	(0.76–1.36)
30-34			0.89	(0.67–1.19)	1.11	(0.74–1.65)	0.75	(0.50–1.12)	0.87	(0.64–1.18)
≥35			0.84	(0.60–1.17)	1.10	(0.69–1.76)	0.68	(0.44–1.08)	0.82	(0.57–1.17)
Parity										
1			1	ref	1	ref	1	ref	1	ref
2			0.89	(0.74–1.07)	0.77	(0.59–1.00)	1.03	(0.79–1.33)	0.91	(0.75–1.11)
3			0.95	(0.73–1.22)	0.91	(0.63–1.29)	0.98	(0.68–1.41)	0.99	(0.75–1.29)
≥4			1.20	(0.81–1.79)	0.80	(0.47–1.35)	1.65	(0.96–2.84)	1.37	(0.91–2.08)
Maternal smoking:										

No smoking			1	ref	1	ref	1	ref	1	ref
Smoking			0.97	(0.71–1.32)	0.60	(0.37–0.97)	1.46	(0.98–2.17)	1.04	(0.76–1.43)
Maternal education (years):										
≤9			1.13	(0.84–1.53)	1.28	(0.84–1.98)	0.99	(0.65–1.50)	1.03	(0.74–1.43)
10-12			0.98	(0.80–1.19)	0.94	(0.71–1.25)	0.96	(0.73–1.27)	0.99	(0.79–1.23)
13-14			0.98	(0.75–1.29)	0.88	(0.61–1.28)	1.02	(0.70–1.51)	0.94	(0.70–1.27)
≥15			1	ref	1	ref	1	ref	1	ref
Maternal country of birth:										
Sweden			1	ref	1	ref	1	ref	1	ref
Other Nordic			1.11	(0.67–1.82)	0.99	(0.49–2.00)	1.24	(0.61–2.52)	1.09	(0.64–1.85)
Europe and North America			1.40	(0.99–1.99)	0.97	(0.56–1.68)	1.82	(1.16–2.85)	1.48	(1.02–2.14)
Asia			1.28	(0.95–1.71)	0.87	(0.54–1.41)	1.63	(1.12–2.37)	1.29	(0.94–1.78)
Africa			2.13	(1.34–3.38)	2.26	(1.22–4.18)	2.10	(1.11–3.96)	2.08	(1.28–3.38)
Other			1.15	(0.58–2.29)	0.90	(0.29–2.77)	1.35	(0.56–3.22)	1.01	(0.45–2.23)
Year of birth:										
1998	1	ref	1	ref	1	ref			1	ref
1999	0.99	(0.67–1.47)	1.01	(0.68–1.48)	1.01	(0.68–1.49)			0.85	(0.56–1.30)
2000	1.30	(0.90–1.86)	1.32	(0.92–1.89)	1.31	(0.91–1.89)			1.27	(0.87–1.84)
2001	0.83	(0.56–1.23)	0.86	(0.57–1.28)	0.85	(0.57–1.27)			0.80	(0.53–1.22)
2002	0.77	(0.52–1.14)	0.78	(0.53–1.16)	0.77	(0.52–1.14)			0.77	(0.51–1.15)
2003	1.13	(0.78–1.62)	1.14	(0.79–1.64)	1.11	(0.77–1.61)			0.89	(0.60–1.33)
2004	1.36	(0.94–1.95)	1.41	(0.98–2.03)			1	ref	1.42	(0.97–2.08)
2005	0.95	(0.65–1.38)	0.98	(0.67–1.44)			0.70	(0.49–1.00)	0.91	(0.61–1.35)
2006	0.68	(0.46–1.02)	0.71	(0.47–1.06)			0.51	(0.35–0.74)	0.67	(0.44–1.01)
2007	0.64	(0.43–0.97)	0.66	(0.44–1.00)			0.47	(0.32–0.70)	0.66	(0.44–1.01)
2008	0.53	(0.35–0.80)	0.54	(0.36–0.83)			0.39	(0.26–0.57)	0.54	(0.35–0.82)
2009	0.59	(0.38–0.90)	0.60	(0.39–0.92)			0.42	(0.28–0.64)	0.57	(0.37–0.89)
2010	0.45	(0.28–0.71)	0.46	(0.29–0.74)			0.33	(0.21–0.51)	0.45	(0.28–0.71)
Pregancy BMI:										
Underweight									0.88	(0.52–1.48)
Normal									1	ref
Overweight									0.85	(0.68–1.05)
Obese									1.10	(0.85–1.42)
Observations (N):	37,825		37,825		15,553		22,272		33,422	
Clusters (Maternal ID):	25,487		25,487		12,445		16,998		23,605	

*Adjusted for year of birth. Analysis included 37,825 children.

†Adjusted for year of birth small for gestational age, large for gestational age, congenital malformation, sex, maternal age, parity, maternal smoking, maternal education level and maternal country of birth. Analysis included 37,825 children.

‡Included children born 1998 to 2003. Adjusted for year of birth small for gestational age, large for gestational age, congenital malformation, sex, maternal age, parity, maternal smoking, maternal education level and maternal country of birth. Analysis excluded children born 2004 to 2010, leaving 15,553 children.

§Included children born 2004 to 2010. Adjusted for year of birth small for gestational age, large for gestational age, congenital malformation, sex, maternal age, parity, maternal smoking, maternal education level and maternal country of birth. Analysis excluded children born 1998 to 2003, leaving 22,272 children.

¶Adjusted for year of birth small for gestational age, large for gestational age, congenital malformation, sex, maternal age, parity, maternal smoking, maternal education level, maternal country of birth and pregnancy BMI. Analysis excluded children with missing data on pregnancy BMI, leaving 33,422 children.

Incidence rate ratios were estimated using negative binomial regression models. Correlation between siblings was accounted for using generalized estimating equations with robust standard errors. CI, confidence intervals. BF, breastfeeding. EBF, exclusive breastfeeding. MO, months. SGA, small for gestation age. LGA, large for gestation age. BMI, body mass index.

Table G: Regression results for enteric infections, later childhood (2–4 years)

	Crude*(95% CI)		Adjusted†(95% CI)		Adjusted 2‡(95% CI)		1998-2003§(95% CI)		2004-2010¶(95% CI)		BMI#(95% CI)	
Breastfeeding:												
No BF	1.34	(0.46–3.91)	1.28	(0.44–3.75)	1.20	(0.41–3.55)	1.91	(0.64–5.73)	-	-	1.06	(0.30–3.70)
EBF <4 MO with BF <6 MO	1.42	(0.99–2.04)	1.36	(0.93–1.99)	1.35	(0.92–1.97)	1.00	(0.61–1.64)	2.77	(1.34–5.73)	1.46	(0.98–2.18)
EBF <4 MO with BF ≥6 MO	1.50	(0.90–2.50)	1.45	(0.86–2.47)	1.45	(0.85–2.47)	1.15	(0.65–2.03)	2.82	(1.02–7.84)	1.51	(0.86–2.66)
EBF 4–5 MO with BF <6 MO	1.15	(0.62–2.13)	1.13	(0.60–2.10)	1.12	(0.60–2.09)	0.98	(0.46–2.09)	1.84	(0.59–5.74)	1.06	(0.53–2.10)
EBF 4–5 MO with BF ≥6 MO	1.15	(0.82–1.60)	1.14	(0.82–1.59)	1.14	(0.82–1.59)	0.96	(0.64–1.43)	2.08	(1.07–4.04)	1.17	(0.82–1.67)
EBF ≥6 MO	1	ref	1	ref	1	ref	1	ref	1	ref	1	ref
SGA												
Yes			2.73	(1.11–6.70)	2.67	(1.08–6.60)	3.17	(0.94–10.60)	1.93	(0.55–6.79)	2.85	(1.16–6.97)
No			1	ref	1	ref	1	ref	1	ref	1	ref
LGA												
Yes			1.06	(0.61–1.82)	1.06	(0.61–1.82)	0.81	(0.38–1.73)	1.51	(0.69–3.29)	1.03	(0.58–1.81)
No			1	ref	1	ref	1	ref	1	ref	1	ref
Congenital malformation:												
Yes			1.97	(1.04–3.71)	1.93	(1.02–3.65)	2.01	(1.00–4.05)	1.56	(0.36–6.83)	2.06	(1.09–3.90)
No			1	ref	1	ref	1	ref	1	ref	1	ref
Sex												
Male			1	ref	1	ref	1	ref	1	ref	1	ref
Female			0.95	(0.73–1.23)	0.95	(0.73–1.23)	0.85	(0.61–1.17)	1.11	(0.73–1.70)	0.92	(0.70–1.20)
Maternal age (years):												
≤19			0.52	(0.15–1.75)	0.53	(0.16–1.77)	0.57	(0.11–2.20)	0.61	(0.08–4.61)	0.57	(0.17–1.95)
20-24			1	ref	1	ref	1	ref	1	ref	1	ref
25-29			0.73	(0.50–1.07)	0.73	(0.50–1.07)	0.61	(0.38–0.98)	1.01	(0.54–1.89)	0.74	(0.49–1.09)
30-34			0.63	(0.41–0.96)	0.63	(0.41–0.97)	0.56	(0.32–0.96)	0.79	(0.40–1.54)	0.62	(0.39–0.96)
≥35			0.56	(0.35–0.92)	0.57	(0.35–0.93)	0.62	(0.33–1.14)	0.53	(0.24–1.14)	0.58	(0.35–0.96)
Parity:												
1			1	ref	1	ref	1	ref	1	ref	1	ref
2			1.05	(0.76–1.45)	1.05	(0.76–1.45)	1.05	(0.70–1.58)	1.05	(0.63–1.76)	1.05	(0.75–1.47)
3			1.07	(0.71–1.61)	1.07	(0.71–1.61)	1.07	(0.62–1.86)	1.11	(0.61–2.00)	1.05	(0.68–1.60)
≥4			1.04	(0.57–1.91)	1.04	(0.57–1.90)	0.83	(0.38–1.84)	1.42	(0.59–3.46)	0.95	(0.50–1.82)
Maternal smoking:												

No smoking			1	ref	1	ref	1	ref	1	ref	1	ref
Smoking			1.04	(0.67–1.61)	1.05	(0.68–1.63)	0.66	(0.35–1.22)	2.02	(1.12–3.66)	1.07	(0.69–1.68)
Maternal education (years):												
≤9			1.05	(0.64–1.72)	1.04	(0.63–1.70)	1.08	(0.58–2.03)	0.91	(0.42–1.95)	1.00	(0.60–1.68)
10-12			0.71	(0.50–1.00)	0.71	(0.50–1.00)	0.62	(0.40–0.97)	0.85	(0.51–1.42)	0.66	(0.45–0.95)
13-14			0.78	(0.52–1.18)	0.78	(0.52–1.19)	0.72	(0.43–1.21)	0.81	(0.41–1.61)	0.71	(0.45–1.10)
≥15			1	ref	1	ref	1	ref	1	ref	1	ref
Maternal country of birth:												
Sweden			1	ref	1	ref	1	ref	1	ref	1	ref
Other Nordic			2.15	(0.71–6.47)	2.14	(0.71–6.46)	1.02	(0.30–3.46)	4.88	(1.06–22.56)	2.31	(0.76–7.04)
Europe and North America			1.68	(1.00–2.84)	1.66	(0.98–2.80)	1.96	(1.04–3.72)	1.12	(0.46–2.72)	1.56	(0.87–2.79)
Asia			0.95	(0.57–1.57)	0.94	(0.57–1.57)	0.60	(0.26–1.39)	1.43	(0.75–2.74)	1.06	(0.63–1.76)
Africa			1.95	(0.89–4.28)	1.90	(0.87–4.18)	2.08	(0.84–5.19)	1.72	(0.40–7.32)	1.89	(0.80–4.45)
Other			0.74	(0.18–3.00)	0.74	(0.18–3.01)	0.58	(0.08–4.19)	1.02	(0.14–7.40)	0.82	(0.20–3.35)
Year of birth:												
1998	1	ref	1	ref	1	ref			1	ref	1	ref
1999	0.85	(0.50–1.47)	0.85	(0.50–1.47)	0.85	(0.49–1.46)	0.85	(0.49–1.46)			0.88	(0.50–1.53)
2000	0.56	(0.32–0.96)	0.56	(0.32–0.96)	0.55	(0.32–0.95)	0.55	(0.32–0.95)			0.52	(0.29–0.92)
2001	0.60	(0.33–1.09)	0.61	(0.33–1.09)	0.62	(0.34–1.12)	0.61	(0.34–1.10)			0.58	(0.30–1.11)
2002	0.89	(0.54–1.48)	0.90	(0.54–1.48)	0.91	(0.55–1.50)	0.88	(0.53–1.45)			0.99	(0.59–1.64)
2003	0.75	(0.46–1.23)	0.75	(0.46–1.23)	0.74	(0.46–1.21)	0.73	(0.45–1.18)			0.76	(0.46–1.27)
2004	0.36	(0.20–0.64)	0.36	(0.20–0.64)	0.36	(0.20–0.64)	1	ref			0.41	(0.23–0.75)
2005	0.43	(0.24–0.75)	0.43	(0.24–0.75)	0.43	(0.24–0.76)			1.27	(0.66–2.46)	0.43	(0.24–0.77)
2006	0.36	(0.20–0.64)	0.36	(0.20–0.64)	0.37	(0.21–0.65)			1.08	(0.55–2.12)	0.35	(0.19–0.64)
2007	0.28	(0.11–0.73)	0.28	(0.11–0.73)	0.28	(0.11–0.72)			0.84	(0.31–2.30)	0.27	(0.10–0.73)
2008	0.33	(0.18–0.59)	0.32	(0.18–0.59)	0.33	(0.18–0.60)			0.98	(0.49–1.94)	0.32	(0.17–0.59)
2009	0.25	(0.13–0.50)	0.24	(0.13–0.50)	0.25	(0.12–0.50)			0.75	(0.34–1.63)	0.23	(0.11–0.48)
2010	0.27	(0.15–0.50)	0.26	(0.15–0.50)	0.27	(0.15–0.50)			0.83	(0.40–1.70)	0.23	(0.12–0.45)
Previous admissions:												
Yes					3.03	(1.83–5.01)	1.96	(0.94–4.08)	5.18	(2.56–10.49)	2.87	(1.68–4.91)
No					1	ref	1	ref	1	ref	1	ref
Pregancy BMI:												
Underweight											0.76	(0.31–1.87)
Normal											1	ref
Overweight											1.22	(0.87–1.71)

Obese						1.17	(0.78–1.75)
Observations (N):	37,621	37,621	37,621	15,466	22,155	33,236	
Clusters (Maternal ID):	25,344	25,344	25,344	12,377	16,910	23,471	

*Adjusted for year of birth. Analysis included 37,621 children.

†Adjusted for year of birth small for gestational age, large for gestational age, congenital malformation, sex, maternal age, parity, maternal smoking, maternal education level and maternal country of birth. Analysis included 37,621 children.

‡Adjusted for year of birth small for gestational age, large for gestational age, congenital malformation, sex, maternal age, parity, maternal smoking, maternal education level, maternal country of birth and previous admissions. Analysis included 37,621 children.

§Included children born 1998 to 2003. Adjusted for year of birth small for gestational age, large for gestational age, congenital malformation, sex, maternal age, parity, maternal smoking, maternal education level, maternal country of birth and previous admissions. Analysis excluded children born 2004 to 2010, leaving 15,466 children.

¶Included children born 2004 to 2010. Adjusted for year of birth small for gestational age, large for gestational age, congenital malformation, sex, maternal age, parity, maternal smoking, maternal education level, maternal country of birth and previous admissions. Analysis excluded children born 1998 to 2003, leaving 22,155 children.

#Adjusted for year of birth small for gestational age, large for gestational age, congenital malformation, sex, maternal age, parity, maternal smoking, maternal education level, maternal country of birth, previous admissions and pregnancy BMI. Analysis excluded children with missing data on pregnancy BMI, leaving 33,236 children.

Incidence rate ratios were estimated using negative binomial regression models. Correlation between siblings was accounted for using generalized estimating equations with robust standard errors. CI, confidence intervals. BF, breastfeeding. EBF, exclusive breastfeeding. MO, months. SGA, small for gestation age. LGA, large for gestation age. BMI, body mass index. The estimate for “No BF” in 2004-2010 is not presented due to few events.

Table H: Regression results for upper respiratory infections, early childhood (<2 years)

	Crude*	(95% CI)	Adjusted†	(95% CI)	1998-2003‡	(95% CI)	2004-2010§	(95% CI)	BMI¶	(95% CI)
Breastfeeding :										
No BF	1.48	(0.88–2.49)	1.31	(0.77–2.22)	1.19	(0.56–2.54)	1.54	(0.74–3.21)	1.24	(0.70–2.18)
EBF <4 MO with BF <6 MO	1.48	(1.19–1.84)	1.40	(1.12–1.75)	1.30	(0.97–1.74)	1.60	(1.11–2.30)	1.36	(1.07–1.74)
EBF <4 MO with BF ≥6 MO	1.22	(0.92–1.62)	1.21	(0.91–1.61)	1.21	(0.80–1.82)	1.28	(0.84–1.96)	1.15	(0.85–1.56)
EBF 4–5 MO with BF <6 MO	0.97	(0.63–1.50)	0.92	(0.60–1.42)	1.01	(0.59–1.73)	0.78	(0.37–1.65)	0.75	(0.46–1.22)
EBF 4–5 MO with BF ≥6 MO	1.18	(0.97–1.44)	1.17	(0.96–1.43)	1.07	(0.82–1.38)	1.36	(0.98–1.90)	1.13	(0.91–1.40)
EBF ≥6 MO	1	ref	1	ref	1	ref	1	ref	1	ref
SGA:										
Yes			1.72	(1.06–2.80)	1.97	(1.04–3.73)	1.47	(0.68–3.14)	1.64	(0.95–2.81)
No			1	ref	1	ref	1	ref	1	ref
LGA:										
Yes			1.31	(0.97–1.75)	1.50	(1.03–2.18)	1.09	(0.67–1.76)	1.24	(0.90–1.71)
No			1	ref	1	ref	1	ref	1	ref
Congenital malformation:										
Yes			2.45	(1.78–3.38)	2.33	(1.54–3.52)	2.63	(1.58–4.37)	2.54	(1.81–3.56)
No			1	ref	1	ref	1	ref	1	ref
Sex										
Male			1	ref	1	ref	1	ref	1	ref
Female			0.74	(0.64–0.86)	0.78	(0.63–0.96)	0.70	(0.56–0.87)	0.74	(0.63–0.87)
Maternal age (years):										
≤19			0.72	(0.33–1.58)	0.54	(0.16–1.77)	0	(0.33–2.75)	0.84	(0.38–1.85)
20-24			1	ref	1	ref	1	ref	1	ref
25-29			1.04	(0.80–1.36)	1.01	(0.70–1.44)	1.10	(0.75–1.61)	1.04	(0.79–1.37)
30-34			0.99	(0.75–1.30)	0.86	(0.58–1.26)	1.16	(0.78–1.71)	0.97	(0.72–1.30)
≥35			0.93	(0.68–1.28)	1.02	(0.65–1.58)	0.84	(0.53–1.33)	0.94	(0.67–1.31)
Parity										
1			1	ref	1	ref	1	ref	1	ref
2			1.15	(0.97–1.37)	1.22	(0.96–1.54)	1.09	(0.85–1.39)	1.15	(0.95–1.38)
3			1.01	(0.78–1.32)	0.98	(0.68–1.43)	1.07	(0.74–1.54)	1.03	(0.79–1.36)
≥4			1.08	(0.77–1.51)	1.06	(0.67–1.67)	1.11	(0.68–1.83)	1.02	(0.71–1.45)
Maternal smoking:										

No smoking			1	ref	1	ref	1	ref	1	ref
Smoking			0.95	(0.70–1.29)	1.01	(0.68–1.50)	0.86	(0.53–1.38)	0.95	(0.69–1.31)
Maternal education (years):										
≤9			1.18	(0.87–1.60)	1.03	(0.66–1.61)	1.38	(0.92–2.06)	1.18	(0.85–1.64)
10-12			1.07	(0.89–1.29)	0.98	(0.75–1.27)	1.18	(0.91–1.53)	1.12	(0.91–1.37)
13-14			0.98	(0.76–1.26)	0.92	(0.65–1.29)	1.03	(0.71–1.50)	0.96	(0.73–1.25)
≥15			1	ref	1	ref	1	ref	1	ref
Maternal country of birth:										
Sweden			1	ref	1	ref	1	ref	1	ref
Other Nordic countries			0.94	(0.58–1.52)	0.96	(0.52–1.76)	0.89	(0.40–1.96)	1.07	(0.66–1.73)
Europe and North America			0.82	(0.54–1.23)	0.96	(0.58–1.61)	0.67	(0.34–1.32)	0.89	(0.58–1.36)
Asia			0.78	(0.55–1.10)	0.73	(0.44–1.21)	0.81	(0.51–1.30)	0.86	(0.60–1.23)
Africa			0.91	(0.52–1.61)	0.76	(0.32–1.79)	1.03	(0.49–2.20)	0.98	(0.54–1.77)
Other			0.53	(0.16–1.72)	0.50	(0.07–3.39)	0.55	(0.14–2.22)	0.46	(0.15–1.44)
Year of birth:										
1998	1	ref	1	ref	1	ref			1	ref
1999	0.71	(0.50–1.01)	0.73	(0.51–1.04)	0.72	(0.51–1.03)			0.68	(0.47–0.99)
2000	0.67	(0.46–0.97)	0.69	(0.48–1.00)	0.68	(0.47–0.99)			0.77	(0.53–1.11)
2001	0.78	(0.57–1.07)	0.83	(0.60–1.14)	0.82	(0.59–1.12)			0.80	(0.57–1.12)
2002	0.74	(0.54–1.01)	0.77	(0.56–1.06)	0.75	(0.55–1.04)			0.75	(0.53–1.05)
2003	0.54	(0.38–0.76)	0.57	(0.40–0.80)	0.56	(0.39–0.79)			0.53	(0.37–0.78)
2004	0.47	(0.33–0.67)	0.50	(0.35–0.71)			1	ref	0.54	(0.38–0.78)
2005	0.51	(0.35–0.72)	0.53	(0.37–0.76)			1.07	(0.73–1.57)	0.57	(0.39–0.82)
2006	0.54	(0.38–0.76)	0.57	(0.40–0.81)			1.13	(0.78–1.66)	0.53	(0.37–0.78)
2007	0.45	(0.31–0.63)	0.48	(0.34–0.67)			0.95	(0.65–1.39)	0.50	(0.35–0.71)
2008	0.32	(0.22–0.46)	0.34	(0.23–0.50)			0.68	(0.45–1.03)	0.32	(0.21–0.48)
2009	0.36	(0.25–0.52)	0.38	(0.26–0.56)			0.77	(0.51–1.15)	0.39	(0.27–0.58)
2010	0.29	(0.20–0.43)	0.32	(0.22–0.48)			0.66	(0.43–1.00)	0.31	(0.20–0.46)
Pregancy BMI:										
Underweight									0.80	(0.43–1.47)
Normal									1	ref
Overweight									0.92	(0.75–1.11)
Obese									1.11	(0.86–1.44)
Observations (N):	37,825		37,825		15,553		22,272		33,422	
Clusters (Maternal ID):	25,487		25,487		12,445		16,998		23,605	

*Adjusted for year of birth. Analysis included 37,825 children.

†Adjusted for year of birth small for gestational age, large for gestational age, congenital malformation, sex, maternal age, parity, maternal smoking, maternal education level and maternal country of birth. Analysis included 37,825 children.

‡Included children born 1998 to 2003. Adjusted for year of birth small for gestational age, large for gestational age, congenital malformation, sex, maternal age, parity, maternal smoking, maternal education level and maternal country of birth. Analysis excluded children born 2004 to 2010, leaving 15,553 children.

§Included children born 2004 to 2010. Adjusted for year of birth small for gestational age, large for gestational age, congenital malformation, sex, maternal age, parity, maternal smoking, maternal education level and maternal country of birth. Analysis excluded children born 1998 to 2003, leaving 22,272 children.

¶Adjusted for year of birth small for gestational age, large for gestational age, congenital malformation, sex, maternal age, parity, maternal smoking, maternal education level, maternal country of birth and pregnancy BMI. Analysis excluded children with missing data on pregnancy BMI, leaving 33,422 children.

Incidence rate ratios were estimated using negative binomial regression models. Correlation between siblings was accounted for using generalized estimating equations with robust standard errors. CI, confidence intervals. BF, breastfeeding. EBF, exclusive breastfeeding. MO, months. SGA, small for gestation age. LGA, large for gestation age. BMI, body mass index.

Table I: Regression results for lower respiratory infections, early childhood (<2 years)

	Crude[*](95% CI)		Adjusted[†](95% CI)		1998-2003[‡](95% CI)		2004-2010[§](95% CI)		BMI[¶](95% CI)	
Breastfeeding :										
No BF	2.39	(1.38–4.13)	2.08	(1.21–3.59)	4.03	(1.79–9.09)	1.25	(0.64–2.45)	2.14	(1.21–3.77)
EBF <4 MO with BF <6 MO	1.36	(1.06–1.76)	1.39	(1.07–1.79)	1.57	(1.01–2.44)	1.28	(0.94–1.76)	1.29	(0.97–1.70)
EBF <4 MO with BF ≥6 MO	1.34	(0.99–1.81)	1.38	(1.02–1.86)	1.48	(0.86–2.57)	1.29	(0.90–1.84)	1.27	(0.92–1.75)
EBF 4–5 MO with BF <6 MO	1.07	(0.63–1.79)	1.09	(0.65–1.81)	1.12	(0.41–3.07)	1.07	(0.61–1.87)	1.04	(0.60–1.82)
EBF 4–5 MO with BF ≥6 MO	0.97	(0.77–1.22)	0.99	(0.79–1.25)	1.15	(0.77–1.72)	0.90	(0.69–1.19)	0.95	(0.75–1.20)
EBF ≥6 MO	1	ref	1	ref	1	ref	1	ref	1	ref
SGA:										
Yes			2.02	(0.96–4.25)	1.52	(0.42–5.57)	2.28	(0.94–5.56)	2.29	(1.09–4.84)
No			1	ref	1	ref	1	ref	1	ref
LGA:										
Yes			1.29	(0.90–1.84)	1.50	(0.83–2.72)	1.18	(0.76–1.83)	1.31	(0.90–1.91)
No			1	ref	1	ref	1	ref	1	ref
Congenital malformation:										
Yes			2.29	(1.50–3.51)	3.26	(1.92–5.54)	1.43	(0.72–2.84)	2.21	(1.40–3.48)
No			1	ref	1	ref	1	ref	1	ref
Sex										
Male			1	ref	1	ref	1	ref	1	ref
Female			0.65	(0.55–0.78)	0.78	(0.56–1.08)	0.59	(0.48–0.73)	0.64	(0.53–0.77)
Maternal age (years):										
≤19			1.48	(0.61–3.58)	1.19	(0.27–5.26)	0	(0.58–5.07)	1.56	(0.59–4.12)
20-24			1	ref	1	ref	1	ref	1	ref
25-29			1.07	(0.77–1.50)	1.38	(0.74–2.56)	0.96	(0.65–1.44)	1.12	(0.78–1.61)
30-34			1.15	(0.82–1.62)	1.52	(0.80–2.87)	1.02	(0.68–1.51)	1.21	(0.83–1.76)
≥35			1.04	(0.71–1.51)	1.40	(0.69–2.84)	0.90	(0.58–1.41)	1.10	(0.73–1.67)
Parity										
1			1	ref	1	ref	1	ref	1	ref
2			2.01	(1.62–2.49)	1.54	(1.03–2.31)	2.29	(1.79–2.94)	1.94	(1.54–2.44)
3			1.99	(1.51–2.62)	1.40	(0.82–2.41)	2.37	(1.74–3.23)	1.91	(1.42–2.56)
≥4			2.60	(1.75–3.87)	2.55	(1.32–4.89)	2.58	(1.58–4.21)	2.71	(1.80–4.08)
Maternal smoking:										

No smoking			1	ref	1	ref	1	ref	1	ref
Smoking			1.47	(1.07–2.02)	1.53	(0.91–2.59)	1.40	(0.94–2.08)	1.43	(1.00–2.03)
Maternal education (years):										
≤9			0.83	(0.58–1.18)	0.91	(0.50–1.63)	0.79	(0.51–1.24)	0.80	(0.55–1.17)
10-12			0.89	(0.73–1.09)	0.96	(0.64–1.45)	0.87	(0.69–1.10)	0.86	(0.69–1.07)
13-14			0.89	(0.68–1.18)	0.94	(0.58–1.51)	0.88	(0.62–1.26)	0.93	(0.70–1.25)
≥15			1	ref	1	ref	1	ref	1	ref
Maternal country of birth:										
Sweden			1	ref	1	ref	1	ref	1	ref
Other Nordic countries			1.15	(0.67–1.95)	1.18	(0.50–2.77)	1.13	(0.57–2.24)	1.03	(0.59–1.82)
Europe and North America			0.54	(0.30–0.96)	0.24	(0.06–0.97)	0.67	(0.36–1.25)	0.61	(0.34–1.08)
Asia			0.86	(0.59–1.26)	0.75	(0.36–1.57)	0.90	(0.59–1.39)	0.82	(0.53–1.26)
Africa			1.44	(0.88–2.36)	1.08	(0.39–2.97)	1.62	(0.92–2.86)	1.53	(0.91–2.55)
Other			1.20	(0.61–2.40)	1.60	(0.52–4.93)	1.08	(0.45–2.56)	0.84	(0.35–1.99)
Year of birth:										
1998	1	ref	1	ref	1	ref			1	ref
1999	1.21	(0.66–2.23)	1.26	(0.68–2.31)	1.27	(0.69–2.33)			1.44	(0.77–2.68)
2000	1.49	(0.85–2.64)	1.55	(0.88–2.73)	1.54	(0.87–2.70)			1.65	(0.90–3.02)
2001	1.53	(0.90–2.59)	1.60	(0.94–2.72)	1.59	(0.94–2.71)			1.62	(0.91–2.87)
2002	1.36	(0.80–2.33)	1.42	(0.83–2.43)	1.41	(0.82–2.41)			1.47	(0.82–2.63)
2003	1.41	(0.84–2.37)	1.50	(0.89–2.52)	1.51	(0.89–2.54)			1.49	(0.86–2.59)
2004	1.56	(0.97–2.53)	1.64	(1.02–2.65)			1	ref	1.80	(1.08–3.00)
2005	2.32	(1.45–3.72)	2.42	(1.51–3.87)			1.48	(1.01–2.16)	2.64	(1.61–4.34)
2006	1.45	(0.88–2.37)	1.52	(0.93–2.48)			0.93	(0.62–1.40)	1.51	(0.89–2.56)
2007	2.29	(1.44–3.64)	2.41	(1.52–3.83)			1.46	(1.01–2.11)	2.34	(1.42–3.86)
2008	1.54	(0.94–2.50)	1.63	(1.00–2.65)			1.00	(0.67–1.48)	1.65	(0.99–2.75)
2009	1.98	(1.22–3.22)	2.10	(1.31–3.38)			1.28	(0.87–1.89)	2.14	(1.29–3.53)
2010	1.87	(1.17–2.98)	2.03	(1.27–3.24)			1.22	(0.84–1.78)	2.03	(1.23–3.34)
Pregancy BMI:										
Underweight									1.33	(0.75–2.37)
Normal									1	ref
Overweight									0.97	(0.78–1.22)
Obese									1.22	(0.93–1.61)
Observations (N):	37,825		37,825		15,553		22,272		33,422	
Clusters (Maternal ID):	25,487		25,487		12,445		16,998		23,605	

*Adjusted for year of birth. Analysis included 37,825 children.

†Adjusted for year of birth small for gestational age, large for gestational age, congenital malformation, sex, maternal age, parity, maternal smoking, maternal education level and maternal country of birth. Analysis included 37,825 children.

‡Included children born 1998 to 2003. Adjusted for year of birth small for gestational age, large for gestational age, congenital malformation, sex, maternal age, parity, maternal smoking, maternal education level and maternal country of birth. Analysis excluded children born 2004 to 2010, leaving 15,553 children.

§Included children born 2004 to 2010. Adjusted for year of birth small for gestational age, large for gestational age, congenital malformation, sex, maternal age, parity, maternal smoking, maternal education level and maternal country of birth. Analysis excluded children born 1998 to 2003, leaving 22,272 children.

¶Adjusted for year of birth small for gestational age, large for gestational age, congenital malformation, sex, maternal age, parity, maternal smoking, maternal education level, maternal country of birth and pregnancy BMI. Analysis excluded children with missing data on pregnancy BMI, leaving 33,422 children.

Incidence rate ratios were estimated using negative binomial regression models. Correlation between siblings was accounted for using generalized estimating equations with robust standard errors. CI, confidence intervals. BF, breastfeeding. EBF, exclusive breastfeeding. MO, months. SGA, small for gestation age. LGA, large for gestation age. BMI, body mass index.

Table J: Regression results for upper respiratory infections, later childhood (2–4 years)

	Crude[*]	(95% CI)	Adjusted[†]	(95% CI)	Adjusted 2[‡]	(95% CI)	1998-2003[§]	(95% CI)	2004-2010[¶]	(95% CI)	BMI[#]	(95% CI)
Breastfeeding:												
No BF	2.50	(1.30–4.81)	2.25	(1.17–4.33)	2.25	(1.17–4.34)	4.04	(1.82–8.94)	0.93	(0.29–3.00)	2.16	(1.11–4.19)
EBF <4 MO with BF <6 MO	1.38	(0.97–1.95)	1.30	(0.91–1.86)	1.27	(0.88–1.81)	1.65	(1.01–2.68)	0.93	(0.55–1.55)	1.31	(0.89–1.94)
EBF <4 MO with BF ≥6 MO	1.47	(0.99–2.18)	1.45	(0.97–2.15)	1.42	(0.96–2.11)	1.41	(0.78–2.54)	1.35	(0.79–2.29)	1.52	(1.00–2.30)
EBF 4–5 MO with BF <6 MO	1.35	(0.76–2.41)	1.33	(0.74–2.37)	1.34	(0.75–2.41)	0.87	(0.37–2.04)	1.90	(0.86–4.19)	1.45	(0.79–2.68)
EBF 4–5 MO with BF ≥6 MO	1.22	(0.90–1.64)	1.22	(0.90–1.64)	1.21	(0.90–1.63)	1.23	(0.82–1.85)	1.15	(0.74–1.77)	1.23	(0.89–1.69)
EBF ≥6 MO	1	ref	1	ref	1	ref	1	ref	1	ref	1	ref
SGA												
Yes			1.22	(0.58–2.59)	1.19	(0.56–2.53)	1.27	(0.48–3.35)	1.13	(0.36–3.54)	1.20	(0.53–2.71)
No			1	ref	1	ref	1	ref	1	ref	1	ref
LGA												
Yes			1.33	(0.84–2.09)	1.29	(0.82–2.04)	1.19	(0.59–2.37)	1.42	(0.79–2.56)	1.27	(0.80–2.03)
No			1	ref	1	ref	1	ref	1	ref	1	ref
Congenital malformation:												
Yes			2.73	(1.72–4.35)	2.47	(1.56–3.91)	2.33	(1.26–4.28)	2.52	(1.26–5.05)	2.45	(1.49–4.03)
No			1	ref	1	ref	1	ref	1	ref	1	ref
Sex												
Male			1	ref	1	ref	1	ref	1	ref	1	ref
Female			0.67	(0.53–0.84)	0.68	(0.54–0.86)	0.76	(0.55–1.06)	0.61	(0.44–0.84)	0.70	(0.55–0.90)
Maternal age (years):												
≤19			0.38	(0.09–1.57)	0.40	(0.10–1.64)	0.49	(0.04–2.08)	0.60	(0.08–4.60)	0.49	(0.12–2.03)
20-24			1	ref	1	ref	1	ref	1	ref	1	ref
25-29			1.04	(0.71–1.51)	1.04	(0.71–1.52)	0.93	(0.54–1.59)	1.20	(0.69–2.09)	1.08	(0.72–1.63)
30-34			0.92	(0.63–1.36)	0.94	(0.63–1.39)	1.11	(0.65–1.90)	0.81	(0.46–1.43)	1.01	(0.66–1.54)
≥35			1.15	(0.74–1.77)	1.16	(0.75–1.80)	1.23	(0.68–2.22)	1.10	(0.58–2.12)	1.23	(0.77–1.96)
Parity:												
1			1	ref	1	ref	1	ref	1	ref	1	ref
2			1.04	(0.81–1.34)	1.03	(0.80–1.33)	0.93	(0.64–1.33)	1.17	(0.83–1.66)	1.07	(0.81–1.40)
3			0.81	(0.56–1.17)	0.80	(0.56–1.16)	0.57	(0.34–0.97)	1.10	(0.66–1.84)	0.79	(0.53–1.18)
≥4			0.98	(0.59–1.64)	0.98	(0.58–1.63)	0.81	(0.41–1.62)	1.17	(0.54–2.52)	1.09	(0.65–1.85)
Maternal smoking:												

No smoking			1	ref	1	ref	1	ref	1	ref	1	ref
Smoking			1.19	(0.79–1.78)	1.20	(0.80–1.80)	1.15	(0.69–1.89)	1.21	(0.62–2.37)	1.04	(0.66–1.62)
Maternal education (years):												
≤9			1.25	(0.84–1.87)	1.24	(0.83–1.85)	1.51	(0.87–2.61)	0.97	(0.51–1.84)	1.30	(0.85–2.00)
10-12			0.97	(0.73–1.27)	0.97	(0.73–1.27)	1.07	(0.71–1.63)	0.90	(0.62–1.30)	1.04	(0.77–1.41)
13-14			1.23	(0.87–1.72)	1.24	(0.88–1.73)	1.12	(0.68–1.85)	1.44	(0.92–2.27)	1.35	(0.94–1.92)
≥15			1	ref	1	ref	1	ref	1	ref	1	ref
Maternal country of birth:												
Sweden			1	ref	1	ref	1	ref	1	ref	1	ref
Other Nordic countries			0.39	(0.13–1.22)	0.39	(0.12–1.20)	0.47	(0.12–1.89)	0.29	(0.04–2.07)	0.42	(0.14–1.31)
Europe and North America			0.82	(0.44–1.53)	0.83	(0.45–1.55)	0.92	(0.36–2.35)	0.75	(0.33–1.71)	0.62	(0.31–1.26)
Asia			0.84	(0.53–1.34)	0.87	(0.54–1.38)	0.87	(0.46–1.64)	0.87	(0.45–1.68)	0.88	(0.55–1.42)
Africa			0.62	(0.23–1.64)	0.60	(0.23–1.58)	0.70	(0.19–2.60)	0.55	(0.13–2.24)	0.66	(0.25–1.73)
Other			1.37	(0.58–3.27)	1.39	(0.59–3.26)	2.37	(0.93–6.02)	0.54	(0.08–3.79)	1.27	(0.48–3.36)
Year of birth:												
1998	1	ref	1	ref	1	ref			1	ref	1	ref
1999	1.05	(0.61–1.80)	1.09	(0.61–1.80)	1.13	(0.66–1.92)	1.12	(0.66–1.91)			1.20	(0.68–2.10)
2000	1.15	(0.68–1.96)	1.20	(0.68–1.96)	1.23	(0.73–2.09)	1.22	(0.72–2.07)			1.28	(0.73–2.25)
2001	0.94	(0.56–1.59)	0.99	(0.56–1.59)	1.00	(0.60–1.68)	0.98	(0.59–1.64)			1.00	(0.57–1.74)
2002	0.69	(0.40–1.19)	0.71	(0.40–1.19)	0.73	(0.42–1.27)	0.73	(0.42–1.26)			0.64	(0.35–1.18)
2003	0.77	(0.42–1.39)	0.81	(0.42–1.39)	0.84	(0.46–1.52)	0.82	(0.45–1.49)			0.94	(0.50–1.79)
2004	0.83	(0.49–1.41)	0.88	(0.49–1.41)	0.93	(0.55–1.58)	1	ref			1.05	(0.60–1.84)
2005	0.60	(0.34–1.06)	0.63	(0.34–1.06)	0.66	(0.37–1.17)			0.71	(0.41–1.23)	0.68	(0.37–1.24)
2006	0.69	(0.40–1.20)	0.73	(0.40–1.20)	0.76	(0.44–1.32)			0.81	(0.49–1.36)	0.82	(0.46–1.46)
2007	0.74	(0.43–1.28)	0.80	(0.43–1.28)	0.84	(0.49–1.45)			0.91	(0.55–1.53)	0.92	(0.52–1.62)
2008	0.38	(0.20–0.72)	0.40	(0.20–0.72)	0.43	(0.23–0.82)			0.48	(0.26–0.89)	0.48	(0.25–0.93)
2009	0.63	(0.36–1.09)	0.68	(0.36–1.09)	0.73	(0.42–1.27)			0.81	(0.48–1.37)	0.66	(0.36–1.19)
2010	0.48	(0.27–0.86)	0.53	(0.27–0.86)	0.57	(0.32–1.01)			0.63	(0.36–1.10)	0.61	(0.34–1.12)
Previous admissions:												
Yes					4.52	(3.02–6.78)	4.27	(2.66–6.88)	4.94	(2.47–9.89)	4.16	(2.63–6.57)
No					1	ref	1	ref	1	ref	1	ref
Pregancy BMI:												
Underweight											0.44	(0.14–1.34)
Normal											1	ref
Overweight											0.95	(0.73–1.25)

Obese						1.04	(0.71–1.53)
Observations (N):	37,621	37,621	37,621	15,466	22,155	33,236	
Clusters (Maternal ID):	25,344	25,344	25,344	12,377	16,910	23,471	

*Adjusted for year of birth. Analysis included 37,621 children.

†Adjusted for year of birth small for gestational age, large for gestational age, congenital malformation, sex, maternal age, parity, maternal smoking, maternal education level and maternal country of birth. Analysis included 37,621 children.

‡Adjusted for year of birth small for gestational age, large for gestational age, congenital malformation, sex, maternal age, parity, maternal smoking, maternal education level, maternal country of birth and previous admissions. Analysis included 37,621 children.

§Included children born 1998 to 2003. Adjusted for year of birth small for gestational age, large for gestational age, congenital malformation, sex, maternal age, parity, maternal smoking, maternal education level, maternal country of birth and previous admissions. Analysis excluded children born 2004 to 2010, leaving 15,466 children.

¶Included children born 2004 to 2010. Adjusted for year of birth small for gestational age, large for gestational age, congenital malformation, sex, maternal age, parity, maternal smoking, maternal education level, maternal country of birth and previous admissions. Analysis excluded children born 1998 to 2003, leaving 22,155 children.

#Adjusted for year of birth small for gestational age, large for gestational age, congenital malformation, sex, maternal age, parity, maternal smoking, maternal education level, maternal country of birth, previous admissions and pregnancy BMI. Analysis excluded children with missing data on pregnancy BMI, leaving 33,236 children.

Incidence rate ratios were estimated using negative binomial regression models. Correlation between siblings was accounted for using generalized estimating equations with robust standard errors. CI, confidence intervals. BF, breastfeeding. EBF, exclusive breastfeeding. MO, months. SGA, small for gestation age. LGA, large for gestation age. BMI, body mass index.

Table K: Regression results for lower respiratory infections, later childhood (2–4 years)

	Crude*	(95% CI)	Adjusted†	(95% CI)	Adjusted 2‡	(95% CI)	1998-2003§	(95% CI)	2004-2010¶	(95% CI)	BMI#	(95% CI)
Breastfeeding:												
No BF	3.70	(1.76–7.78)	3.50	(1.69–7.24)	3.10	(1.49–6.45)	5.43	(2.33-12.65)	1.28	(0.27–6.12)	3.33	(1.56–7.11)
EBF <4 MO with BF <6 MO	1.79	(1.12–2.86)	1.93	(1.18–3.15)	1.87	(1.15–3.06)	1.62	(0.76–3.44)	2.04	(1.06–3.91)	1.81	(1.07–3.08)
EBF <4 MO with BF ≥6 MO	1.18	(0.70–2.00)	1.15	(0.67–1.96)	1.13	(0.66–1.93)	1.54	(0.71–3.36)	0.96	(0.47–1.98)	1.15	(0.66–2.03)
EBF 4–5 MO with BF <6 MO	0.46	(0.16–1.29)	0.51	(0.18–1.42)	0.50	(0.18–1.40)	0.86	(0.25–2.91)	0.24	(0.03–1.78)	0.54	(0.19–1.53)
EBF 4–5 MO with BF ≥6 MO	0.85	(0.56–1.28)	0.87	(0.58–1.31)	0.88	(0.58–1.32)	0.90	(0.48–1.67)	0.88	(0.51–1.51)	0.86	(0.56–1.32)
EBF ≥6 MO	1	ref	1	ref	1	ref	1	ref	1	ref	1	ref
SGA												
Yes			3.89	(1.86–8.15)	3.86	(1.84–8.10)	1.93	(0.57–6.50)	5.43	(2.29-12.85)	4.17	(1.99–8.74)
No			1	ref	1	ref	1	ref	1	ref	1	ref
LGA												
Yes			0.99	(0.53–1.85)	0.99	(0.53–1.88)	0.87	(0.32–2.40)	1.04	(0.47–2.31)	1.01	(0.51–2.01)
No			1	ref	1	ref	1	ref	1	ref	1	ref
Congenital malformation:												
Yes			5.40	(3.31–8.81)	4.91	(2.96–8.12)	4.47	(2.07–9.66)	4.75	(2.45–9.21)	4.43	(2.62–7.49)
No			1	ref	1	ref	1	ref	1	ref	1	ref
Sex												
Male			1	ref	1	ref	1	ref	1	ref	1	ref
Female			1.00	(0.73–1.36)	1.03	(0.75–1.42)	1.25	(0.78–2.00)	0.87	(0.58–1.31)	1.04	(0.75–1.46)
Maternal age (years):												
≤19			1.10	(0.23–5.16)	1.12	(0.24–5.29)	1.29	(0.27-23.97)	0.82	(0.09–7.22)	1.29	(0.27–6.24)
20-24			1	ref	1	ref	1	ref	1	ref	1	ref
25-29			1.57	(0.87–2.83)	1.53	(0.85–2.76)	2.68	(0.91–7.84)	1.22	(0.60–2.50)	1.66	(0.89–3.09)
30-34			1.38	(0.77–2.46)	1.34	(0.75–2.39)	4.01	(1.37-11.79)	0.69	(0.34–1.36)	1.45	(0.79–2.66)
≥35			1.97	(1.03–3.76)	1.94	(1.02–3.70)	6.07	(1.86-19.81)	0.98	(0.46–2.10)	2.01	(1.02–3.95)
Parity:												
1			1	ref	1	ref	1	ref	1	ref	1	ref
2			1.03	(0.72–1.47)	1.00	(0.69–1.43)	0.76	(0.44–1.30)	1.33	(0.85–2.09)	0.96	(0.66–1.40)
3			1.01	(0.58–1.76)	0.97	(0.55–1.71)	0.55	(0.22–1.39)	1.59	(0.79–3.18)	0.95	(0.51–1.76)
≥4			0.83	(0.42–1.66)	0.79	(0.39–1.60)	0.44	(0.18–1.10)	1.32	(0.50–3.48)	0.79	(0.38–1.65)
Maternal smoking:												

No smoking			1	ref	1	ref	1	ref	1	ref	1	ref
Smoking			0.53	(0.28–1.00)	0.51	(0.27–0.96)	0.52	(0.22–1.22)	0.52	(0.21–1.31)	0.45	(0.23–0.89)
Maternal education (years):												
≤9			0.92	(0.48–1.76)	0.91	(0.47–1.75)	0.88	(0.37–2.11)	0.82	(0.33–2.07)	1.01	(0.51–2.01)
10-12			0.95	(0.65–1.39)	0.95	(0.65–1.39)	1.23	(0.68–2.22)	0.75	(0.46–1.23)	1.00	(0.66–1.50)
13-14			0.99	(0.62–1.59)	0.99	(0.62–1.59)	0.90	(0.45–1.82)	1.07	(0.57–2.03)	0.98	(0.60–1.61)
≥15			1	ref	1	ref	1	ref	1	ref	1	ref
Maternal country of birth:												
Sweden			1	ref	1	ref	1	ref	1	ref	1	ref
Other Nordic countries			1.58	(0.74–3.36)	1.55	(0.73–3.31)	1.42	(0.45–4.51)	1.69	(0.62–4.60)	1.43	(0.63–3.25)
Europe and North America			0.22	(0.05–0.87)	0.22	(0.06–0.90)	0.33	(0.05–2.45)	0.17	(0.02–1.23)	0.24	(0.06–0.98)
Sweden			1.40	(0.79–2.47)	1.41	(0.80–2.51)	1.40	(0.62–3.16)	1.30	(0.58–2.91)	1.57	(0.88–2.80)
Africa			2.05	(0.69–6.06)	2.02	(0.69–5.95)	2.34	(0.79–6.92)	1.77	(0.34–9.14)	1.87	(0.56–6.18)
Other			1.63	(0.37–7.15)	1.64	(0.37–7.30)	3.09	(0.51-18.82)	0.69	(0.10–4.95)	0.50	(0.07–3.61)
Year of birth:												
1998	1	ref	1	ref	1	ref			1	ref	1	ref
1999	0.93	(0.37–2.38)	0.99	(0.37–2.38)	0.99	(0.39–2.53)	0.99	(0.39–2.51)			1.06	(0.40–2.80)
2000	1.26	(0.50–3.13)	1.34	(0.50–3.13)	1.30	(0.53–3.22)	1.21	(0.50–2.91)			1.36	(0.53–3.50)
2001	1.20	(0.46–3.12)	1.27	(0.46–3.12)	1.25	(0.48–3.20)	1.14	(0.46–2.83)			1.06	(0.38–3.02)
2002	1.22	(0.51–2.93)	1.24	(0.51–2.93)	1.19	(0.50–2.86)	1.08	(0.45–2.59)			1.21	(0.48–3.03)
2003	1.29	(0.56–2.96)	1.30	(0.56–2.96)	1.24	(0.54–2.84)	1.13	(0.50–2.58)			1.23	(0.51–2.98)
2004	1.19	(0.47–3.01)	1.25	(0.47–3.01)	1.21	(0.48–3.05)	1	ref			1.31	(0.49–3.49)
2005	1.14	(0.48–2.70)	1.15	(0.48–2.70)	1.06	(0.45–2.49)			0.89	(0.42–1.87)	0.95	(0.39–2.32)
2006	0.80	(0.28–2.27)	0.83	(0.28–2.27)	0.81	(0.28–2.33)			0.67	(0.26–1.74)	0.77	(0.25–2.42)
2007	1.14	(0.49–2.62)	1.14	(0.49–2.62)	1.09	(0.47–2.53)			0.89	(0.43–1.87)	1.19	(0.50–2.83)
2008	1.00	(0.40–2.53)	1.04	(0.40–2.53)	1.02	(0.41–2.54)			0.85	(0.37–1.97)	0.99	(0.38–2.61)
2009	1.46	(0.64–3.32)	1.55	(0.64–3.32)	1.47	(0.65–3.36)			1.23	(0.59–2.59)	1.55	(0.66–3.63)
2010	0.74	(0.31–1.77)	0.83	(0.31–1.77)	0.77	(0.32–1.87)			0.64	(0.29–1.44)	0.79	(0.32–1.97)
Previous admissions:												
Yes					6.29	(3.44-11.50)	9.65	(3.41-27.27)	4.23	(2.15–8.32)	6.58	(3.50-12.38)
No					1	ref	1	ref	1	ref	1	ref
Pregancy BMI:												
Underweight											0.72	(0.21–2.48)
Normal											1	ref
Overweight											0.68	(0.42–1.11)

Obese											1.06	(0.68–1.65)
Observations (N):	37,621		37,621		37,621		15,466		22,155		33,236	
Clusters (Maternal ID):	25,344		25,344		25,344		12,377		16,910		23,471	

*Adjusted for year of birth. Analysis included 37,621 children.

†Adjusted for year of birth small for gestational age, large for gestational age, congenital malformation, sex, maternal age, parity, maternal smoking, maternal education level and maternal country of birth. Analysis included 37,621 children.

‡Adjusted for year of birth small for gestational age, large for gestational age, congenital malformation, sex, maternal age, parity, maternal smoking, maternal education level, maternal country of birth and previous admissions. Analysis included 37,621 children.

§Included children born 1998 to 2003. Adjusted for year of birth small for gestational age, large for gestational age, congenital malformation, sex, maternal age, parity, maternal smoking, maternal education level, maternal country of birth and previous admissions. Analysis excluded children born 2004 to 2010, leaving 15,466 children.

¶Included children born 2004 to 2010. Adjusted for year of birth small for gestational age, large for gestational age, congenital malformation, sex, maternal age, parity, maternal smoking, maternal education level, maternal country of birth and previous admissions. Analysis excluded children born 1998 to 2003, leaving 22,155 children.

#Adjusted for year of birth small for gestational age, large for gestational age, congenital malformation, sex, maternal age, parity, maternal smoking, maternal education level, maternal country of birth, previous admissions and pregnancy BMI. Analysis excluded children with missing data on pregnancy BMI, leaving 33,236 children.

Incidence rate ratios were estimated using negative binomial regression models. Correlation between siblings was accounted for using generalized estimating equations with robust standard errors. CI, confidence intervals. BF, breastfeeding. EBF, exclusive breastfeeding. MO, months. SGA, small for gestation age. LGA, large for gestation age. BMI, body mass index.
