# Supplementary material for: Breastfeeding practice, breastfeeding policy and hospitalisations for infectious diseases in early and later childhood: a register-based study in Uppsala County, Sweden
Source: BMJ Open. 2021 May 30;11(5):e046583. doi: 10.1136/bmjopen-2020-046583 (PMC8169467; doi:10.1136/bmjopen-2020-046583)
Supplement: Author's manuscript [file bmjopen-2020-046583.draft_revisions.pdf]

BMJ Open

BMJ Open is committed to open peer review. As part of this commitment we make the peer review history of every article we publish publicly available.

When an article is published we post the peer reviewers' comments and the authors' responses online. We also post the versions of the paper that were used during peer review. These are the versions that the peer review comments apply to.

The versions of the paper that follow are the versions that were submitted during the peer review process. They are not the versions of record or the final published versions. They should not be cited or distributed as the published version of this manuscript.

BMJ Open is an open access journal and the full, final, typeset and author-corrected version of record of the manuscript is available on our site with no access controls, subscription charges or pay-per-view fees (http://bmjopen.bmj.com).

If you have any questions on BMJ Open's open peer review process please email info.bmjopen@bmj.com

BMJ Open

Breastfeeding practices and childhood infections: a cohort study

Journal:	*BMJ Open*
Manuscript ID	bmjopen-2020-046583
Article Type:	Original research
Date Submitted by the Author:	03-Nov-2020
Complete List of Authors:	Videholm, Samuel; Umeå universitet Klinisk vetenskap, unit of Pediatrics<br>Wallby, Thomas; Uppsala University Department of Womens and Childrens Health<br>Silfverdal, Sven-Arne; Umeå universitet Klinisk vetenskap, unit of Pediatrics
Keywords:	INFECTIOUS DISEASES, Nutrition < TROPICAL MEDICINE, Public health < INFECTIOUS DISEASES, EPIDEMIOLOGY, Community child health < PAEDIATRICS

SCHOLARONE™
Manuscripts

I, the Submitting Author has the right to grant and does grant on behalf of all authors of the Work (as defined in the below author licence), an exclusive licence and/or a non-exclusive licence for contributions from authors who are: i) UK Crown employees; ii) where BMJ has agreed a CC-BY licence shall apply, and/or iii) in accordance with the terms applicable for US Federal Government officers or employees acting as part of their official duties; on a worldwide, perpetual, irrevocable, royalty-free basis to BMJ Publishing Group Ltd ("BMJ") its licensees and where the relevant Journal is co-owned by BMJ to the co-owners of the Journal, to publish the Work in this journal and any other BMJ products and to exploit all rights, as set out in our licence.

The Submitting Author accepts and understands that any supply made under these terms is made by BMJ to the Submitting Author unless you are acting as an employee on behalf of your employer or a postgraduate student of an affiliated institution which is paying any applicable article publishing charge ("APC") for Open Access articles. Where the Submitting Author wishes to make the Work available on an Open Access basis (and intends to pay the relevant APC), the terms of reuse of such Open Access shall be governed by a Creative Commons licence – details of these licences and which Creative Commons licence will apply to this Work are set out in our licence referred to above.

Other than as permitted in any relevant BMJ Author's Self Archiving Policies, I confirm this Work has not been accepted for publication elsewhere, is not being considered for publication elsewhere and does not duplicate material already published. I confirm all authors consent to publication of this Work and authorise the granting of this licence.

For peer review only

Breastfeeding practices and childhood infections: a cohort study

Samuel Videholm, MD[1], Thomas Wallby, PhD[2], Sven-Arne Silfverdal, MD, PhD, MPH[1]

[1]Department of Clinical Sciences, Pediatrics, Umeå University, Umeå, Sweden

[2]Department of Women's and Children's Health, Uppsala University, Uppsala, Sweden

Corresponding Author:

Name: Samuel Videholm

E-mail: samuel.videholm@umu.se

Telephone number: +46 907858919

Fax: +46 90123728

Postal address:

Department of Clinical Sciences, Pediatrics,

Umeå University

901 87 Umeå

Sweden

Keywords:

Infectious Diseases

Breastfeeding

Public Health

Epidemiology

Community Child Health

Word count: 2716

For peer review only - http://bmjopen.bmj.com/site/about/guidelines.xhtml

For peer review only

Abstract

Objective: To examine the association between breastfeeding practices and hospitalisations for infectious diseases in the first five years of life.

Design: A register-based cohort study.

Setting: A cohort was created by combining the Swedish Medical Birth Register, the National Inpatient Register, the Cause of Death Register, the Total Population Register, the Longitudinal integration database for health insurance and labour market studies, with the Uppsala Preventive Child Health Care database.

Patients: 37 825 full-term singletons born to women who resided in Uppsala County (Sweden) between 1998 and 2010.

Main outcome measures: Number of hospitalisations for infectious diseases in early (<2 years) and later childhood (2–4 years).

Results: The risk of hospitalisations for infectious diseases decreased with duration of exclusive breastfeeding until 4 months of age. In early childhood, breastfeeding was associated with a decreased risk of enteric and respiratory infections. The strongest association was found between no breastfeeding and enteric infections (aIRR 3.32 [95% CI 2.14–5.14]). In later childhood, breastfeeding was associated with a lower risk of respiratory infections. The highest risk was found in children who were not breastfed (aIRR 2.53 [95% CI 1.51–4.24]). In comparison with breastfeeding 4 to 5 months, exclusively breastfeeding 6 months or more was not associated with a lower risk of enteric or respiratory infections.

Conclusions: Exclusive breastfeeding until 4 months was associated with a lower risk of overall infections in early childhood and a lower risk of hospitalisations for respiratory infections in later childhood.

For peer review only - http://bmjopen.bmj.com/site/about/guidelines.xhtml

Strengths and limitations of this study:

This register-based cohort study examines the associations between breastfeeding practices and hospitalisations for infectious diseases in early and later childhood.

The study includes 37 825 full-term singletons born in one county over 13 years.

Data from high-quality health and administrative registers are used, which allows analyses to be adjusted for key maternal and child characteristics.

Information on breastfeeding status was not continuously collected, information on breastfeeding status was only available at 1 week, 2, 4 and 6 months.

For peer review only

For peer review only - http://bmjopen.bmj.com/site/about/guidelines.xhtml

BACKGROUND

Infectious diseases cause large harm and suffering in childhood. Globally, infectious diseases are a leading cause of death in children under five years of age.[1] In high-income countries, infectious disease remains a major cause of hospitalisations in young children.[2 3] Breast milk contains IgA antibodies and oligosaccharides that prevent microbes to adhere to the mucosa, lactoferrin and lysozyme act directly on the microbes, and growth factors, nucleotides, cytokines stimulate maturation of the infant's immune system.[4 5] Numerous studies have shown that breastfeeding reduces the risk of infectious diseases in infancy.[4 6 7] A few studies have also reported that breastfeeding reduces the risk of infectious diseases after infancy.[8-10]

Although it is well known that breastfeeding protects young children in high-income countries against infections, the optimal breastfeeding practices is still under debate. The World Health Organization recommends exclusive breastfeeding until 6 months of age followed by breastfeeding along with complementary feeding.[11] The American Academy of Pediatrics recommends breastfeeding for at least 12 months with exclusive breastfeeding for about 6 months.[12] In contrast, the European Society for Paediatric Gastroenterology, Hepatology, and Nutrition recommend exclusive breastfeeding for at least 4 months with exclusive or predominant breastfeeding for approximately 6 months.[6] Therefore, the aim of this study was to examine the associations between breastfeeding practices i.e. intensity and duration, and hospitalisations for infectious diseases in early (<2 years) and later childhood (2–4 years) in Uppsala County (Sweden).

METHODS

Setting

In Sweden, all children are offered free primary and hospital care, a free preventive child health care program and a free general immunization program; all working parents are entitled to a generous parental leave scheme.[13] During the study period, Swedish guidelines for breastfeeding duration were consistent with the World Health Organization recommendations. Until 2002, exclusive

For peer review only - http://bmjopen.bmj.com/site/about/guidelines.xhtml

breastfeeding was recommended until four to six months of age. Thereafter, exclusive breastfeeding was recommended for at least 6 months.[14]

Study population

This is a register-based cohort study of full-term singletons born to women who resided in Uppsala County between 1998 and 2010. A database was created by combining data from nationwide and local health and administrative registers. The Swedish Medical Birth Register includes information on prenatal, delivery and neonatal care, with coverage of 98–99 percent of all births.[15] The National Patient Register contains information on all hospitalisations including International Classification of Disease, Tenth Revision (ICD-10) codes for primary diagnosis.[16] The Longitudinal integration database for health insurance and labour market studies, includes information maternal education.[17] The Cause of Death Register provides information on mortality. The Multi-Generation Register contains information about migration and maternal country of birth. The Child Health Care Quality database in Uppsala contains data on breastfeeding, which was collected by child health nurses during free routine health checkups.[13] These registers were linked using the unique personal identification number assigned to every Swedish resident, by the Centre for Epidemiology at the Swedish National Board of Health and Welfare. All data were anonymized and de-identified prior to analysis.

Main Exposures

Breastfeeding was categorised according to intensity and duration. Information on current breastfeeding status (exclusive breastfeeding, partial breastfeeding or no breastfeeding) was collected at 1 week, 2, 4 and 6 months. Breastfeeding was categorised as no breastfeeding if “no breastfeeding” was reported at 1 week. Thereafter, breastfeeding was considered to be present until “no breastfeeding” was reported; exclusive breastfeeding was considered to be present until “partial breastfeeding” or “no breastfeeding” was reported e.g. breastfeeding was categorised as “breastfeeding ≥6 months with exclusive breastfeeding 4–5 months” if “exclusive breastfeeding” was reported at 1 week, 2 months, 4 months and “partial breastfeeding” was reported at 6 months.

For peer review only - http://bmjopen.bmj.com/site/about/guidelines.xhtml

Information on breastfeeding intensity and duration was combined into following categories: No breastfeeding (No BF), breastfeeding <6 months and exclusive breastfeeding <4 months (BF <6 MO and EBF <4 MO), breastfeeding <6 months with exclusive breastfeeding 4–5 months (BF <6 MO with EBF 4–5 MO), breastfeeding ≥6 months and exclusive breastfeeding <4 months (BF ≥6 MO and EBF <4 MO), breastfeeding ≥6 months with exclusive breastfeeding 4–5 months (BF ≥6 MO with EBF 4–5 MO) and exclusive breastfeeding ≥6 months (EBF ≥6 MO). Before 2004, the Swedish definition of exclusive breastfeeding allowed ‘‘small tastes’’ that didn't replace a breast milk feed. In 2004 the WHO definition of exclusive breastfeeding was adopted in Sweden, and tastes were no longer accepted to fulfil this criteria.[18 19]

Covariates

Analyses were adjusted for several child and maternal characteristics. Information on small for gestational age (yes or no), large for gestational age (yes or no), congenital malformation (ICD-10 codes Q00-Q99) and sex (male or female), maternal age (≤19, 20–24, 25–29, 30–34, and ≥35), parity (1, 2, 3, and ≥4), maternal smoking at the first antenatal care visit (yes or no) and year of birth were retrieved from the Medical Birth Register. Maternal education level at year of birth (secondary school or less [≤9 years], upper secondary school [10–12 years], short postsecondary education [13–14 years] and long postsecondary education [≥15 years]) was obtained from the Longitudinal integration database for health insurance and labour market studies. Information on maternal country of birth (Sweden, Other Nordic, Other Europe and North America, Asia, Africa and Other) was retrieved from the Multi-Generation Register.

Outcomes

The main outcome was overall number of hospitalisations with a principal diagnosis of infectious disease recorded in the National Patient Register. Secondary outcomes were number of hospitalisations for respiratory tract and enteric infections. Hospitalisations were recorded using ICD-

For peer review only - http://bmjopen.bmj.com/site/about/guidelines.xhtml

10 codes. We used a previously developed coding scheme to identify infectious disease codes and group them into infectious disease categories (online supplementary appendix A).[3]

Statistical analysis

Crude and adjusted associations between breastfeeding practices and number of hospitalisations were estimated using negative binomial regression models. Log follow-up time (days) was used as an offset. Results were presented as adjusted incidence rate ratios (aIRRs) with 95% confidence intervals (CIs). Correlations between siblings were accounted for using generalized estimating equations with robust standard errors.[20] All analyses were adjusted for time trends (year of birth). The adjusted analyses were also controlled for small for gestational age, large for gestational age, congenital malformation, sex, maternal age, parity, maternal smoking, maternal education level and maternal country of birth. Crude and adjusted models were fitted for each outcome (overall, respiratory and enteric infections) and follow-up period (early childhood [<2 years] and later childhood [2–4 years]). In the later follow-up period, an additional adjusted model controlled for previous admissions (a binary variable indicating hospitalisation in the early follow-up period) was fitted for each outcome. All models were restricted to observations with complete data on breastfeeding and covariates. In sensitivity analyses, the respiratory category was divided into upper and lower respiratory infections, analyses were adjusted for maternal body mass index (BMI) during early pregnancy and analyses were stratified by year of birth (1998 to 2003 and 2004 to 2010). All statistical analyses were performed using Stata V.14 (Stata, 2015. Stata Statistical Software: Release 14. College Station, Texas, USA).

Patient and Public Involvement

This study was conducted without patient involvement.

Ethics

The project was approved by the Regional Ethics Board in Umeå (reference number 2017-399-32M).

For peer review only - http://bmjopen.bmj.com/site/about/guidelines.xhtml

RESULTS

The Swedish Medical Birth Register included 41 825 full-term singletons born to women who resided in Uppsala County between 1998 and 2010. We excluded children with missing data on breastfeeding (n=2127) or covariates (n=1873), leaving 37 825 children (90% of the original cohort). In the first follow-up period (early childhood), all children were followed from birth until 2 years of age or censoring due to death (n=10) or emigration (n=194). In the later follow-up period (later childhood), remaining 37 621 children were followed from 2 until 5 years of age or censoring due to death (n=14) or emigration (n=573). The study included 187 763 person-years (PY) of follow up time, 75 513 PY in early childhood and 112 250 PY in later childhood. In total 4728 hospital admissions for infectious diseases were recorded during the study period. Readmissions on the same day recorded with the same infectious disease group were excluded (n=23) leaving 4705 admissions. In early childhood, the study included 1485 admissions for respiratory infections (799 admissions for upper respiratory infections and 686 admissions for lower respiratory infections), 762 admissions for enteric infections and 1080 admissions for other infections. In later childhood, the study included 630 admissions for respiratory infections (381 admissions for upper respiratory infections and 249 admissions for lower respiratory infections), 300 admissions for enteric infections and 448 admissions for other infections.

Table 1 presents maternal and child characteristics. Most mothers initiated breastfeeding, only 1.6% (n=613) of mothers reported no breastfeeding at one week. A majority of women breastfed their child for 6 months or more with exclusive breastfeeding for at least 4 months, “breastfeeding ≥6 months with exclusive breastfeeding 4–5 months” was reported by 40% and “exclusive breastfeeding ≥6 months” was reported by 25%. Women over 25 years of age and women with a postsecondary education, were more likely to report breastfeeding for 6 months or more with exclusive breastfeeding for at least 4 months. In contrast, women who reported smoking during pregnancy were more likely to report no breastfeeding at one week.

For peer review only - http://bmjopen.bmj.com/site/about/guidelines.xhtml

Figure 1 shows the association between breastfeeding categories and overall hospitalisations for infectious disease in early and later childhood. The risk of hospitalisations for infections decreased with duration of exclusive breastfeeding until 4 months of age. In early childhood, the highest risk was found in children who were not breastfed (aIRR 1.89 [95% CI 1.45–2.47]) and in children breastfed <6 months and exclusive breastfeeding <4 months (aIRR 1.41 [95% CI 1.25–1.59]). Similarly, the risk of hospitalisations for infectious diseases in later childhood was highest in children who were not breastfed (aIRR 1.52 [95% CI 1.00–2.33], controlled for previous admissions) and in children breastfed <6 months and exclusive breastfeeding <4 months (aIRR 1.43 [95% CI 1.16–1.75], controlled for previous admissions). The risk of hospitalisations for infectious diseases was comparable in children exclusively breastfed 4 to 5 months and children exclusively breastfed 6 months or more. Online supplementary appendix B includes full regression results including sensitivity analyses.

Figure 2 shows associations between breastfeeding categories and hospitalisations for respiratory tract and enteric infections in early and later childhood. In early childhood, the risk of both respiratory and enteric infections decreased with duration of exclusive breastfeeding until 4 months of age. The strongest association was found between no breastfeeding and enteric infections (aIRR 3.32 [95% CI 2.14–5.14]). In later childhood, the risk of respiratory infections decreased with duration of exclusive breastfeeding until 4 months of age. The highest risk of respiratory infections was observed in children who were not breastfed (aIRR 2.53 [95% CI 1.51–4.24], controlled for previous admissions). Online supplementary appendix C includes full regression results including sensitivity analyses.

DISCUSSION

We found that the risk of overall hospitalisations for infectious diseases in early childhood (<2 years) and the risk of hospitalisations for respiratory infections in later childhood (2–4 years) decreased with duration of exclusive breastfeeding until 4 months of age. In comparison with exclusive

For peer review only - http://bmjopen.bmj.com/site/about/guidelines.xhtml

breastfeeding 4 to 5 months, exclusive breastfeeding 6 months or more was not associated with a lower risk of infections in early or later childhood.

There is strong evidence that breastfeeding protects infants from infectious diseases.[4 6 7] However, the optimal breastfeeding practices is under discussion. Our results are consistent with a Spanish study reporting that full breastfeeding until at least 4 months would prevent 56.4% (95% CI: 30.9%–69.4%) of non-perinatal infection in the first year of life.[21] Moreover, a Dutch study found that exclusive breastfeeding until 4 months followed by partially breastfeeding decreased the risk of physician-confirmed upper respiratory (adjusted odds ratio [aOR] 0.65 [95% CI: 0.51–0.83]) lower respiratory (aOR 0.50 [95% CI: 0.32–0.79]) and gastrointestinal infections (aOR 0.41 [95% CI: 0.26–0.64]) during the first 6 months. However, the authors concluded that exclusive breastfeeding until 6 months may reduce the risk of infectious diseases even more.[22] In contrast to our findings, a US study reported that full breastfeeding for 6 months was, in comparison with full breastfeeding for 4 months, associated with a decreased risk of respiratory tract infection during the first two years of life.[23] Moreover, observational analysis of the PROBIT trial found that exclusive breastfeeding for 6 months was, compared with exclusive breastfeeding for 3 months and partial breast-feeding thereafter, associated with a decreased risk of gastrointestinal episodes; no significant association was found for gastrointestinal hospitalisations.[7] The Dutch and US studies included less severe infections, whereas our study only included infections requiring hospital care.

There is increasing evidence that breastfeeding also reduces the risk of respiratory infections after infancy. The mechanism is still unknown, however, it has been suggested that breastmilk influence the development of the immune system.[8 24] Our findings are similar to those reported in a recent Japanese study. In that study, exclusive breastfeeding at 6–7 months of age was associated with a reduced risk of hospitalisations for respiratory tract infections (aOR 0.76 [95% CI: 0.58–0.99]) between 30 and 42 months of age, whereas no association was found between breastfeeding and hospitalisations for diarrhea.[9] In a Dutch cohort study, breastfeeding for at least 6 months was

For peer review only - http://bmjopen.bmj.com/site/about/guidelines.xhtml

associated with a decreed risk lower respiratory tract infections (aOR 0.71 [95% CI: 0.51–0.98]) between infancy and 4 years of age.[10] Moreover, a recent US study of children aged 6 years, found that breastfeeding for 9 months or more was associated with a decreased risk of ear (aOR 0.69 [95% CI: 0.48–0.98]), throat (aOR 0.68 [95% CI: 0.47–0.98]), and sinus (aOR 0.47 [95% CI: 0.30–0.72]) infections, but not with cold/upper respiratory tract infections and pneumonia.[8] Overall, these findings are consistent with our results, that breastfeeding reduces the risk of respiratory infections after infancy.

Sensitivity revealed no substantial changes in point estimates after adjusting for pregnancy BMI, indicating no major confounding effect. Moreover, sensitivity showed similar associations between breastfeeding categories and overall hospitalisations for infectious disease in the first (1998–2003) and second (2004–2010) birth cohort. Consequently, the changed definition of exclusive breastfeeding in 2004 did not significantly affect the overall risk of hospitalisation for infectious diseases.

Our study has several strengths. First, the use of high-quality health and administrative register allowed us to adjust analyses for potential cofounders including congenital malformation, large/small for gestational age, maternal age, maternal smoking, maternal education level and maternal country of birth. Secondly, the long follow up period (until 5 years of age or censoring) enabled us to examine the effect of breastfeeding in both early and later childhood. Additionally, this allowed analyses in later childhood to be adjusted for hospitalisations in early childhood. Finally, our study included almost all children (<10% missing data) in one county, thereby reducing the risk of selection bias. However, our study has several weaknesses. In early childhood, infections episodes may influence breastfeeding practices leading to reverse causation. In later childhood, confidence intervals were wide due to the small number of events. Therefore, the associations between breastfeeding and infections in later childhood need to be interpreted with caution. Information on current breastfeeding status was not continuously collected. Compared to studies with complete information

For peer review only - http://bmjopen.bmj.com/site/about/guidelines.xhtml

on breastfeeding practices since birth e.g. collected through daily recordings, our study is likely to overestimate the proportion of exclusively breastfed children.[25] Moreover, Sweden has family-friendly policies that enable breastfeeding including a generous parental leave scheme.[13] Consequently, our findings may not be generalised to other populations with less family-friendly policies and lower breastfeeding rates. Finally, due to the observational nature of this study, we cannot rule out the risk of unmeasured or residual confounding.

CONCLUSIONS

Our study found that the risk of overall hospitalisations for infectious diseases in early childhood and the risk of hospitalisations for respiratory infections in later childhood decreased with duration of exclusive breastfeeding until 4 months of age. Additionally, the risk of hospitalisations for infectious diseases was comparable in children exclusively breastfed 4 to 5 months and children exclusively breastfed 6 months or more. Thereby, it supports the current European Society for Paediatric Gastroenterology, Hepatology, and Nutrition guidelines, recommending exclusive breastfeeding for at least 4 months with exclusive or predominant breastfeeding for approximately 6 months. Moreover, it adds to the growing body of evidence suggesting a protective effect of breastfeeding on respiratory infections after infancy.

For peer review only

Contributors: SV conceptualised and designed the study together with SA, carried out analyses and drafted the initial manuscript. TW supervised the collection of breastfeeding data, contributed to the study design, reviewed and revised the manuscript. SA conceptualised and designed the study together with SV, coordinated and supervised the database, reviewed and revised the manuscript.

Funding: This study was supported by the Unit of Research, Development, and Education, Östersund Hospital, Östersund, Sweden (JLL-930202 [to SV]); ALF Umeå University, Umeå, Sweden (RV-933162 [to SAS]).

Competing interests: We have no conflict of interest to declare.

Patient and Public Involvement: This study was conducted without patient involvement.

Patient consent: Patient informed consent was not needed because the data were collected retrospective and anonymized prior to analysis.

Ethics approval: The project was approved by the Regional Ethics Board in Umeå (reference number 2017-399-32M).

Acknowledgement: The authors would like to thank Anna Lindam, PhD at the Unit of Research, Development, and Education, Östersund Hospital, Sweden, for assistance with statistical analysis and Håkan Sjöberg, MEc at Statistics Sweden, Örebro, Sweden, for help in compiling the dataset.

Data availability statement: We used deidentified register data obtained from third parties. It includes sensitive information and some access restrictions may apply. Interested researchers need to obtain data directly from the National Board of Health and Welfare in Sweden (socialstyrelsen@socialstyrelsen.se), from Statistics Sweden (scb@scb.se) and from the Child Health Service Unit in Uppsala (thomas.wallby@kbh.uu.se).

Children included in the study were identified in the Medical Birth Register, data on hospitalisations were obtained from the Swedish National Patient Register and data on deaths were obtained from the Cause of Death Register. All of these registers are maintained by the National Board of Health

For peer review only - http://bmjopen.bmj.com/site/about/guidelines.xhtml

For peer review only

and Welfare in Sweden. Data on maternal education were obtained from the Longitudinal Integration Database for Health Insurance and Labour market Studies and data on migration were obtained from Multi-Generation Register, both registers are maintained by Statistics Sweden. Data on breastfeeding was obtained from The Child Health Care Quality database, which is maintained by the Child Health Service Unit in Uppsala.

For peer review only

REFERENCES

1 Collaborators GBDCoD. Global, regional, and national age-sex-specific mortality for 282 causes of death in 195 countries and territories, 1980-2017: a systematic analysis for the Global Burden of Disease Study 2017. *Lancet* 2018;392:1736-88.
2 Yorita KL, Holman RC, Sejvar JJ, Steiner CA, Schonberger LB. Infectious disease hospitalizations among infants in the United States. *Pediatrics* 2008;121:244-52.
3 Baker MG, Barnard LT, Kvalsvig A*, et al.* Increasing incidence of serious infectious diseases and inequalities in New Zealand: a national epidemiological study. *Lancet* 2012;379:1112-9.
4 Duijts L, Ramadhani MK, Moll HA. Breastfeeding protects against infectious diseases during infancy in industrialized countries. A systematic review. *Maternal & child nutrition* 2009;5:199-210.
5 Horta BL, Victora, C. G. . Short-term eff ects of breastfeeding: a systematic review of the benefi ts of breastfeeding on diarhoea and pneumonia mortality. Geneva: World Health Organization. 2013.
6 Fewtrell M, Bronsky J, Campoy C*, et al.* Complementary Feeding: A Position Paper by the European Society for Paediatric Gastroenterology, Hepatology, and Nutrition (ESPGHAN) Committee on Nutrition. *J Pediatr Gastroenterol Nutr* 2017;64:119-32.
7 Kramer MS, Kakuma R. Optimal duration of exclusive breastfeeding. *Cochrane Database Syst Rev* 2002:CD003517.
8 Li R, Dee D, Li CM, Hoffman HJ, Grummer-Strawn LM. Breastfeeding and risk of infections at 6 years. *Pediatrics* 2014;134 Suppl 1:S13-20.
9 Yamakawa M, Yorifuji T, Kato T*, et al.* Long-Term Effects of Breastfeeding on Children's Hospitalization for Respiratory Tract Infections and Diarrhea in Early Childhood in Japan. *Matern Child Health J* 2015;19:1956-65.
10 Tromp I, Kiefte-de Jong J, Raat H*, et al.* Breastfeeding and the risk of respiratory tract infections after infancy: The Generation R Study. *PloS one* 2017;12:e0172763.
11 World Health Organization. The Optimal Duration of Exclusive Breastfeeding: Report of an Expert Consultation. 2001.
12 Pediatrics AAo. Breastfeeding and the use of human milk. *Pediatrics* 2012;129.
13 Wettergren B, Blennow M, Hjern A, Soder O, Ludvigsson JF. Child Health Systems in Sweden. *The Journal of pediatrics* 2016;177S:S187-S202.
14 The National Board of Health and Welfare in Sweden. Statistics on Breastfeeding 2014. Available at: https://www.socialstyrelsen.se/globalassets/sharepoint-dokument/artikelkatalog/statistik/2016-9-20.pdf (accessed September 7, 2020).
15 The National Board of Health and Welfare in Sweden. The Swedish Medical Birth Register - A summary of content and quality; 2003.
16 Ludvigsson JF, Andersson E, Ekbom A*, et al.* External review and validation of the Swedish national inpatient register. *BMC public health* 2011;11:450.
17 Ludvigsson JF, Svedberg P, Olen O, Bruze G, Neovius M. The longitudinal integrated database for health insurance and labour market studies (LISA) and its use in medical research. *European journal of epidemiology* 2019;34:423-37.
18 . The National Board of Health and Welfare in Sweden. Kvalitetsdeklaration Statistik om amning 2016. Available at: https://www.socialstyrelsen.se/globalassets/sharepoint-dokument/artikelkatalog/statistik/2018-9-2-kvalitetsdeklaration.pdf (accessed September 7, 2020).
19 . The National Board of Health and Welfare in Sweden. Statistics on breastfeeding 2017. Available at: https://www.socialstyrelsen.se/globalassets/sharepoint-dokument/artikelkatalog/statistik/2019-9-6379.pdf (accessed September 7, 2020).
20 Hanley JA, Negassa A, Edwardes MD, Forrester JE. Statistical analysis of correlated data using generalized estimating equations: an orientation. *American journal of epidemiology* 2003;157:364-75.
21 Paricio Talayero JM, Lizan-Garcia M, Otero Puime A*, et al.* Full breastfeeding and hospitalization as a result of infections in the first year of life. *Pediatrics* 2006;118:e92-9.

For peer review only

22 Duijts L, Jaddoe VW, Hofman A, Moll HA. Prolonged and exclusive breastfeeding reduces the risk of infectious diseases in infancy. *Pediatrics* 2010;126:e18-25.
23 Chantry CJ, Howard CR, Auinger P. Full breastfeeding duration and associated decrease in respiratory tract infection in US children. *Pediatrics* 2006;117:425-32.
24 Hanson LA, Silfverdal SA. The mother's immune system is a balanced threat to the foetus, turning to protection of the neonate. *Acta paediatrica* 2009;98:221-8.
25 Aarts C, Kylberg E, Hornell A, Hofvander Y, Gebre-Medhin M, Greiner T. How exclusive is exclusive breastfeeding? A comparison of data since birth with current status data. *International journal of epidemiology* 2000;29:1041-6.

For peer review only

Table 1. Child and maternal characteristics and their association with breastfeeding categories

	Not BF	BF <6 MO and EBF <4 MO	BF <6 MO with EBF 4–5 MO	BF ≥6 MO and EBF <4 MO	BF ≥6 MO with EBF 4–5 MO	EBF ≥6 MO	All included	Excluded
	% (n)	% (n)	% (n)	% (n)	% (n)	% (n)	n	n
All	1.6 (613)	19 (7335)	3.9 (1488)	11 (3998)	40 (15007)	25 (9384)	37825	4000
SGA								
Yes	1.6 (605)	19 (7191)	3.9 (1466)	11 (3912)	40 (14837)	25 (9278)	37289	3816
No	1.5 (8)	27 (144)	4.1 (22)	16 (86)	32 (170)	20 (106)	536	85
LGA								
Yes	1.6 (570)	19 (6950)	4.0 (1426)	11 (3809)	40 (14393)	25 (8927)	36075	3729
No	2.5 (43)	22 (385)	3.5 (62)	11 (189)	35 (614)	26 (457)	1750	172
Congenital malformation*								
Yes	4.0 (32)	25 (196)	3.6 (29)	9.7 (77)	35 (277)	23 (186)	797	3891
No	1.6 (581)	19 (7139)	3.9 (1459)	11 (3921)	40 (14730)	25 (9198)	37028	109
Sex								
Male	1.6 (319)	20 (3926)	4.1 (795)	11 (2077)	40 (7751)	24 (4731)	19599	2088
Female	1.6 (294)	19 (3409)	3.8 (693)	11 (1921)	40 (7256)	26 (4653)	18226	1912
Maternal age								
≤19	2.5 (10)	47 (187)	8.2 (33)	7.5 (30)	24 (98)	11 (43)	401	146
20-–24	1.6 (67)	33 (1402)	6.7 (282)	9.0 (378)	32 (1362)	17 (723)	4214	607
25–29	1.7 (198)	21 (2366)	4.7 (545)	9.8 (1136)	40 (4582)	24 (2727)	11554	1167
30–34	1.6 (222)	15 (2108)	3.4 (470)	11 (1482)	43 (5876)	27 (3682)	13840	1305
≥35	1.5 (116)	16 (1272)	2.0 (158)	12 (972)	40 (3089)	28 (2209)	7816	775
Parity								
1	1.1 (185)	22 (3510)	3.9 (642)	12 (1878)	39 (6384)	23 (3724)	16323	1821
2	1.9 (268)	18 (2486)	4.3 (603)	9.3 (1301)	41 (5738)	26 (3584)	13980	1336
3	2.1 (110)	17 (902)	3.1 (165)	10 (541)	41 (2177)	27 (1440)	5335	535
≥4	2.3 (50)	20 (437)	3.6 (78)	13 (278)	32 (708)	29 (636)	2187	308

For peer review only

For peer review only - http://bmjopen.bmj.com/site/about/guidelines.xhtml

Maternal smoking								
No smoking	1.5 (521)	18 (6136)	3.8 (1337)	11 (3744)	41 (14362)	26 (9061)	35161	2366
Smoking	3.5 (92)	45 (1199)	5.7 (151)	9.5 (254)	24 (645)	12 (323)	2664	255
Maternal education†								
≤9	2.9 (102)	36 (1273)	5.4 (193)	11 (406)	29 (1014)	16 (561)	3549	419
10–12	2.2 (338)	25 (3942)	5.4 (838)	10 (1590)	35 (5516)	22 (3378)	15602	1217
13–14	1.1 (56)	15 (725)	3.2 (161)	10 (500)	42 (2071)	30 (1472)	4985	491
≥14	0.9 (117)	10 (1395)	2.2 (296)	11 (1502)	47 (6406)	29 (3973)	13689	1217
Maternal country of birth								
Sweden	1.6 (526)	19 (6231)	4.1 (1313)	9.9 (3168)	40 (12853)	25 (7977)	32068	2693
Other Nordic	0.8 (6)	17 (134)	2.4 (19)	8.8 (70)	41 (326)	30 (238)	793	128
Other Europe and North America	1.8 (27)	20 (303)	3.2 (48)	12 (185)	37 (564)	25 (382)	1509	339
Asia	1.3 (33)	22 (538)	3.0 (75)	16 (399)	36 (895)	21 (528)	2468	540
Africa	1.9 (12)	11 (67)	3.1 (19)	20 (126)	38 (233)	26 (163)	620	224
Other	2.5 (9)	17 (62)	3.8 (14)	14 (50)	37 (136)	26 (96)	367	73

Children with missing data on breastfeeding (n=2127) or covariates (n=1873) were excluded leaving 37825 children.

*ICD-10 codes: Q00-Q99.

†Maternal education level at year of birth, categorised as 9 years or less, 10–12 years, 13–14 years or 15 years or more.

BF, breastfeeding. EBF, exclusive breastfeeding. MO, Months. SGA, small for gestation age. LGA, large for gestation age.

For peer review only - http://bmjopen.bmj.com/site/about/guidelines.xhtml

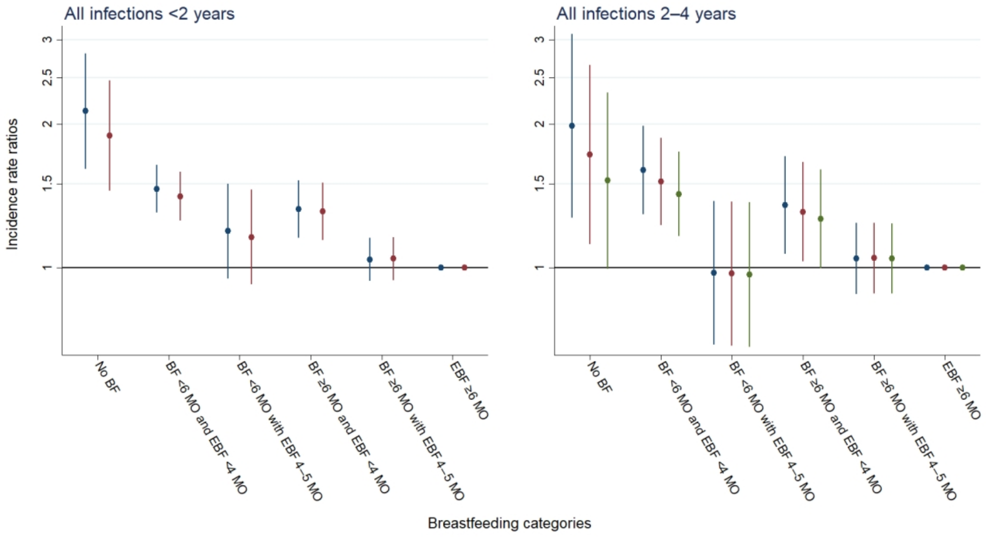

Crude analyses (blue), adjusted analyses (red) and adjusted analyses controlled for previous admissions (green) of the associations between breastfeeding categories and overall hospitalisations for infectious disease, in early childhood (<2 years) and later childhood (2–4 years). Incidence rate ratios are presented on a logarithmic scale. Vertical lines represent 95% confidence intervals around the point estimates. BF, breastfeeding. EBF exclusive breastfeeding. MO, months.

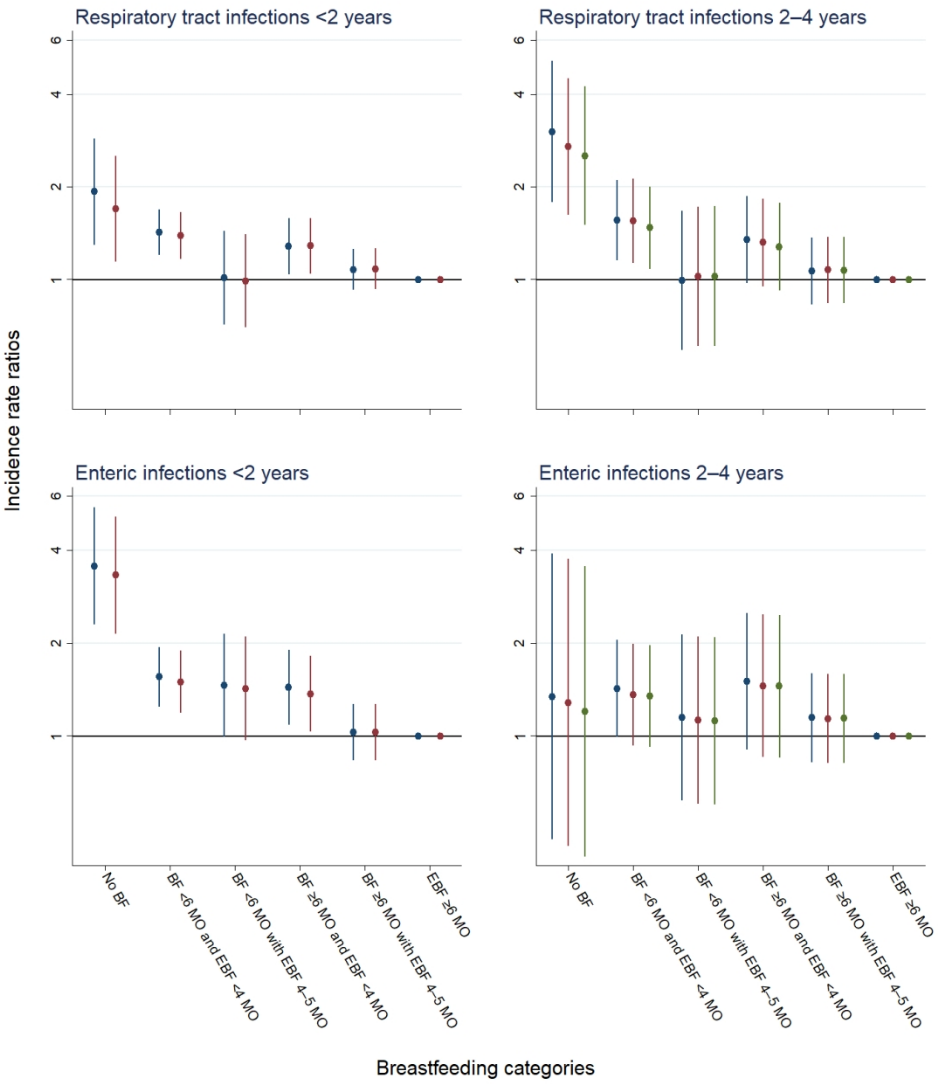

Crude analyses (blue), adjusted analyses (red) and adjusted analyses controlled for previous admissions (green) of the associations between breastfeeding categories, and hospitalisations for respiratory tract and enteric infections, in early childhood (<2 years) and later childhood (2–4 years). Incidence rate ratios are presented on a logarithmic scale. Vertical lines represent 95% confidence intervals around the point estimates. BF, breastfeeding. EBF exclusive breastfeeding. MO, months.

Appendix A

Table A: Infectious disease categories and associated ICD-10 codes

Category	Group	ICD codes
Enteric infections	Enteric infections	A00, A01, A020, A029, A03, A04, A05, A06, A07,A08
	Enteric symptoms	A09, I880, K528, K529, R11
Upper respiratory tract infections	Upper RTI	J00, J01, J02, J03, J04, J05, J06, J32, J340, J36, J37, J390, J391
	Ear infections	H600, H601, H602, H603, H608, H609, H62, H65, H66, H67, H680, H70, H730, H750, H830, H940
Lower respiratory tract infections	Acute lower RTI	A481, A482, B59, J09, J10, J11, J12, J13, J14, J15, J16, J17, J18, J20, J21, J22
	Chronic lower RTI	J40, J41, J42, J440, J47, J85, J86, J988
	Tuberculosis	A15, A16, A17, A18, A19, N740, N741, J65
Other infections	Sepsis	A391, A392, A393, A394, A395, A398, A399 A40, A41, A483, A021, A327
	HIV/AIDS	B20, B21. B22, B23, B24
	STI	A50, A51, A52, A53, A54, A55, A56, A57, A58, A59, A60, A63, A64, N290
	CNS viral infections	A801, A802, A803, A804, A809, A811, A812, A818, A819, A82, A83, A84, A85, A86, A87, A88, A89
	CNS general infections	G00, G01, G02, G030, G039, G04, G05, G06, G07, G08, G09, G610, A321, A390
	Eye infections	B30, H000, H03, H043, H050, H100, H102, H103, H109, H130, H131, H160, H190, H191, H192, H220, H440, H451
	Oral infections	K02, K044, K046, K050, K052, K053, K113, K122
	Heart and Circulatory infections	B332, I00, I01, I02, I05, I06, I07, I08, I09, I301, I33, I38, I39, I400, I410, I411, I412, I430, I716, I790, I791
	Gastrointestinal tract infections	K230, K231, K25, K26, K27, K28, K293, K294, K295, K35, K36, K37, K61, K630, K632, K650, K678, K908, K930
	Hepatic infections	K750, K770, K830
	Viral Hepatitis	B15, B16, B17, B18, B19

For peer review only

For peer review only - http://bmjopen.bmj.com/site/about/guidelines.xhtml

Kidney infections	N00, N05, N10, N136, N151
Urinary tract infections	N300, N341, N351, N37, N390
Reproductive system infections, male	N410, N411, N412, N413, N431, N45, N410, N411, N412, N413, N431, N45, N481, N482, N490, N49, N51
Reproductive system infections, female	N70, N71, N72, N73, N74, N751, N764, N87
Skin infections, typical	A46, L00, L01, L02, L03, L04, L050, L08
Skin infections, other	B86, T009, T633, T634, T793
Breast infections	N61
Osteomyelitis	M462, M463, M464, M465
Joint infections	M00, M01
Connective tissue infections	M021, M023, M03, M600, M630, M631, M632, M650, M651, M680, M710, M711, M896
Neoplasms from infection	C11, C161, C162, C163, C164, C165, C166, C168, C169, C210, C211, C220, C46, C53, D002, D013, D06
Postoperative infections	T802, T814, T826, T827, T835, T836, T845, T846, T847, T857, T874
Adverse effect of ID treatment	R761, R762, T36, T37, T485, T487, T490, T495, T496, T499, T788, T789, T880, T881, T887
Other Bacterial infections	A20, A21, A22, A23, A24, A25, A26, A27, A28, A30, A31, A320, A328, A329, A33, A34, A35, A36, A37, A38, A42, A43, A44, A480, A484, A488, A49, A65, A66, A67, A68, A69, A70, A71, A74, A75, A77, A78, A79, B95, B96
Other Viral infections	A90, A91, A92, A93, A94, A95, A96, A98, A99, B00, B01, B02, B03, B04, B05, B06, B07, A08, B09, B25, B26, B27, B33, B34, B97
Other Mycoses	B35, B36, B37, B38, B39, B40, B41, B42, B43, B44, B45, B46, B47, B48, B49
Other Protozoan infections	B50, B51, B52, B53, B54, B55, B56, B57, B58, B60, B64
Other ID	B65, B66, B67, B68, B69, B70, B71, B72, B73, B74, B75, B76, B77, B78, B79, B80, B81, B82, B83, B85, B86, B87, B88, B89, B94, B99, E033, E321, F024, F071, I88, T64
Perinatal infections	P002, P027, P23, P35, P36, P37, P38, P39

ICD-10, International Classification of Disease, Tenth Revision. RTI, respiratory tract infections. CNS, central nervous system. ID, infectious diseases.

For peer review only

Appendix B

Table B: Regression results for all infections, early childhood (<2 years)

	Crude[*](95% CI)		Adjusted[†](95% CI)		1998-2003[‡](95% CI)		2004-2010[§](95% CI)		BMI[¶](95% CI)	
Breastfeeding:										
No BF	2.13	(1.61–2.81)	1.89	(1.45–2.47)	2.25	(1.51–3.35)	1.64	(1.17–2.32)	1.81	(1.35–2.42)
BF <6 MO and EBF <4 MO	1.46	(1.30–1.64)	1.41	(1.25–1.59)	1.34	(1.13–1.59)	1.48	(1.25–1.74)	1.40	(1.23–1.59)
BF <6 MO with EBF 4–5 MO	1.19	(0.95–1.50)	1.16	(0.92–1.46)	1.21	(0.86–1.71)	1.11	(0.84–1.47)	1.09	(0.84–1.40)
BF ≥6 MO and EBF <4 MO	1.33	(1.16–1.52)	1.31	(1.14–1.50)	1.26	(1.02–1.56)	1.35	(1.12–1.63)	1.29	(1.11–1.49)
BF ≥6 MO with EBF 4–5 MO	1.04	(0.94–1.15)	1.05	(0.94–1.16)	1.06	(0.92–1.23)	1.04	(0.89–1.20)	1.04	(0.93–1.16)
EBF ≥6 MO	1	ref	1	ref	1	ref	1	ref	1	ref
SGA:										
Yes			1.58	(1.14–2.19)	1.59	(0.98–2.57)	1.55	(0.99–2.43)	1.60	(1.12–2.28)
No			1	ref	1	ref	1	ref	1	ref
LGA:										
Yes			1.22	(1.02–1.47)	1.40	(1.06–1.84)	1.08	(0.86–1.36)	1.21	(0.99–1.47)
No			1	ref	1	ref	1	ref	1	ref
Congenital malformation:										
Yes			2.25	(1.82–2.79)	2.18	(1.64–2.90)	2.32	(1.71–3.17)	2.27	(1.82–2.84)
No			1	ref	1	ref	1	ref	1	ref
Sex:										
Male			1	ref	1	ref	1	ref	1	ref
Female			0.89	(0.82–0.96)	0.95	(0.85–1.07)	0.83	(0.75–0.93)	0.88	(0.81–0.96)
Maternal age (years):										
≤19			1.00	(0.71–1.42)	1.03	(0.65–1.63)	0	(0.59–1.63)	1.08	(0.75–1.55)
20-24			1	ref	1	ref	1	ref	1	ref
25-29			1.00	(0.86–1.15)	1.06	(0.85–1.32)	0.96	(0.79–1.17)	1.01	(0.86–1.18)
30-34			0.95	(0.82–1.10)	1.01	(0.80–1.28)	0.91	(0.75–1.11)	0.95	(0.81–1.11)
≥35			0.88	(0.74–1.05)	1.01	(0.77–1.32)	0.80	(0.64–1.00)	0.89	(0.74–1.07)
Parity:										
1			1	ref	1	ref	1	ref	1	ref
2			1.18	(1.07–1.29)	1.06	(0.93–1.22)	1.29	(1.14–1.46)	1.17	(1.06–1.30)
3			1.17	(1.03–1.33)	1.04	(0.86–1.25)	1.30	(1.10–1.55)	1.17	(1.03–1.34)
≥4			1.42	(1.18–1.71)	1.23	(0.95–1.61)	1.62	(1.25–2.10)	1.47	(1.21–1.79)

For peer review only

Maternal smoking:										
No smoking			1	ref	1	ref	1	ref	1	ref
Smoking			1.04	(0.89–1.21)	0.99	(0.80–1.23)	1.10	(0.89–1.37)	1.04	(0.89–1.22)
Maternal education (years):										
≤9			1.07	(0.91–1.25)	1.09	(0.87–1.37)	1.03	(0.83–1.29)	1.05	(0.89–1.24)
10-12			1.03	(0.93–1.13)	1.01	(0.86–1.17)	1.04	(0.91–1.18)	1.04	(0.93–1.16)
13-14			1.01	(0.88–1.15)	1.00	(0.83–1.21)	1.00	(0.83–1.21)	1.02	(0.88–1.17)
≥15			1	ref	1	ref	1	ref	1	ref
Maternal country of birth:										
Sweden			1	ref	1	ref	1	ref	1	ref
Other Nordic			1.06	(0.82–1.37)	1.09	(0.78–1.54)	1.03	(0.70–1.52)	1.07	(0.82–1.40)
Europe and North America			1.04	(0.86–1.25)	1.15	(0.87–1.51)	0.96	(0.74–1.25)	1.08	(0.88–1.33)
Asia			0.94	(0.79–1.12)	0.88	(0.67–1.15)	0.98	(0.79–1.23)	0.93	(0.77–1.13)
Africa			1.41	(1.08–1.84)	1.35	(0.93–1.94)	1.45	(1.01–2.10)	1.48	(1.12–1.95)
Other			0.92	(0.58–1.45)	1.00	(0.48–2.10)	0.84	(0.49–1.45)	0.84	(0.54–1.30)
Year of birth:										
1998	1	ref	1	ref	1	ref			1	ref
1999	0.80	(0.64–0.99)	0.81	(0.66–1.01)	0.81	(0.66–1.00)			0.79	(0.63–0.99)
2000	0.99	(0.82–1.21)	1.02	(0.84–1.24)	1.01	(0.83–1.22)			1.03	(0.84–1.26)
2001	0.89	(0.74–1.09)	0.93	(0.77–1.13)	0.93	(0.76–1.12)			0.94	(0.76–1.16)
2002	0.87	(0.72–1.06)	0.90	(0.74–1.09)	0.89	(0.73–1.08)			0.92	(0.75–1.12)
2003	0.79	(0.65–0.96)	0.82	(0.68–0.99)	0.81	(0.67–0.98)			0.78	(0.63–0.95)
2004	0.84	(0.70–1.02)	0.89	(0.73–1.07)			1	ref	0.94	(0.77–1.15)
2005	0.84	(0.70–1.02)	0.88	(0.73–1.06)			0.99	(0.83–1.20)	0.90	(0.74–1.10)
2006	0.73	(0.60–0.89)	0.77	(0.64–0.93)			0.87	(0.73–1.05)	0.76	(0.62–0.93)
2007	0.69	(0.57–0.84)	0.73	(0.60–0.88)			0.82	(0.68–0.99)	0.71	(0.58–0.87)
2008	0.58	(0.47–0.71)	0.61	(0.50–0.75)			0.69	(0.57–0.84)	0.61	(0.49–0.75)
2009	0.59	(0.48–0.73)	0.62	(0.51–0.77)			0.70	(0.57–0.87)	0.62	(0.50–0.77)
2010	0.55	(0.44–0.67)	0.59	(0.48–0.72)			0.66	(0.54–0.81)	0.58	(0.47–0.72)
Pregnancy BMI:										
Underweight									1.09	(0.82–1.44)
Normal									1	ref
Overweight									0.98	(0.89–1.09)
Obese									1.12	(0.98–1.28)
Observations (N):	37,825		37,825		15,553		22,272		33,422	

For peer review only

Clusters (Maternal ID):	25,487	25,487	12,445	16,998	23,605

*Adjusted for year of birth. Analysis included 37,825 children.

†Adjusted for year of birth small for gestational age, large for gestational age, congenital malformation, sex, maternal age, parity, maternal smoking, maternal education level and maternal country of birth. Analysis included 37,825 children.

‡Included children born 1998 to 2003. Adjusted for year of birth small for gestational age, large for gestational age, congenital malformation, sex, maternal age, parity, maternal smoking, maternal education level and maternal country of birth. Analysis excluded children born 2004 to 2010, leaving 15,553 children.

§Included children born 2004 to 2010. Adjusted for year of birth small for gestational age, large for gestational age, congenital malformation, sex, maternal age, parity, maternal smoking, maternal education level and maternal country of birth. Analysis excluded children born 1998 to 2003, leaving 22,272 children.

¶Adjusted for year of birth small for gestational age, large for gestational age, congenital malformation, sex, maternal age, parity, maternal smoking, maternal education level, maternal country of birth and pregnancy BMI. Analysis excluded children with missing data on pregnancy BMI, leaving 33,422 children.

Incidence rate ratios were estimated using negative binomial regression models. Correlation between siblings was accounted for using generalized estimating equations with robust standard errors. CI, confidence intervals. BF, breastfeeding. EBF, exclusive breastfeeding. MO, months. SGA, small for gestation age. LGA, large for gestation age. BMI, body mass index.

For peer review only

Table C: Regression results for all infections, later childhood (2–4 years)

	Crude*	(95% CI)	Adjusted†	(95% CI)	Adjusted 2‡	(95% CI)	1998-2003§	(95% CI)	2004-2010¶	(95% CI)	BMI#	(95% CI)
Breastfeeding:												
No BF	1.98	(1.27–3.09)	1.73	(1.12–2.66)	1.52	(1.00–2.33)	2.51	(1.54–4.09)	0.82	(0.37–1.82)	1.54	(0.98–2.40)
BF <6 MO and EBF <4 MO	1.60	(1.29–1.98)	1.52	(1.23–1.87)	1.43	(1.16–1.75)	1.29	(0.97–1.71)	1.54	(1.13–2.08)	1.46	(1.17–1.82)
BF <6 MO with EBF 4–5 MO	0.98	(0.69–1.38)	0.97	(0.69–1.38)	0.97	(0.68–1.37)	0.90	(0.59–1.38)	1.08	(0.61–1.89)	1.01	(0.70–1.47)
BF ≥6 MO and EBF <4 MO	1.35	(1.07–1.71)	1.31	(1.03–1.66)	1.27	(1.00–1.61)	1.39	(1.01–1.91)	1.22	(0.86–1.73)	1.32	(1.03–1.69)
BF ≥6 MO with EBF 4–5 MO	1.05	(0.88–1.24)	1.05	(0.88–1.24)	1.05	(0.88–1.24)	1.07	(0.86–1.34)	1.04	(0.81–1.35)	1.04	(0.86–1.24)
EBF ≥6 MO	1	ref	1	ref	1	ref	1	ref	1	ref	1	ref
SGA:												
Yes			2.23	(1.43–3.46)	2.12	(1.37–3.29)	1.68	(0.88–3.23)	2.49	(1.41–4.40)	2.26	(1.45–3.53)
No			1	ref	1	ref	1	ref	1	ref	1	ref
LGA:												
Yes			1.21	(0.91–1.60)	1.17	(0.89–1.54)	1.03	(0.68–1.53)	1.33	(0.92–1.93)	1.08	(0.80–1.45)
No			1	ref	1	ref	1	ref	1	ref	1	ref
Congenital malformation:												
Yes			3.44	(2.36–5.03)	2.89	(2.03–4.11)	2.37	(1.66–3.39)	3.42	(1.97–5.95)	2.96	(2.04–4.29)
No			1	ref	1	ref	1	ref	1	ref	1	ref
Sex												
Male			1	ref	1	ref	1	ref	1	ref	1	ref
Female			0.90	(0.78–1.04)	0.91	(0.79–1.05)	0.94	(0.78–1.12)	0.87	(0.71–1.07)	0.92	(0.79–1.07)
Maternal age (years):												
≤19			0.64	(0.33–1.24)	0.65	(0.33–1.26)	0.67	(0.27–1.53)	0.67	(0.24–1.88)	0.67	(0.33–1.35)
20–24			1	ref	1	ref	1	ref	1	ref	1	ref
25–29			0.98	(0.78–1.25)	0.98	(0.77–1.23)	0.96	(0.71–1.28)	0.99	(0.70–1.39)	1.00	(0.78–1.27)
30–34			0.82	(0.65–1.04)	0.82	(0.65–1.04)	1.02	(0.75–1.39)	0.66	(0.47–0.93)	0.81	(0.63–1.04)
≥35			0.96	(0.73–1.25)	0.98	(0.75–1.27)	1.22	(0.86–1.74)	0.81	(0.56–1.18)	0.97	(0.73–1.29)
Parity:												
1			1	ref	1	ref	1	ref	1	ref	1	ref
2			0.98	(0.84–1.14)	0.96	(0.83–1.12)	0.90	(0.73–1.12)	1.04	(0.84–1.30)	0.98	(0.83–1.15)
3			0.99	(0.80–1.23)	0.97	(0.78–1.20)	0.85	(0.63–1.15)	1.11	(0.82–1.50)	0.97	(0.78–1.23)
≥4			0.99	(0.67–1.48)	0.94	(0.64–1.36)	0.60	(0.39–0.92)	1.33	(0.81–2.18)	0.96	(0.65–1.43)
Maternal smoking:												
No smoking			1	ref	1	ref	1	ref	1	ref	1	ref

For peer review only

Smoking			1.04	(0.80–1.37)	1.04	(0.80–1.34)	0.92	(0.68–1.25)	1.16	(0.78–1.73)	0.96	(0.73–1.27)
Maternal education (years):												
≤9			1.15	(0.87–1.53)	1.13	(0.86–1.49)	1.24	(0.89–1.73)	0.99	(0.65–1.53)	1.19	(0.89–1.57)
10–12			0.90	(0.76–1.07)	0.90	(0.76–1.07)	0.98	(0.77–1.26)	0.81	(0.64–1.03)	0.91	(0.75–1.09)
13–14			1.11	(0.91–1.37)	1.12	(0.92–1.37)	1.10	(0.82–1.46)	1.13	(0.84–1.51)	1.10	(0.89–1.37)
≥15			1	ref	1	ref	1	ref	1	ref	1	ref
Maternal country of birth:												
Sweden			1	ref	1	ref	1	ref	1	ref	1	ref
Other Nordic			0.91	(0.47–1.78)	0.89	(0.46–1.72)	0.66	(0.33–1.31)	1.20	(0.43–3.37)	0.90	(0.44–1.84)
Europe and North America			0.99	(0.72–1.37)	0.98	(0.71–1.35)	1.09	(0.68–1.72)	0.90	(0.58–1.38)	0.86	(0.60–1.24)
Asia			1.04	(0.77–1.40)	1.05	(0.78–1.40)	0.88	(0.60–1.27)	1.17	(0.77–1.76)	1.12	(0.82–1.51)
Africa			1.23	(0.70–2.18)	1.14	(0.66–1.97)	1.19	(0.67–2.12)	1.12	(0.48–2.61)	1.14	(0.64–2.02)
Other			1.05	(0.54–2.05)	1.03	(0.56–1.89)	1.33	(0.58–3.04)	0.76	(0.32–1.80)	0.90	(0.49–1.66)
Year of birth:												
1998	1	ref	1	ref	1	ref			1	ref	1	ref
1999	1.01	(0.74–1.38)	1.05	(0.74–1.38)	1.10	(0.80–1.50)	1.07	(0.79–1.46)			1.18	(0.85–1.62)
2000	0.99	(0.72–1.36)	1.04	(0.72–1.36)	1.03	(0.76–1.41)	1.02	(0.75–1.39)			1.00	(0.72–1.39)
2001	0.93	(0.67–1.29)	0.99	(0.67–1.29)	0.99	(0.72–1.36)	0.97	(0.71–1.32)			0.91	(0.64–1.30)
2002	0.97	(0.71–1.31)	1.01	(0.71–1.31)	1.03	(0.76–1.39)	1.00	(0.74–1.35)			1.05	(0.76–1.44)
2003	0.94	(0.69–1.29)	0.98	(0.69–1.29)	1.01	(0.74–1.37)	0.97	(0.72–1.33)			1.06	(0.76–1.48)
2004	0.72	(0.52–1.00)	0.77	(0.52–1.00)	0.78	(0.56–1.07)	1	ref			0.88	(0.63–1.23)
2005	0.79	(0.55–1.12)	0.83	(0.55–1.12)	0.83	(0.59–1.17)			1.07	(0.76–1.50)	0.77	(0.54–1.11)
2006	0.56	(0.40–0.80)	0.60	(0.40–0.80)	0.62	(0.44–0.87)			0.80	(0.57–1.12)	0.62	(0.43–0.90)
2007	0.70	(0.50–1.00)	0.74	(0.50–1.00)	0.78	(0.55–1.10)			1.01	(0.72–1.44)	0.79	(0.55–1.14)
2008	0.66	(0.46–0.95)	0.71	(0.46–0.95)	0.76	(0.53–1.09)			1.00	(0.70–1.43)	0.79	(0.54–1.15)
2009	0.68	(0.46–1.02)	0.72	(0.46–1.02)	0.76	(0.52–1.13)			0.98	(0.68–1.42)	0.77	(0.52–1.16)
2010	0.53	(0.38–0.74)	0.58	(0.38–0.74)	0.63	(0.45–0.87)			0.83	(0.59–1.15)	0.65	(0.46–0.91)
Previous admissions:												
Yes					3.39	(2.78–4.12)	2.86	(2.24–3.65)	3.94	(2.94–5.28)	3.46	(2.81–4.26)
No					1	ref	1	ref	1	ref	1	ref
Pregnancy BMI:												
Underweight											0.74	(0.45–1.22)
Normal											1	ref
Overweight											1.01	(0.84–1.20)
Obese											1.03	(0.83–1.29)

For peer review only

Observations (N):	37,621	37,621	37,621	15,466	22,155	33,236
Clusters (Maternal ID):	25,344	25,344	25,344	12,377	16,910	23,471

*Adjusted for year of birth. Analysis included 37,621 children.

†Adjusted for year of birth small for gestational age, large for gestational age, congenital malformation, sex, maternal age, parity, maternal smoking, maternal education level and maternal country of birth. Analysis included 37,621 children.

‡Adjusted for year of birth small for gestational age, large for gestational age, congenital malformation, sex, maternal age, parity, maternal smoking, maternal education level, maternal country of birth and previous admissions. Analysis included 37,621 children.

§Included children born 1998 to 2003. Adjusted for year of birth small for gestational age, large for gestational age, congenital malformation, sex, maternal age, parity, maternal smoking, maternal education level, maternal country of birth and previous admissions. Analysis excluded children born 2004 to 2010, leaving 15,466 children.

¶Included children born 2004 to 2010. Adjusted for year of birth small for gestational age, large for gestational age, congenital malformation, sex, maternal age, parity, maternal smoking, maternal education level, maternal country of birth and previous admissions. Analysis excluded children born 1998 to 2003, leaving 22,155 children.

#Adjusted for year of birth small for gestational age, large for gestational age, congenital malformation, sex, maternal age, parity, maternal smoking, maternal education level, maternal country of birth, previous admissions and pregnancy BMI. Analysis excluded children with missing data on pregnancy BMI, leaving 33,236 children.

Incidence rate ratios were estimated using negative binomial regression models. Correlation between siblings was accounted for using generalized estimating equations with robust standard errors. BF, breastfeeding. EBF exclusive breastfeeding. MO, months. SGA, small for gestation age. LGA, large for gestation age. BMI, body mass index.

For peer review only

Appendix C

Table D: Regression results for respiratory infections, early childhood (<2 years)

	Crude*(95% CI)		Adjusted†(95% CI)		1998-2003‡(95% CI)		2004-2010§(95% CI)		BMI¶(95% CI)	
Breastfeeding :										
No BF	1.93	(1.30–2.87)	1.70	(1.15–2.52)	2.18	(1.21–3.93)	1.37	(0.82–2.29)	1.69	(1.11–2.57)
BF <6 MO and EBF <4 MO	1.43	(1.20–1.69)	1.39	(1.17–1.65)	1.38	(1.07–1.78)	1.40	(1.10–1.79)	1.32	(1.10–1.60)
BF <6 MO with EBF 4–5 MO	1.02	(0.72–1.44)	0.99	(0.70–1.40)	1.04	(0.62–1.75)	0.95	(0.61–1.49)	0.88	(0.59–1.30)
BF ≥6 MO and EBF <4 MO	1.28	(1.04–1.58)	1.29	(1.05–1.59)	1.29	(0.93–1.78)	1.28	(0.98–1.69)	1.21	(0.97–1.51)
BF ≥6 MO with EBF 4–5 MO	1.08	(0.93–1.26)	1.09	(0.93–1.26)	1.09	(0.88–1.36)	1.08	(0.88–1.34)	1.04	(0.89–1.22)
EBF ≥6 MO	1	ref	1	ref	1	ref	1	ref	1	ref
SGA:										
Yes			1.85	(1.15–2.97)	1.78	(0.99–3.21)	1.89	(0.94–3.81)	1.92	(1.16–3.20)
No			1	ref	1	ref	1	ref	1	ref
LGA:										
Yes			1.29	(1.02–1.64)	1.50	(1.06–2.10)	1.14	(0.82–1.58)	1.27	(0.98–1.64)
No			1	ref	1	ref	1	ref	1	ref
Congenital malformation:										
Yes			2.35	(1.81–3.05)	2.63	(1.89–3.65)	1.97	(1.29–3.01)	2.36	(1.80–3.11)
No			1	ref	1	ref	1	ref	1	ref
Sex										
Male			1	ref	1	ref	1	ref	1	ref
Female			0.70	(0.62–0.79)	0.78	(0.65–0.93)	0.64	(0.55–0.75)	0.69	(0.61–0.78)
Maternal age (years):										
≤19			0.99	(0.55–1.79)	0.70	(0.28–1.76)	0	(0.61–2.80)	1.10	(0.60–2.04)
20-24			1	ref	1	ref	1	ref	1	ref
25-29			1.05	(0.85–1.30)	1.10	(0.80–1.53)	1.02	(0.77–1.36)	1.06	(0.84–1.34)
30-34			1.05	(0.84–1.31)	1.03	(0.73–1.45)	1.08	(0.81–1.43)	1.06	(0.84–1.35)
≥35			0.97	(0.76–1.25)	1.11	(0.75–1.65)	0.89	(0.64–1.23)	1.00	(0.76–1.31)
Parity										
1			1	ref	1	ref	1	ref	1	ref
2			1.47	(1.28–1.68)	1.31	(1.06–1.61)	1.61	(1.34–1.93)	1.44	(1.24–1.67)
3			1.37	(1.13–1.66)	1.11	(0.81–1.51)	1.63	(1.28–2.07)	1.36	(1.11–1.66)
≥4			1.63	(1.25–2.13)	1.51	(1.01–2.24)	1.75	(1.22–2.50)	1.64	(1.24–2.17)

Maternal smoking:										
No smoking			1	ref	1	ref	1	ref	1	ref
Smoking			1.17	(0.93–1.46)	1.18	(0.86–1.64)	1.14	(0.83–1.55)	1.15	(0.90–1.47)
Maternal education (years):										
≤9			1.00	(0.79–1.26)	0.99	(0.69–1.41)	1.01	(0.74–1.38)	0.99	(0.77–1.27)
10-12			0.98	(0.85–1.13)	0.98	(0.78–1.22)	0.99	(0.83–1.19)	0.99	(0.85–1.15)
13-14			0.93	(0.77–1.13)	0.93	(0.70–1.23)	0.94	(0.72–1.23)	0.94	(0.77–1.15)
≥15			1	ref	1	ref	1	ref	1	ref
Maternal country of birth:										
Sweden			1	ref	1	ref	1	ref	1	ref
Other Nordic			1.03	(0.72–1.48)	1.04	(0.62–1.74)	1.02	(0.61–1.71)	1.05	(0.72–1.52)
Europe and North America			0.68	(0.49–0.95)	0.72	(0.45–1.15)	0.67	(0.42–1.06)	0.75	(0.53–1.06)
Asia			0.81	(0.62–1.06)	0.73	(0.48–1.12)	0.86	(0.61–1.21)	0.84	(0.63–1.12)
Africa			1.17	(0.78–1.74)	0.87	(0.46–1.66)	1.36	(0.82–2.26)	1.25	(0.82–1.90)
Other			0.85	(0.43–1.66)	0.84	(0.24–2.93)	0.85	(0.41–1.77)	0.65	(0.33–1.28)
Year of birth:										
1998	1	ref	1	ref	1	ref			1	ref
1999	0.82	(0.60–1.13)	0.84	(0.62–1.16)	0.84	(0.61–1.15)			0.84	(0.60–1.17)
2000	0.85	(0.62–1.16)	0.88	(0.65–1.20)	0.87	(0.64–1.18)			0.95	(0.69–1.31)
2001	0.94	(0.72–1.24)	0.99	(0.75–1.30)	0.98	(0.75–1.30)			0.97	(0.72–1.30)
2002	0.87	(0.66–1.15)	0.91	(0.69–1.20)	0.90	(0.68–1.19)			0.90	(0.67–1.22)
2003	0.73	(0.55–0.97)	0.77	(0.58–1.02)	0.76	(0.57–1.01)			0.74	(0.54–1.00)
2004	0.71	(0.54–0.93)	0.75	(0.57–0.98)			1	ref	0.81	(0.61–1.08)
2005	0.91	(0.69–1.19)	0.94	(0.72–1.23)			1.26	(0.97–1.66)	1.01	(0.76–1.33)
2006	0.74	(0.56–0.97)	0.78	(0.59–1.03)			1.04	(0.79–1.38)	0.74	(0.55–1.00)
2007	0.85	(0.65–1.11)	0.90	(0.69–1.17)			1.20	(0.92–1.57)	0.89	(0.67–1.18)
2008	0.58	(0.44–0.77)	0.62	(0.47–0.83)			0.83	(0.63–1.11)	0.60	(0.44–0.81)
2009	0.71	(0.54–0.95)	0.76	(0.57–1.01)			1.02	(0.77–1.36)	0.77	(0.57–1.03)
2010	0.64	(0.48–0.85)	0.70	(0.53–0.92)			0.93	(0.70–1.24)	0.67	(0.50–0.91)
Pregancy BMI:										
Underweight									1.02	(0.67–1.56)
Normal									1	ref
Overweight									0.94	(0.81–1.09)
Obese									1.16	(0.96–1.41)
Observations (N):	37,825		37,825		15,553		22,272		33,422	

Clusters (Maternal ID):	25,487	25,487	12,445	16,998	23,605

*Adjusted for year of birth. Analysis included 37,825 children.

†Adjusted for year of birth small for gestational age, large for gestational age, congenital malformation, sex, maternal age, parity, maternal smoking, maternal education level and maternal country of birth. Analysis included 37,825 children.

‡Included children born 1998 to 2003. Adjusted for year of birth small for gestational age, large for gestational age, congenital malformation, sex, maternal age, parity, maternal smoking, maternal education level and maternal country of birth. Analysis excluded children born 2004 to 2010, leaving 15,553 children.

§Included children born 2004 to 2010. Adjusted for year of birth small for gestational age, large for gestational age, congenital malformation, sex, maternal age, parity, maternal smoking, maternal education level and maternal country of birth. Analysis excluded children born 1998 to 2003, leaving 22,272 children.

¶Adjusted for year of birth small for gestational age, large for gestational age, congenital malformation, sex, maternal age, parity, maternal smoking, maternal education level, maternal country of birth and pregnancy BMI. Analysis excluded children with missing data on pregnancy BMI, leaving 33,422 children.

Incidence rate ratios were estimated using negative binomial regression models. Correlation between siblings was accounted for using generalized estimating equations with robust standard errors. CI, confidence intervals. BF, breastfeeding. EBF, exclusive breastfeeding. MO, months. SGA, small for gestation age. LGA, large for gestation age. BMI, body mass index.

For peer review only

Table E: Regression results for respiratory infections, later childhood (2–4 years)

	Crude*(95% CI)		Adjusted†(95% CI)		Adjusted 2‡(95% CI)		1998-2003§(95% CI)		2004-2010¶(95% CI)		BMI#(95% CI)	
Breastfeeding:												
No BF	3.03	(1.78–5.13)	2.70	(1.62–4.50)	2.53	(1.51–4.24)	4.60	(2.49–8.48)	1.06	(0.41–2.76)	2.60	(1.52–4.45)
BF <6 MO and EBF <4 MO	1.56	(1.15–2.11)	1.55	(1.13–2.12)	1.47	(1.08–2.01)	1.58	(1.04–2.40)	1.37	(0.87–2.15)	1.50	(1.07–2.10)
BF <6 MO with EBF 4–5 MO	0.99	(0.59–1.67)	1.03	(0.61–1.73)	1.03	(0.61–1.73)	0.86	(0.43–1.73)	1.24	(0.59–2.61)	1.11	(0.65–1.91)
BF ≥6 MO and EBF <4 MO	1.35	(0.98–1.87)	1.32	(0.95–1.83)	1.28	(0.92–1.77)	1.44	(0.89–2.33)	1.18	(0.77–1.83)	1.35	(0.96–1.90)
BF ≥6 MO with EBF 4–5 MO	1.07	(0.83–1.37)	1.08	(0.84–1.38)	1.07	(0.84–1.38)	1.10	(0.78–1.56)	1.04	(0.73–1.48)	1.08	(0.83–1.40)
EBF ≥6 MO	1	ref	1	ref	1	ref	1	ref	1	ref	1	ref
SGA												
Yes			2.26	(1.26–4.04)	2.26	(1.25–4.07)	1.45	(0.57–3.68)	3.10	(1.50–6.43)	2.44	(1.32–4.50)
No			1	ref	1	ref	1	ref	1	ref	1	ref
LGA												
Yes			1.17	(0.79–1.74)	1.16	(0.78–1.72)	1.05	(0.57–1.92)	1.24	(0.75–2.05)	1.15	(0.76–1.75)
No			1	ref	1	ref	1	ref	1	ref	1	ref
Congenital malformation:												
Yes			3.75	(2.59–5.43)	3.22	(2.24–4.62)	2.82	(1.71–4.66)	3.44	(2.07–5.73)	3.11	(2.11–4.58)
No			1	ref	1	ref	1	ref	1	ref	1	ref
Sex												
Male			1	ref	1	ref	1	ref	1	ref	1	ref
Female			0.79	(0.65–0.95)	0.82	(0.67–0.99)	0.91	(0.69–1.19)	0.73	(0.56–0.95)	0.84	(0.68–1.02)
Maternal age (years):												
≤19			0.58	(0.21–1.61)	0.61	(0.22–1.69)	0.72	(0.13–2.29)	0.70	(0.16–3.06)	0.72	(0.25–2.04)
20-24			1	ref	1	ref	1	ref	1	ref	1	ref
25-29			1.19	(0.85–1.66)	1.19	(0.85–1.66)	1.17	(0.72–1.88)	1.20	(0.75–1.91)	1.24	(0.87–1.78)
30-34			1.06	(0.76–1.48)	1.06	(0.76–1.48)	1.51	(0.94–2.45)	0.75	(0.47–1.19)	1.14	(0.79–1.63)
≥35			1.39	(0.95–2.03)	1.39	(0.95–2.03)	1.88	(1.10–3.22)	1.05	(0.62–1.77)	1.46	(0.97–2.19)
Parity:												
1			1	ref	1	ref	1	ref	1	ref	1	ref
2			1.04	(0.83–1.29)	1.01	(0.81–1.25)	0.85	(0.62–1.16)	1.22	(0.91–1.64)	1.01	(0.80–1.27)
3			0.88	(0.63–1.23)	0.86	(0.62–1.19)	0.56	(0.35–0.92)	1.25	(0.81–1.93)	0.83	(0.58–1.19)
≥4			0.92	(0.60–1.41)	0.88	(0.58–1.36)	0.62	(0.35–1.12)	1.24	(0.67–2.30)	0.94	(0.61–1.47)
Maternal smoking:												

For peer review only

No smoking			1	ref	1	ref	1	ref	1	ref	1	ref
Smoking			0.91	(0.64–1.31)	0.89	(0.63–1.28)	0.93	(0.61–1.44)	0.84	(0.45–1.54)	0.78	(0.52–1.15)
Maternal education (years):												
≤9			1.12	(0.77–1.63)	1.11	(0.76–1.61)	1.36	(0.85–2.17)	0.90	(0.49–1.66)	1.18	(0.80–1.73)
10-12			0.96	(0.76–1.21)	0.96	(0.76–1.22)	1.15	(0.82–1.64)	0.82	(0.60–1.13)	1.02	(0.79–1.31)
13-14			1.13	(0.84–1.51)	1.14	(0.86–1.52)	1.05	(0.69–1.59)	1.28	(0.87–1.90)	1.20	(0.88–1.62)
≥15			1	ref	1	ref	1	ref	1	ref	1	ref
Maternal country of birth:												
Sweden			1	ref	1	ref	1	ref	1	ref	1	ref
Other Nordic			0.82	(0.44–1.52)	0.80	(0.43–1.48)	0.80	(0.33–1.91)	0.77	(0.32–1.86)	0.78	(0.40–1.50)
Europe and North America			0.58	(0.33–1.03)	0.60	(0.34–1.07)	0.74	(0.32–1.75)	0.50	(0.24–1.07)	0.48	(0.25–0.89)
Asia			1.05	(0.67–1.62)	1.08	(0.70–1.68)	1.05	(0.62–1.78)	1.04	(0.54–1.99)	1.15	(0.75–1.78)
Africa			1.15	(0.46–2.85)	1.10	(0.44–2.77)	1.21	(0.53–2.79)	1.00	(0.23–4.32)	1.10	(0.41–2.98)
Other			1.48	(0.58–3.80)	1.38	(0.59–3.20)	2.25	(0.89–5.65)	0.61	(0.15–2.41)	1.00	(0.42–2.40)
Year of birth:												
1998	1	ref	1	ref	1	ref			1	ref	1	ref
1999	1.02	(0.63–1.64)	1.07	(0.63–1.64)	1.11	(0.69–1.78)	1.10	(0.68–1.76)			1.18	(0.72–1.94)
2000	1.18	(0.74–1.88)	1.25	(0.74–1.88)	1.27	(0.80–2.02)	1.25	(0.79–1.97)			1.32	(0.80–2.15)
2001	1.02	(0.63–1.66)	1.08	(0.63–1.66)	1.07	(0.67–1.71)	1.02	(0.64–1.62)			1.02	(0.61–1.71)
2002	0.85	(0.53–1.36)	0.88	(0.53–1.36)	0.89	(0.56–1.43)	0.87	(0.54–1.40)			0.84	(0.50–1.41)
2003	0.92	(0.57–1.49)	0.96	(0.57–1.49)	0.97	(0.60–1.56)	0.94	(0.58–1.52)			1.04	(0.62–1.74)
2004	0.94	(0.59–1.52)	1.00	(0.59–1.52)	1.04	(0.64–1.67)	1	ref			1.16	(0.69–1.93)
2005	0.76	(0.47–1.23)	0.79	(0.47–1.23)	0.79	(0.49–1.27)			0.76	(0.48–1.21)	0.76	(0.46–1.27)
2006	0.72	(0.43–1.22)	0.77	(0.43–1.22)	0.78	(0.47–1.31)			0.75	(0.47–1.21)	0.80	(0.46–1.40)
2007	0.86	(0.54–1.37)	0.91	(0.54–1.37)	0.92	(0.58–1.46)			0.90	(0.57–1.41)	1.00	(0.62–1.62)
2008	0.57	(0.33–0.96)	0.60	(0.33–0.96)	0.64	(0.38–1.08)			0.62	(0.38–1.03)	0.66	(0.38–1.15)
2009	0.89	(0.55–1.42)	0.96	(0.55–1.42)	0.99	(0.62–1.59)			0.98	(0.61–1.55)	0.97	(0.59–1.61)
2010	0.56	(0.34–0.91)	0.62	(0.34–0.91)	0.64	(0.40–1.05)			0.64	(0.40–1.03)	0.69	(0.41–1.14)
Previous admissions:												
Yes					4.48	(3.28–6.11)	4.68	(3.14–6.98)	4.20	(2.63–6.71)	4.47	(3.20–6.25)
No					1	ref	1	ref	1	ref	1	ref
Pregancy BMI:												
Underweight											0.55	(0.22–1.36)
Normal											1	ref
Overweight											0.85	(0.66–1.10)

For peer review only

Obese						1.05	(0.78–1.43)
Observations (N):	37,621	37,621	37,621	15,466	22,155	33,236	
Clusters (Maternal ID):	25,344	25,344	25,344	12,377	16,910	23,471	

*Adjusted for year of birth. Analysis included 37,621 children.

†Adjusted for year of birth small for gestational age, large for gestational age, congenital malformation, sex, maternal age, parity, maternal smoking, maternal education level and maternal country of birth. Analysis included 37,621 children.

‡Adjusted for year of birth small for gestational age, large for gestational age, congenital malformation, sex, maternal age, parity, maternal smoking, maternal education level, maternal country of birth and previous admissions. Analysis included 37,621 children.

§Included children born 1998 to 2003. Adjusted for year of birth small for gestational age, large for gestational age, congenital malformation, sex, maternal age, parity, maternal smoking, maternal education level, maternal country of birth and previous admissions. Analysis excluded children born 2004 to 2010, leaving 15,466 children.

¶Included children born 2004 to 2010. Adjusted for year of birth small for gestational age, large for gestational age, congenital malformation, sex, maternal age, parity, maternal smoking, maternal education level, maternal country of birth and previous admissions. Analysis excluded children born 1998 to 2003, leaving 22,155 children.

#Adjusted for year of birth small for gestational age, large for gestational age, congenital malformation, sex, maternal age, parity, maternal smoking, maternal education level, maternal country of birth, previous admissions and pregnancy BMI. Analysis excluded children with missing data on pregnancy BMI, leaving 33,236 children.

Incidence rate ratios were estimated using negative binomial regression models. Correlation between siblings was accounted for using generalized estimating equations with robust standard errors. CI, confidence intervals. BF, breastfeeding. EBF, exclusive breastfeeding. MO, months. SGA, small for gestation age. LGA, large for gestation age. BMI, body mass index.

For peer review only

Table F: Regression results for enteric infections, early childhood (<2 years)

	Crude*	(95% CI)	Adjusted†	(95% CI)	1998-2003‡	(95% CI)	2004-2010§	(95% CI)	BMI¶	(95% CI)
Breastfeeding :										
No BF	3.56	(2.30–5.52)	3.32	(2.14–5.14)	3.54	(1.85–6.78)	3.34	(1.85–6.01)	3.17	(1.99–5.05)
BF <6 MO and EBF <4 MO	1.55	(1.24–1.94)	1.50	(1.19–1.89)	1.50	(1.09–2.07)	1.56	(1.11–2.20)	1.59	(1.24–2.05)
BF <6 MO with EBF 4–5 MO	1.46	(0.99–2.15)	1.43	(0.97–2.10)	0.99	(0.53–1.86)	2.01	(1.22–3.32)	1.38	(0.89–2.13)
BF ≥6 MO and EBF <4 MO	1.44	(1.09–1.91)	1.37	(1.03–1.82)	1.26	(0.82–1.94)	1.52	(1.03–2.25)	1.47	(1.09–1.98)
BF ≥6 MO with EBF 4–5 MO	1.03	(0.83–1.27)	1.03	(0.83–1.27)	1.07	(0.81–1.40)	1.03	(0.74–1.43)	1.07	(0.85–1.34)
EBF ≥6 MO	1	ref	1	ref	1	ref	1	ref	1	ref
SGA:										
Yes			1.47	(0.90–2.40)	1.34	(0.63–2.83)	1.58	(0.82–3.03)	1.69	(1.04–2.76)
No			1	ref	1	ref	1	ref	1	ref
LGA:										
Yes			1.01	(0.70–1.44)	1.15	(0.71–1.86)	0.87	(0.51–1.46)	0.91	(0.61–1.35)
No			1	ref	1	ref	1	ref	1	ref
Congenital malformation:										
Yes			2.16	(1.47–3.17)	1.70	(1.02–2.84)	2.87	(1.66–4.95)	2.30	(1.54–3.41)
No			1	ref	1	ref	1	ref	1	ref
Sex										
Male			1	ref	1	ref	1	ref	1	ref
Female			1.05	(0.90–1.23)	0.95	(0.76–1.18)	1.15	(0.92–1.44)	1.02	(0.86–1.20)
Maternal age (years):										
≤19			0.64	(0.29–1.41)	0.76	(0.26–2.18)	0	(0.17–1.75)	0.68	(0.29–1.59)
20-24			1	ref	1	ref	1	ref	1	ref
25-29			1.04	(0.79–1.36)	1.34	(0.93–1.93)	0.83	(0.56–1.22)	1.02	(0.76–1.36)
30-34			0.89	(0.67–1.19)	1.11	(0.74–1.65)	0.75	(0.50–1.12)	0.87	(0.64–1.18)
≥35			0.84	(0.60–1.17)	1.10	(0.69–1.76)	0.68	(0.44–1.08)	0.82	(0.57–1.17)
Parity										
1			1	ref	1	ref	1	ref	1	ref
2			0.89	(0.74–1.07)	0.77	(0.59–1.00)	1.03	(0.79–1.33)	0.91	(0.75–1.11)
3			0.95	(0.73–1.22)	0.91	(0.63–1.29)	0.98	(0.68–1.41)	0.99	(0.75–1.29)
≥4			1.20	(0.81–1.79)	0.80	(0.47–1.35)	1.65	(0.96–2.84)	1.37	(0.91–2.08)
Maternal smoking:										

For peer review only

No smoking			1	ref	1	ref	1	ref	1	ref
Smoking			0.97	(0.71–1.32)	0.60	(0.37–0.97)	1.46	(0.98–2.17)	1.04	(0.76–1.43)
Maternal education (years):										
≤9			1.13	(0.84–1.53)	1.28	(0.84–1.98)	0.99	(0.65–1.50)	1.03	(0.74–1.43)
10-12			0.98	(0.80–1.19)	0.94	(0.71–1.25)	0.96	(0.73–1.27)	0.99	(0.79–1.23)
13-14			0.98	(0.75–1.29)	0.88	(0.61–1.28)	1.02	(0.70–1.51)	0.94	(0.70–1.27)
≥15			1	ref	1	ref	1	ref	1	ref
Maternal country of birth:										
Sweden			1	ref	1	ref	1	ref	1	ref
Other Nordic			1.11	(0.67–1.82)	0.99	(0.49–2.00)	1.24	(0.61–2.52)	1.09	(0.64–1.85)
Europe and North America			1.40	(0.99–1.99)	0.97	(0.56–1.68)	1.82	(1.16–2.85)	1.48	(1.02–2.14)
Asia			1.28	(0.95–1.71)	0.87	(0.54–1.41)	1.63	(1.12–2.37)	1.29	(0.94–1.78)
Africa			2.13	(1.34–3.38)	2.26	(1.22–4.18)	2.10	(1.11–3.96)	2.08	(1.28–3.38)
Other			1.15	(0.58–2.29)	0.90	(0.29–2.77)	1.35	(0.56–3.22)	1.01	(0.45–2.23)
Year of birth:										
1998	1	ref	1	ref	1	ref			1	ref
1999	0.99	(0.67–1.47)	1.01	(0.68–1.48)	1.01	(0.68–1.49)			0.85	(0.56–1.30)
2000	1.30	(0.90–1.86)	1.32	(0.92–1.89)	1.31	(0.91–1.89)			1.27	(0.87–1.84)
2001	0.83	(0.56–1.23)	0.86	(0.57–1.28)	0.85	(0.57–1.27)			0.80	(0.53–1.22)
2002	0.77	(0.52–1.14)	0.78	(0.53–1.16)	0.77	(0.52–1.14)			0.77	(0.51–1.15)
2003	1.13	(0.78–1.62)	1.14	(0.79–1.64)	1.11	(0.77–1.61)			0.89	(0.60–1.33)
2004	1.36	(0.94–1.95)	1.41	(0.98–2.03)			1	ref	1.42	(0.97–2.08)
2005	0.95	(0.65–1.38)	0.98	(0.67–1.44)			0.70	(0.49–1.00)	0.91	(0.61–1.35)
2006	0.68	(0.46–1.02)	0.71	(0.47–1.06)			0.51	(0.35–0.74)	0.67	(0.44–1.01)
2007	0.64	(0.43–0.97)	0.66	(0.44–1.00)			0.47	(0.32–0.70)	0.66	(0.44–1.01)
2008	0.53	(0.35–0.80)	0.54	(0.36–0.83)			0.39	(0.26–0.57)	0.54	(0.35–0.82)
2009	0.59	(0.38–0.90)	0.60	(0.39–0.92)			0.42	(0.28–0.64)	0.57	(0.37–0.89)
2010	0.45	(0.28–0.71)	0.46	(0.29–0.74)			0.33	(0.21–0.51)	0.45	(0.28–0.71)
Pregancy BMI:										
Underweight									0.88	(0.52–1.48)
Normal									1	ref
Overweight									0.85	(0.68–1.05)
Obese									1.10	(0.85–1.42)
Observations (N):	37,825		37,825		15,553		22,272		33,422	
Clusters (Maternal ID):	25,487		25,487		12,445		16,998		23,605	

For peer review only - http://bmjopen.bmj.com/site/about/guidelines.xhtml

*Adjusted for year of birth. Analysis included 37,825 children.

†Adjusted for year of birth small for gestational age, large for gestational age, congenital malformation, sex, maternal age, parity, maternal smoking, maternal education level and maternal country of birth. Analysis included 37,825 children.

‡Included children born 1998 to 2003. Adjusted for year of birth small for gestational age, large for gestational age, congenital malformation, sex, maternal age, parity, maternal smoking, maternal education level and maternal country of birth. Analysis excluded children born 2004 to 2010, leaving 15,553 children.

§Included children born 2004 to 2010. Adjusted for year of birth small for gestational age, large for gestational age, congenital malformation, sex, maternal age, parity, maternal smoking, maternal education level and maternal country of birth. Analysis excluded children born 1998 to 2003, leaving 22,272 children.

¶Adjusted for year of birth small for gestational age, large for gestational age, congenital malformation, sex, maternal age, parity, maternal smoking, maternal education level, maternal country of birth and pregnancy BMI. Analysis excluded children with missing data on pregnancy BMI, leaving 33,422 children.

Incidence rate ratios were estimated using negative binomial regression models. Correlation between siblings was accounted for using generalized estimating equations with robust standard errors. CI, confidence intervals. BF, breastfeeding. EBF, exclusive breastfeeding. MO, months. SGA, small for gestation age. LGA, large for gestation age. BMI, body mass index.

For peer review only

Table G: Regression results for enteric infections, later childhood (2–4 years)

	Crude[*]	(95% CI)	Adjusted[†]	(95% CI)	Adjusted 2[‡]	(95% CI)	1998-2003[§]	(95% CI)	2004-2010[¶]	(95% CI)	BMI[#]	(95% CI)
Breastfeeding:												
No BF	1.34	(0.46–3.91)	1.28	(0.44–3.75)	1.20	(0.41–3.55)	1.91	(0.64–5.73)	0.01	(0.01–0.01)	1.06	(0.30–3.70)
BF <6 MO and EBF <4 MO	1.42	(0.99–2.04)	1.36	(0.93–1.99)	1.35	(0.92–1.97)	1.00	(0.61–1.64)	2.77	(1.34–5.73)	1.46	(0.98–2.18)
BF <6 MO with EBF 4–5 MO	1.15	(0.62–2.13)	1.13	(0.60–2.10)	1.12	(0.60–2.09)	0.98	(0.46–2.09)	1.84	(0.59–5.74)	1.06	(0.53–2.10)
BF ≥6 MO and EBF <4 MO	1.50	(0.90–2.50)	1.45	(0.86–2.47)	1.45	(0.85–2.47)	1.15	(0.65–2.03)	2.82	(1.02–7.84)	1.51	(0.86–2.66)
BF ≥6 MO with EBF 4–5 MO	1.15	(0.82–1.60)	1.14	(0.82–1.59)	1.14	(0.82–1.59)	0.96	(0.64–1.43)	2.08	(1.07–4.04)	1.17	(0.82–1.67)
EBF ≥6 MO	1	ref	1	ref	1	ref	1	ref	1	ref	1	ref
SGA												
Yes			2.73	(1.11–6.70)	2.67	(1.08–6.60)	3.17	(0.94–10.60)	1.93	(0.55–6.79)	2.85	(1.16–6.97)
No			1	ref	1	ref	1	ref	1	ref	1	ref
LGA												
Yes			1.06	(0.61–1.82)	1.06	(0.61–1.82)	0.81	(0.38–1.73)	1.51	(0.69–3.29)	1.03	(0.58–1.81)
No			1	ref	1	ref	1	ref	1	ref	1	ref
Congenital malformation:												
Yes			1.97	(1.04–3.71)	1.93	(1.02–3.65)	2.01	(1.00–4.05)	1.56	(0.36–6.83)	2.06	(1.09–3.90)
No			1	ref	1	ref	1	ref	1	ref	1	ref
Sex												
Male			1	ref	1	ref	1	ref	1	ref	1	ref
Female			0.95	(0.73–1.23)	0.95	(0.73–1.23)	0.85	(0.61–1.17)	1.11	(0.73–1.70)	0.92	(0.70–1.20)
Maternal age (years):												
≤19			0.52	(0.15–1.75)	0.53	(0.16–1.77)	0.57	(0.11–2.20)	0.61	(0.08–4.61)	0.57	(0.17–1.95)
20-24			1	ref	1	ref	1	ref	1	ref	1	ref
25-29			0.73	(0.50–1.07)	0.73	(0.50–1.07)	0.61	(0.38–0.98)	1.01	(0.54–1.89)	0.74	(0.49–1.09)
30-34			0.63	(0.41–0.96)	0.63	(0.41–0.97)	0.56	(0.32–0.96)	0.79	(0.40–1.54)	0.62	(0.39–0.96)
≥35			0.56	(0.35–0.92)	0.57	(0.35–0.93)	0.62	(0.33–1.14)	0.53	(0.24–1.14)	0.58	(0.35–0.96)
Parity:												
1			1	ref	1	ref	1	ref	1	ref	1	ref
2			1.05	(0.76–1.45)	1.05	(0.76–1.45)	1.05	(0.70–1.58)	1.05	(0.63–1.76)	1.05	(0.75–1.47)
3			1.07	(0.71–1.61)	1.07	(0.71–1.61)	1.07	(0.62–1.86)	1.11	(0.61–2.00)	1.05	(0.68–1.60)
≥4			1.04	(0.57–1.91)	1.04	(0.57–1.90)	0.83	(0.38–1.84)	1.42	(0.59–3.46)	0.95	(0.50–1.82)
Maternal smoking:												

For peer review only

No smoking			1	ref	1	ref	1	ref	1	ref	1	ref
Smoking			1.04	(0.67–1.61)	1.05	(0.68–1.63)	0.66	(0.35–1.22)	2.02	(1.12–3.66)	1.07	(0.69–1.68)
Maternal education (years):												
≤9			1.05	(0.64–1.72)	1.04	(0.63–1.70)	1.08	(0.58–2.03)	0.91	(0.42–1.95)	1.00	(0.60–1.68)
10-12			0.71	(0.50–1.00)	0.71	(0.50–1.00)	0.62	(0.40–0.97)	0.85	(0.51–1.42)	0.66	(0.45–0.95)
13-14			0.78	(0.52–1.18)	0.78	(0.52–1.19)	0.72	(0.43–1.21)	0.81	(0.41–1.61)	0.71	(0.45–1.10)
≥15			1	ref	1	ref	1	ref	1	ref	1	ref
Maternal country of birth:												
Sweden			1	ref	1	ref	1	ref	1	ref	1	ref
Other Nordic			2.15	(0.71–6.47)	2.14	(0.71–6.46)	1.02	(0.30–3.46)	4.88	(1.06–22.56)	2.31	(0.76–7.04)
Europe and North America			1.68	(1.00–2.84)	1.66	(0.98–2.80)	1.96	(1.04–3.72)	1.12	(0.46–2.72)	1.56	(0.87–2.79)
Asia			0.95	(0.57–1.57)	0.94	(0.57–1.57)	0.60	(0.26–1.39)	1.43	(0.75–2.74)	1.06	(0.63–1.76)
Africa			1.95	(0.89–4.28)	1.90	(0.87–4.18)	2.08	(0.84–5.19)	1.72	(0.40–7.32)	1.89	(0.80–4.45)
Other			0.74	(0.18–3.00)	0.74	(0.18–3.01)	0.58	(0.08–4.19)	1.02	(0.14–7.40)	0.82	(0.20–3.35)
Year of birth:												
1998	1	ref	1	ref	1	ref			1	ref	1	ref
1999	0.85	(0.50–1.47)	0.85	(0.50–1.47)	0.85	(0.49–1.46)	0.85	(0.49–1.46)			0.88	(0.50–1.53)
2000	0.56	(0.32–0.96)	0.56	(0.32–0.96)	0.55	(0.32–0.95)	0.55	(0.32–0.95)			0.52	(0.29–0.92)
2001	0.60	(0.33–1.09)	0.61	(0.33–1.09)	0.62	(0.34–1.12)	0.61	(0.34–1.10)			0.58	(0.30–1.11)
2002	0.89	(0.54–1.48)	0.90	(0.54–1.48)	0.91	(0.55–1.50)	0.88	(0.53–1.45)			0.99	(0.59–1.64)
2003	0.75	(0.46–1.23)	0.75	(0.46–1.23)	0.74	(0.46–1.21)	0.73	(0.45–1.18)			0.76	(0.46–1.27)
2004	0.36	(0.20–0.64)	0.36	(0.20–0.64)	0.36	(0.20–0.64)	1	ref			0.41	(0.23–0.75)
2005	0.43	(0.24–0.75)	0.43	(0.24–0.75)	0.43	(0.24–0.76)			1.27	(0.66–2.46)	0.43	(0.24–0.77)
2006	0.36	(0.20–0.64)	0.36	(0.20–0.64)	0.37	(0.21–0.65)			1.08	(0.55–2.12)	0.35	(0.19–0.64)
2007	0.28	(0.11–0.73)	0.28	(0.11–0.73)	0.28	(0.11–0.72)			0.84	(0.31–2.30)	0.27	(0.10–0.73)
2008	0.33	(0.18–0.59)	0.32	(0.18–0.59)	0.33	(0.18–0.60)			0.98	(0.49–1.94)	0.32	(0.17–0.59)
2009	0.25	(0.13–0.50)	0.24	(0.13–0.50)	0.25	(0.12–0.50)			0.75	(0.34–1.63)	0.23	(0.11–0.48)
2010	0.27	(0.15–0.50)	0.26	(0.15–0.50)	0.27	(0.15–0.50)			0.83	(0.40–1.70)	0.23	(0.12–0.45)
Previous admissions:												
Yes					3.03	(1.83–5.01)	1.96	(0.94–4.08)	5.18	(2.56–10.49)	2.87	(1.68–4.91)
No					1	ref	1	ref	1	ref	1	ref
Pregancy BMI:												
Underweight											0.76	(0.31–1.87)
Normal											1	ref
Overweight											1.22	(0.87–1.71)

For peer review only

Obese						1.17	(0.78–1.75)
Observations (N):	37,621	37,621	37,621	15,466	22,155	33,236	
Clusters (Maternal ID):	25,344	25,344	25,344	12,377	16,910	23,471	

*Adjusted for year of birth. Analysis included 37,621 children.

†Adjusted for year of birth small for gestational age, large for gestational age, congenital malformation, sex, maternal age, parity, maternal smoking, maternal education level and maternal country of birth. Analysis included 37,621 children.

‡Adjusted for year of birth small for gestational age, large for gestational age, congenital malformation, sex, maternal age, parity, maternal smoking, maternal education level, maternal country of birth and previous admissions. Analysis included 37,621 children.

§Included children born 1998 to 2003. Adjusted for year of birth small for gestational age, large for gestational age, congenital malformation, sex, maternal age, parity, maternal smoking, maternal education level, maternal country of birth and previous admissions. Analysis excluded children born 2004 to 2010, leaving 15,466 children.

¶Included children born 2004 to 2010. Adjusted for year of birth small for gestational age, large for gestational age, congenital malformation, sex, maternal age, parity, maternal smoking, maternal education level, maternal country of birth and previous admissions. Analysis excluded children born 1998 to 2003, leaving 22,155 children.

#Adjusted for year of birth small for gestational age, large for gestational age, congenital malformation, sex, maternal age, parity, maternal smoking, maternal education level, maternal country of birth, previous admissions and pregnancy BMI. Analysis excluded children with missing data on pregnancy BMI, leaving 33,236 children.

Incidence rate ratios were estimated using negative binomial regression models. Correlation between siblings was accounted for using generalized estimating equations with robust standard errors. CI, confidence intervals. BF, breastfeeding. EBF, exclusive breastfeeding. MO, months. SGA, small for gestation age. LGA, large for gestation age. BMI, body mass index.

For peer review only

For peer review only - http://bmjopen.bmj.com/site/about/guidelines.xhtml

Table H: Regression results for upper respiratory infections, early childhood (<2 years)

	Crude*	(95% CI)	Adjusted†	(95% CI)	1998-2003‡	(95% CI)	2004-2010§	(95% CI)	BMI¶	(95% CI)
Breastfeeding :										
No BF	1.48	(0.88–2.49)	1.31	(0.77–2.22)	1.19	(0.56–2.54)	1.54	(0.74–3.21)	1.24	(0.70–2.18)
BF <6 MO and EBF <4 MO	1.48	(1.19–1.84)	1.40	(1.12–1.75)	1.30	(0.97–1.74)	1.60	(1.11–2.30)	1.36	(1.07–1.74)
BF <6 MO with EBF 4–5 MO	0.97	(0.63–1.50)	0.92	(0.60–1.42)	1.01	(0.59–1.73)	0.78	(0.37–1.65)	0.75	(0.46–1.22)
BF ≥6 MO and EBF <4 MO	1.22	(0.92–1.62)	1.21	(0.91–1.61)	1.21	(0.80–1.82)	1.28	(0.84–1.96)	1.15	(0.85–1.56)
BF ≥6 MO with EBF 4–5 MO	1.18	(0.97–1.44)	1.17	(0.96–1.43)	1.07	(0.82–1.38)	1.36	(0.98–1.90)	1.13	(0.91–1.40)
EBF ≥6 MO	1	ref	1	ref	1	ref	1	ref	1	ref
SGA:										
Yes			1.72	(1.06–2.80)	1.97	(1.04–3.73)	1.47	(0.68–3.14)	1.64	(0.95–2.81)
No			1	ref	1	ref	1	ref	1	ref
LGA:										
Yes			1.31	(0.97–1.75)	1.50	(1.03–2.18)	1.09	(0.67–1.76)	1.24	(0.90–1.71)
No			1	ref	1	ref	1	ref	1	ref
Congenital malformation:										
Yes			2.45	(1.78–3.38)	2.33	(1.54–3.52)	2.63	(1.58–4.37)	2.54	(1.81–3.56)
No			1	ref	1	ref	1	ref	1	ref
Sex										
Male			1	ref	1	ref	1	ref	1	ref
Female			0.74	(0.64–0.86)	0.78	(0.63–0.96)	0.70	(0.56–0.87)	0.74	(0.63–0.87)
Maternal age (years):										
≤19			0.72	(0.33–1.58)	0.54	(0.16–1.77)	0	(0.33–2.75)	0.84	(0.38–1.85)
20-24			1	ref	1	ref	1	ref	1	ref
25-29			1.04	(0.80–1.36)	1.01	(0.70–1.44)	1.10	(0.75–1.61)	1.04	(0.79–1.37)
30-34			0.99	(0.75–1.30)	0.86	(0.58–1.26)	1.16	(0.78–1.71)	0.97	(0.72–1.30)
≥35			0.93	(0.68–1.28)	1.02	(0.65–1.58)	0.84	(0.53–1.33)	0.94	(0.67–1.31)
Parity										
1			1	ref	1	ref	1	ref	1	ref
2			1.15	(0.97–1.37)	1.22	(0.96–1.54)	1.09	(0.85–1.39)	1.15	(0.95–1.38)
3			1.01	(0.78–1.32)	0.98	(0.68–1.43)	1.07	(0.74–1.54)	1.03	(0.79–1.36)
≥4			1.08	(0.77–1.51)	1.06	(0.67–1.67)	1.11	(0.68–1.83)	1.02	(0.71–1.45)
Maternal smoking:										

For peer review only

No smoking			1	ref	1	ref	1	ref	1	ref
Smoking			0.95	(0.70–1.29)	1.01	(0.68–1.50)	0.86	(0.53–1.38)	0.95	(0.69–1.31)
Maternal education (years):										
≤9			1.18	(0.87–1.60)	1.03	(0.66–1.61)	1.38	(0.92–2.06)	1.18	(0.85–1.64)
10-12			1.07	(0.89–1.29)	0.98	(0.75–1.27)	1.18	(0.91–1.53)	1.12	(0.91–1.37)
13-14			0.98	(0.76–1.26)	0.92	(0.65–1.29)	1.03	(0.71–1.50)	0.96	(0.73–1.25)
≥15			1	ref	1	ref	1	ref	1	ref
Maternal country of birth:										
Sweden			1	ref	1	ref	1	ref	1	ref
Other Nordic countries			0.94	(0.58–1.52)	0.96	(0.52–1.76)	0.89	(0.40–1.96)	1.07	(0.66–1.73)
Europe and North America			0.82	(0.54–1.23)	0.96	(0.58–1.61)	0.67	(0.34–1.32)	0.89	(0.58–1.36)
Asia			0.78	(0.55–1.10)	0.73	(0.44–1.21)	0.81	(0.51–1.30)	0.86	(0.60–1.23)
Africa			0.91	(0.52–1.61)	0.76	(0.32–1.79)	1.03	(0.49–2.20)	0.98	(0.54–1.77)
Other			0.53	(0.16–1.72)	0.50	(0.07–3.39)	0.55	(0.14–2.22)	0.46	(0.15–1.44)
Year of birth:										
1998	1	ref	1	ref	1	ref			1	ref
1999	0.71	(0.50–1.01)	0.73	(0.51–1.04)	0.72	(0.51–1.03)			0.68	(0.47–0.99)
2000	0.67	(0.46–0.97)	0.69	(0.48–1.00)	0.68	(0.47–0.99)			0.77	(0.53–1.11)
2001	0.78	(0.57–1.07)	0.83	(0.60–1.14)	0.82	(0.59–1.12)			0.80	(0.57–1.12)
2002	0.74	(0.54–1.01)	0.77	(0.56–1.06)	0.75	(0.55–1.04)			0.75	(0.53–1.05)
2003	0.54	(0.38–0.76)	0.57	(0.40–0.80)	0.56	(0.39–0.79)			0.53	(0.37–0.78)
2004	0.47	(0.33–0.67)	0.50	(0.35–0.71)			1	ref	0.54	(0.38–0.78)
2005	0.51	(0.35–0.72)	0.53	(0.37–0.76)			1.07	(0.73–1.57)	0.57	(0.39–0.82)
2006	0.54	(0.38–0.76)	0.57	(0.40–0.81)			1.13	(0.78–1.66)	0.53	(0.37–0.78)
2007	0.45	(0.31–0.63)	0.48	(0.34–0.67)			0.95	(0.65–1.39)	0.50	(0.35–0.71)
2008	0.32	(0.22–0.46)	0.34	(0.23–0.50)			0.68	(0.45–1.03)	0.32	(0.21–0.48)
2009	0.36	(0.25–0.52)	0.38	(0.26–0.56)			0.77	(0.51–1.15)	0.39	(0.27–0.58)
2010	0.29	(0.20–0.43)	0.32	(0.22–0.48)			0.66	(0.43–1.00)	0.31	(0.20–0.46)
Pregancy BMI:										
Underweight									0.80	(0.43–1.47)
Normal									1	ref
Overweight									0.92	(0.75–1.11)
Obese									1.11	(0.86–1.44)
Observations (N):	37,825		37,825		15,553		22,272		33,422	
Clusters (Maternal ID):	25,487		25,487		12,445		16,998		23,605	

For peer review only

*Adjusted for year of birth. Analysis included 37,825 children.

†Adjusted for year of birth small for gestational age, large for gestational age, congenital malformation, sex, maternal age, parity, maternal smoking, maternal education level and maternal country of birth. Analysis included 37,825 children.

‡Included children born 1998 to 2003. Adjusted for year of birth small for gestational age, large for gestational age, congenital malformation, sex, maternal age, parity, maternal smoking, maternal education level and maternal country of birth. Analysis excluded children born 2004 to 2010, leaving 15,553 children.

§Included children born 2004 to 2010. Adjusted for year of birth small for gestational age, large for gestational age, congenital malformation, sex, maternal age, parity, maternal smoking, maternal education level and maternal country of birth. Analysis excluded children born 1998 to 2003, leaving 22,272 children.

¶Adjusted for year of birth small for gestational age, large for gestational age, congenital malformation, sex, maternal age, parity, maternal smoking, maternal education level, maternal country of birth and pregnancy BMI. Analysis excluded children with missing data on pregnancy BMI, leaving 33,422 children.

Incidence rate ratios were estimated using negative binomial regression models. Correlation between siblings was accounted for using generalized estimating equations with robust standard errors. CI, confidence intervals. BF, breastfeeding. EBF, exclusive breastfeeding. MO, months. SGA, small for gestation age. LGA, large for gestation age. BMI, body mass index.

For peer review only

Table I: Regression results for lower respiratory infections, early childhood (<2 years)

	Crude*	(95% CI)	Adjusted†	(95% CI)	1998-2003‡	(95% CI)	2004-2010§	(95% CI)	BMI¶	(95% CI)
Breastfeeding :										
No BF	2.39	(1.38–4.13)	2.08	(1.21–3.59)	4.03	(1.79–9.09)	1.25	(0.64–2.45)	2.14	(1.21–3.77)
BF <6 MO and EBF <4 MO	1.36	(1.06–1.76)	1.39	(1.07–1.79)	1.57	(1.01–2.44)	1.28	(0.94–1.76)	1.29	(0.97–1.70)
BF <6 MO with EBF 4–5 MO	1.07	(0.63–1.79)	1.09	(0.65–1.81)	1.12	(0.41–3.07)	1.07	(0.61–1.87)	1.04	(0.60–1.82)
BF ≥6 MO and EBF <4 MO	1.34	(0.99–1.81)	1.38	(1.02–1.86)	1.48	(0.86–2.57)	1.29	(0.90–1.84)	1.27	(0.92–1.75)
BF ≥6 MO with EBF 4–5 MO	0.97	(0.77–1.22)	0.99	(0.79–1.25)	1.15	(0.77–1.72)	0.90	(0.69–1.19)	0.95	(0.75–1.20)
EBF ≥6 MO	1	ref	1	ref	1	ref	1	ref	1	ref
SGA:										
Yes			2.02	(0.96–4.25)	1.52	(0.42–5.57)	2.28	(0.94–5.56)	2.29	(1.09–4.84)
No			1	ref	1	ref	1	ref	1	ref
LGA:										
Yes			1.29	(0.90–1.84)	1.50	(0.83–2.72)	1.18	(0.76–1.83)	1.31	(0.90–1.91)
No			1	ref	1	ref	1	ref	1	ref
Congenital malformation:										
Yes			2.29	(1.50–3.51)	3.26	(1.92–5.54)	1.43	(0.72–2.84)	2.21	(1.40–3.48)
No			1	ref	1	ref	1	ref	1	ref
Sex										
Male			1	ref	1	ref	1	ref	1	ref
Female			0.65	(0.55–0.78)	0.78	(0.56–1.08)	0.59	(0.48–0.73)	0.64	(0.53–0.77)
Maternal age (years):										
≤19			1.48	(0.61–3.58)	1.19	(0.27–5.26)	0	(0.58–5.07)	1.56	(0.59–4.12)
20-24			1	ref	1	ref	1	ref	1	ref
25-29			1.07	(0.77–1.50)	1.38	(0.74–2.56)	0.96	(0.65–1.44)	1.12	(0.78–1.61)
30-34			1.15	(0.82–1.62)	1.52	(0.80–2.87)	1.02	(0.68–1.51)	1.21	(0.83–1.76)
≥35			1.04	(0.71–1.51)	1.40	(0.69–2.84)	0.90	(0.58–1.41)	1.10	(0.73–1.67)
Parity										
1			1	ref	1	ref	1	ref	1	ref
2			2.01	(1.62–2.49)	1.54	(1.03–2.31)	2.29	(1.79–2.94)	1.94	(1.54–2.44)
3			1.99	(1.51–2.62)	1.40	(0.82–2.41)	2.37	(1.74–3.23)	1.91	(1.42–2.56)
≥4			2.60	(1.75–3.87)	2.55	(1.32–4.89)	2.58	(1.58–4.21)	2.71	(1.80–4.08)
Maternal smoking:										

For peer review only

No smoking			1	ref	1	ref	1	ref	1	ref
Smoking			1.47	(1.07–2.02)	1.53	(0.91–2.59)	1.40	(0.94–2.08)	1.43	(1.00–2.03)
Maternal education (years):										
≤9			0.83	(0.58–1.18)	0.91	(0.50–1.63)	0.79	(0.51–1.24)	0.80	(0.55–1.17)
10-12			0.89	(0.73–1.09)	0.96	(0.64–1.45)	0.87	(0.69–1.10)	0.86	(0.69–1.07)
13-14			0.89	(0.68–1.18)	0.94	(0.58–1.51)	0.88	(0.62–1.26)	0.93	(0.70–1.25)
≥15			1	ref	1	ref	1	ref	1	ref
Maternal country of birth:										
Sweden			1	ref	1	ref	1	ref	1	ref
Other Nordic countries			1.15	(0.67–1.95)	1.18	(0.50–2.77)	1.13	(0.57–2.24)	1.03	(0.59–1.82)
Europe and North America			0.54	(0.30–0.96)	0.24	(0.06–0.97)	0.67	(0.36–1.25)	0.61	(0.34–1.08)
Asia			0.86	(0.59–1.26)	0.75	(0.36–1.57)	0.90	(0.59–1.39)	0.82	(0.53–1.26)
Africa			1.44	(0.88–2.36)	1.08	(0.39–2.97)	1.62	(0.92–2.86)	1.53	(0.91–2.55)
Other			1.20	(0.61–2.40)	1.60	(0.52–4.93)	1.08	(0.45–2.56)	0.84	(0.35–1.99)
Year of birth:										
1998	1	ref	1	ref	1	ref			1	ref
1999	1.21	(0.66–2.23)	1.26	(0.68–2.31)	1.27	(0.69–2.33)			1.44	(0.77–2.68)
2000	1.49	(0.85–2.64)	1.55	(0.88–2.73)	1.54	(0.87–2.70)			1.65	(0.90–3.02)
2001	1.53	(0.90–2.59)	1.60	(0.94–2.72)	1.59	(0.94–2.71)			1.62	(0.91–2.87)
2002	1.36	(0.80–2.33)	1.42	(0.83–2.43)	1.41	(0.82–2.41)			1.47	(0.82–2.63)
2003	1.41	(0.84–2.37)	1.50	(0.89–2.52)	1.51	(0.89–2.54)			1.49	(0.86–2.59)
2004	1.56	(0.97–2.53)	1.64	(1.02–2.65)			1	ref	1.80	(1.08–3.00)
2005	2.32	(1.45–3.72)	2.42	(1.51–3.87)			1.48	(1.01–2.16)	2.64	(1.61–4.34)
2006	1.45	(0.88–2.37)	1.52	(0.93–2.48)			0.93	(0.62–1.40)	1.51	(0.89–2.56)
2007	2.29	(1.44–3.64)	2.41	(1.52–3.83)			1.46	(1.01–2.11)	2.34	(1.42–3.86)
2008	1.54	(0.94–2.50)	1.63	(1.00–2.65)			1.00	(0.67–1.48)	1.65	(0.99–2.75)
2009	1.98	(1.22–3.22)	2.10	(1.31–3.38)			1.28	(0.87–1.89)	2.14	(1.29–3.53)
2010	1.87	(1.17–2.98)	2.03	(1.27–3.24)			1.22	(0.84–1.78)	2.03	(1.23–3.34)
Pregancy BMI:										
Underweight									1.33	(0.75–2.37)
Normal									1	ref
Overweight									0.97	(0.78–1.22)
Obese									1.22	(0.93–1.61)
Observations (N):	37,825		37,825		15,553		22,272		33,422	
Clusters (Maternal ID):	25,487		25,487		12,445		16,998		23,605	

For peer review only

*Adjusted for year of birth. Analysis included 37,825 children.

†Adjusted for year of birth small for gestational age, large for gestational age, congenital malformation, sex, maternal age, parity, maternal smoking, maternal education level and maternal country of birth. Analysis included 37,825 children.

‡Included children born 1998 to 2003. Adjusted for year of birth small for gestational age, large for gestational age, congenital malformation, sex, maternal age, parity, maternal smoking, maternal education level and maternal country of birth. Analysis excluded children born 2004 to 2010, leaving 15,553 children.

§Included children born 2004 to 2010. Adjusted for year of birth small for gestational age, large for gestational age, congenital malformation, sex, maternal age, parity, maternal smoking, maternal education level and maternal country of birth. Analysis excluded children born 1998 to 2003, leaving 22,272 children.

¶Adjusted for year of birth small for gestational age, large for gestational age, congenital malformation, sex, maternal age, parity, maternal smoking, maternal education level, maternal country of birth and pregnancy BMI. Analysis excluded children with missing data on pregnancy BMI, leaving 33,422 children.

Incidence rate ratios were estimated using negative binomial regression models. Correlation between siblings was accounted for using generalized estimating equations with robust standard errors. CI, confidence intervals. BF, breastfeeding. EBF, exclusive breastfeeding. MO, months. SGA, small for gestation age. LGA, large for gestation age. BMI, body mass index.

For peer review only

Table J: Regression results for upper respiratory infections, later childhood (2–4 years)

	Crude*(95% CI)		Adjusted†(95% CI)		Adjusted 2‡(95% CI)		1998-2003§(95% CI)		2004-2010¶(95% CI)		BMI#(95% CI)	
Breastfeeding:												
No BF	2.50	(1.30–4.81)	2.25	(1.17–4.33)	2.25	(1.17–4.34)	4.04	(1.82–8.94)	0.93	(0.29–3.00)	2.16	(1.11–4.19)
BF <6 MO and EBF <4 MO	1.38	(0.97–1.95)	1.30	(0.91–1.86)	1.27	(0.88–1.81)	1.65	(1.01–2.68)	0.93	(0.55–1.55)	1.31	(0.89–1.94)
BF <6 MO with EBF 4–5 MO	1.35	(0.76–2.41)	1.33	(0.74–2.37)	1.34	(0.75–2.41)	0.87	(0.37–2.04)	1.90	(0.86–4.19)	1.45	(0.79–2.68)
BF ≥6 MO and EBF <4 MO	1.47	(0.99–2.18)	1.45	(0.97–2.15)	1.42	(0.96–2.11)	1.41	(0.78–2.54)	1.35	(0.79–2.29)	1.52	(1.00–2.30)
BF ≥6 MO with EBF 4–5 MO	1.22	(0.90–1.64)	1.22	(0.90–1.64)	1.21	(0.90–1.63)	1.23	(0.82–1.85)	1.15	(0.74–1.77)	1.23	(0.89–1.69)
EBF ≥6 MO	1	ref	1	ref	1	ref	1	ref	1	ref	1	ref
SGA												
Yes			1.22	(0.58–2.59)	1.19	(0.56–2.53)	1.27	(0.48–3.35)	1.13	(0.36–3.54)	1.20	(0.53–2.71)
No			1	ref	1	ref	1	ref	1	ref	1	ref
LGA												
Yes			1.33	(0.84–2.09)	1.29	(0.82–2.04)	1.19	(0.59–2.37)	1.42	(0.79–2.56)	1.27	(0.80–2.03)
No			1	ref	1	ref	1	ref	1	ref	1	ref
Congenital malformation:												
Yes			2.73	(1.72–4.35)	2.47	(1.56–3.91)	2.33	(1.26–4.28)	2.52	(1.26–5.05)	2.45	(1.49–4.03)
No			1	ref	1	ref	1	ref	1	ref	1	ref
Sex												
Male			1	ref	1	ref	1	ref	1	ref	1	ref
Female			0.67	(0.53–0.84)	0.68	(0.54–0.86)	0.76	(0.55–1.06)	0.61	(0.44–0.84)	0.70	(0.55–0.90)
Maternal age (years):												
≤19			0.38	(0.09–1.57)	0.40	(0.10–1.64)	0.49	(0.04–2.08)	0.60	(0.08–4.60)	0.49	(0.12–2.03)
20-24			1	ref	1	ref	1	ref	1	ref	1	ref
25-29			1.04	(0.71–1.51)	1.04	(0.71–1.52)	0.93	(0.54–1.59)	1.20	(0.69–2.09)	1.08	(0.72–1.63)
30-34			0.92	(0.63–1.36)	0.94	(0.63–1.39)	1.11	(0.65–1.90)	0.81	(0.46–1.43)	1.01	(0.66–1.54)
≥35			1.15	(0.74–1.77)	1.16	(0.75–1.80)	1.23	(0.68–2.22)	1.10	(0.58–2.12)	1.23	(0.77–1.96)
Parity:												
1			1	ref	1	ref	1	ref	1	ref	1	ref
2			1.04	(0.81–1.34)	1.03	(0.80–1.33)	0.93	(0.64–1.33)	1.17	(0.83–1.66)	1.07	(0.81–1.40)
3			0.81	(0.56–1.17)	0.80	(0.56–1.16)	0.57	(0.34–0.97)	1.10	(0.66–1.84)	0.79	(0.53–1.18)
≥4			0.98	(0.59–1.64)	0.98	(0.58–1.63)	0.81	(0.41–1.62)	1.17	(0.54–2.52)	1.09	(0.65–1.85)
Maternal smoking:												

For peer review only

No smoking			1	ref	1	ref	1	ref	1	ref	1	ref
Smoking			1.19	(0.79–1.78)	1.20	(0.80–1.80)	1.15	(0.69–1.89)	1.21	(0.62–2.37)	1.04	(0.66–1.62)
Maternal education (years):												
≤9			1.25	(0.84–1.87)	1.24	(0.83–1.85)	1.51	(0.87–2.61)	0.97	(0.51–1.84)	1.30	(0.85–2.00)
10-12			0.97	(0.73–1.27)	0.97	(0.73–1.27)	1.07	(0.71–1.63)	0.90	(0.62–1.30)	1.04	(0.77–1.41)
13-14			1.23	(0.87–1.72)	1.24	(0.88–1.73)	1.12	(0.68–1.85)	1.44	(0.92–2.27)	1.35	(0.94–1.92)
≥15			1	ref	1	ref	1	ref	1	ref	1	ref
Maternal country of birth:												
Sweden			1	ref	1	ref	1	ref	1	ref	1	ref
Other Nordic countries			0.39	(0.13–1.22)	0.39	(0.12–1.20)	0.47	(0.12–1.89)	0.29	(0.04–2.07)	0.42	(0.14–1.31)
Europe and North America			0.82	(0.44–1.53)	0.83	(0.45–1.55)	0.92	(0.36–2.35)	0.75	(0.33–1.71)	0.62	(0.31–1.26)
Asia			0.84	(0.53–1.34)	0.87	(0.54–1.38)	0.87	(0.46–1.64)	0.87	(0.45–1.68)	0.88	(0.55–1.42)
Africa			0.62	(0.23–1.64)	0.60	(0.23–1.58)	0.70	(0.19–2.60)	0.55	(0.13–2.24)	0.66	(0.25–1.73)
Other			1.37	(0.58–3.27)	1.39	(0.59–3.26)	2.37	(0.93–6.02)	0.54	(0.08–3.79)	1.27	(0.48–3.36)
Year of birth:												
1998	1	ref	1	ref	1	ref			1	ref	1	ref
1999	1.05	(0.61–1.80)	1.09	(0.61–1.80)	1.13	(0.66–1.92)	1.12	(0.66–1.91)			1.20	(0.68–2.10)
2000	1.15	(0.68–1.96)	1.20	(0.68–1.96)	1.23	(0.73–2.09)	1.22	(0.72–2.07)			1.28	(0.73–2.25)
2001	0.94	(0.56–1.59)	0.99	(0.56–1.59)	1.00	(0.60–1.68)	0.98	(0.59–1.64)			1.00	(0.57–1.74)
2002	0.69	(0.40–1.19)	0.71	(0.40–1.19)	0.73	(0.42–1.27)	0.73	(0.42–1.26)			0.64	(0.35–1.18)
2003	0.77	(0.42–1.39)	0.81	(0.42–1.39)	0.84	(0.46–1.52)	0.82	(0.45–1.49)			0.94	(0.50–1.79)
2004	0.83	(0.49–1.41)	0.88	(0.49–1.41)	0.93	(0.55–1.58)	1	ref			1.05	(0.60–1.84)
2005	0.60	(0.34–1.06)	0.63	(0.34–1.06)	0.66	(0.37–1.17)			0.71	(0.41–1.23)	0.68	(0.37–1.24)
2006	0.69	(0.40–1.20)	0.73	(0.40–1.20)	0.76	(0.44–1.32)			0.81	(0.49–1.36)	0.82	(0.46–1.46)
2007	0.74	(0.43–1.28)	0.80	(0.43–1.28)	0.84	(0.49–1.45)			0.91	(0.55–1.53)	0.92	(0.52–1.62)
2008	0.38	(0.20–0.72)	0.40	(0.20–0.72)	0.43	(0.23–0.82)			0.48	(0.26–0.89)	0.48	(0.25–0.93)
2009	0.63	(0.36–1.09)	0.68	(0.36–1.09)	0.73	(0.42–1.27)			0.81	(0.48–1.37)	0.66	(0.36–1.19)
2010	0.48	(0.27–0.86)	0.53	(0.27–0.86)	0.57	(0.32–1.01)			0.63	(0.36–1.10)	0.61	(0.34–1.12)
Previous admissions:												
Yes					4.52	(3.02–6.78)	4.27	(2.66–6.88)	4.94	(2.47–9.89)	4.16	(2.63–6.57)
No					1	ref	1	ref	1	ref	1	ref
Pregancy BMI:												
Underweight											0.44	(0.14–1.34)
Normal											1	ref
Overweight											0.95	(0.73–1.25)

For peer review only

Obese						1.04	(0.71–1.53)
Observations (N):	37,621	37,621	37,621	15,466	22,155	33,236	
Clusters (Maternal ID):	25,344	25,344	25,344	12,377	16,910	23,471	

*Adjusted for year of birth. Analysis included 37,621 children.

†Adjusted for year of birth small for gestational age, large for gestational age, congenital malformation, sex, maternal age, parity, maternal smoking, maternal education level and maternal country of birth. Analysis included 37,621 children.

‡Adjusted for year of birth small for gestational age, large for gestational age, congenital malformation, sex, maternal age, parity, maternal smoking, maternal education level, maternal country of birth and previous admissions. Analysis included 37,621 children.

§Included children born 1998 to 2003. Adjusted for year of birth small for gestational age, large for gestational age, congenital malformation, sex, maternal age, parity, maternal smoking, maternal education level, maternal country of birth and previous admissions. Analysis excluded children born 2004 to 2010, leaving 15,466 children.

¶Included children born 2004 to 2010. Adjusted for year of birth small for gestational age, large for gestational age, congenital malformation, sex, maternal age, parity, maternal smoking, maternal education level, maternal country of birth and previous admissions. Analysis excluded children born 1998 to 2003, leaving 22,155 children.

#Adjusted for year of birth small for gestational age, large for gestational age, congenital malformation, sex, maternal age, parity, maternal smoking, maternal education level, maternal country of birth, previous admissions and pregnancy BMI. Analysis excluded children with missing data on pregnancy BMI, leaving 33,236 children.

Incidence rate ratios were estimated using negative binomial regression models. Correlation between siblings was accounted for using generalized estimating equations with robust standard errors. CI, confidence intervals. BF, breastfeeding. EBF, exclusive breastfeeding. MO, months. SGA, small for gestation age. LGA, large for gestation age. BMI, body mass index.

For peer review only

Table K: Regression results for lower respiratory infections, later childhood (2–4 years)

	Crude*	(95% CI)	Adjusted†	(95% CI)	Adjusted 2‡	(95% CI)	1998-2003§	(95% CI)	2004-2010¶	(95% CI)	BMI#	(95% CI)
Breastfeeding:												
No BF	3.70	(1.76–7.78)	3.50	(1.69–7.24)	3.10	(1.49–6.45)	5.43	(2.33-12.65)	1.28	(0.27–6.12)	3.33	(1.56–7.11)
BF <6 MO and EBF <4 MO	1.79	(1.12–2.86)	1.93	(1.18–3.15)	1.87	(1.15–3.06)	1.62	(0.76–3.44)	2.04	(1.06–3.91)	1.81	(1.07–3.08)
BF <6 MO with EBF 4–5 MO	0.46	(0.16–1.29)	0.51	(0.18–1.42)	0.50	(0.18–1.40)	0.86	(0.25–2.91)	0.24	(0.03–1.78)	0.54	(0.19–1.53)
BF ≥6 MO and EBF <4 MO	1.18	(0.70–2.00)	1.15	(0.67–1.96)	1.13	(0.66–1.93)	1.54	(0.71–3.36)	0.96	(0.47–1.98)	1.15	(0.66–2.03)
BF ≥6 MO with EBF 4–5 MO	0.85	(0.56–1.28)	0.87	(0.58–1.31)	0.88	(0.58–1.32)	0.90	(0.48–1.67)	0.88	(0.51–1.51)	0.86	(0.56–1.32)
EBF ≥6 MO	1	ref	1	ref	1	ref	1	ref	1	ref	1	ref
SGA												
Yes			3.89	(1.86–8.15)	3.86	(1.84–8.10)	1.93	(0.57–6.50)	5.43	(2.29-12.85)	4.17	(1.99–8.74)
No			1	ref	1	ref	1	ref	1	ref	1	ref
LGA												
Yes			0.99	(0.53–1.85)	0.99	(0.53–1.88)	0.87	(0.32–2.40)	1.04	(0.47–2.31)	1.01	(0.51–2.01)
No			1	ref	1	ref	1	ref	1	ref	1	ref
Congenital malformation:												
Yes			5.40	(3.31–8.81)	4.91	(2.96–8.12)	4.47	(2.07–9.66)	4.75	(2.45–9.21)	4.43	(2.62–7.49)
No			1	ref	1	ref	1	ref	1	ref	1	ref
Sex												
Male			1	ref	1	ref	1	ref	1	ref	1	ref
Female			1.00	(0.73–1.36)	1.03	(0.75–1.42)	1.25	(0.78–2.00)	0.87	(0.58–1.31)	1.04	(0.75–1.46)
Maternal age (years):												
≤19			1.10	(0.23–5.16)	1.12	(0.24–5.29)	1.29	(0.27-23.97)	0.82	(0.09–7.22)	1.29	(0.27–6.24)
20-24			1	ref	1	ref	1	ref	1	ref	1	ref
25-29			1.57	(0.87–2.83)	1.53	(0.85–2.76)	2.68	(0.91–7.84)	1.22	(0.60–2.50)	1.66	(0.89–3.09)
30-34			1.38	(0.77–2.46)	1.34	(0.75–2.39)	4.01	(1.37-11.79)	0.69	(0.34–1.36)	1.45	(0.79–2.66)
≥35			1.97	(1.03–3.76)	1.94	(1.02–3.70)	6.07	(1.86-19.81)	0.98	(0.46–2.10)	2.01	(1.02–3.95)
Parity:												
1			1	ref	1	ref	1	ref	1	ref	1	ref
2			1.03	(0.72–1.47)	1.00	(0.69–1.43)	0.76	(0.44–1.30)	1.33	(0.85–2.09)	0.96	(0.66–1.40)
3			1.01	(0.58–1.76)	0.97	(0.55–1.71)	0.55	(0.22–1.39)	1.59	(0.79–3.18)	0.95	(0.51–1.76)
≥4			0.83	(0.42–1.66)	0.79	(0.39–1.60)	0.44	(0.18–1.10)	1.32	(0.50–3.48)	0.79	(0.38–1.65)
Maternal smoking:												

For peer review only

No smoking			1	ref	1	ref	1	ref	1	ref	1	ref
Smoking			0.53	(0.28–1.00)	0.51	(0.27–0.96)	0.52	(0.22–1.22)	0.52	(0.21–1.31)	0.45	(0.23–0.89)
Maternal education (years):												
≤9			0.92	(0.48–1.76)	0.91	(0.47–1.75)	0.88	(0.37–2.11)	0.82	(0.33–2.07)	1.01	(0.51–2.01)
10-12			0.95	(0.65–1.39)	0.95	(0.65–1.39)	1.23	(0.68–2.22)	0.75	(0.46–1.23)	1.00	(0.66–1.50)
13-14			0.99	(0.62–1.59)	0.99	(0.62–1.59)	0.90	(0.45–1.82)	1.07	(0.57–2.03)	0.98	(0.60–1.61)
≥15			1	ref	1	ref	1	ref	1	ref	1	ref
Maternal country of birth:												
Sweden			1	ref	1	ref	1	ref	1	ref	1	ref
Other Nordic countries			1.58	(0.74–3.36)	1.55	(0.73–3.31)	1.42	(0.45–4.51)	1.69	(0.62–4.60)	1.43	(0.63–3.25)
Europe and North America			0.22	(0.05–0.87)	0.22	(0.06–0.90)	0.33	(0.05–2.45)	0.17	(0.02–1.23)	0.24	(0.06–0.98)
Sweden			1.40	(0.79–2.47)	1.41	(0.80–2.51)	1.40	(0.62–3.16)	1.30	(0.58–2.91)	1.57	(0.88–2.80)
Africa			2.05	(0.69–6.06)	2.02	(0.69–5.95)	2.34	(0.79–6.92)	1.77	(0.34–9.14)	1.87	(0.56–6.18)
Other			1.63	(0.37–7.15)	1.64	(0.37–7.30)	3.09	(0.51-18.82)	0.69	(0.10–4.95)	0.50	(0.07–3.61)
Year of birth:												
1998	1	ref	1	ref	1	ref			1	ref	1	ref
1999	0.93	(0.37–2.38)	0.99	(0.37–2.38)	0.99	(0.39–2.53)	0.99	(0.39–2.51)			1.06	(0.40–2.80)
2000	1.26	(0.50–3.13)	1.34	(0.50–3.13)	1.30	(0.53–3.22)	1.21	(0.50–2.91)			1.36	(0.53–3.50)
2001	1.20	(0.46–3.12)	1.27	(0.46–3.12)	1.25	(0.48–3.20)	1.14	(0.46–2.83)			1.06	(0.38–3.02)
2002	1.22	(0.51–2.93)	1.24	(0.51–2.93)	1.19	(0.50–2.86)	1.08	(0.45–2.59)			1.21	(0.48–3.03)
2003	1.29	(0.56–2.96)	1.30	(0.56–2.96)	1.24	(0.54–2.84)	1.13	(0.50–2.58)			1.23	(0.51–2.98)
2004	1.19	(0.47–3.01)	1.25	(0.47–3.01)	1.21	(0.48–3.05)	1	ref			1.31	(0.49–3.49)
2005	1.14	(0.48–2.70)	1.15	(0.48–2.70)	1.06	(0.45–2.49)			0.89	(0.42–1.87)	0.95	(0.39–2.32)
2006	0.80	(0.28–2.27)	0.83	(0.28–2.27)	0.81	(0.28–2.33)			0.67	(0.26–1.74)	0.77	(0.25–2.42)
2007	1.14	(0.49–2.62)	1.14	(0.49–2.62)	1.09	(0.47–2.53)			0.89	(0.43–1.87)	1.19	(0.50–2.83)
2008	1.00	(0.40–2.53)	1.04	(0.40–2.53)	1.02	(0.41–2.54)			0.85	(0.37–1.97)	0.99	(0.38–2.61)
2009	1.46	(0.64–3.32)	1.55	(0.64–3.32)	1.47	(0.65–3.36)			1.23	(0.59–2.59)	1.55	(0.66–3.63)
2010	0.74	(0.31–1.77)	0.83	(0.31–1.77)	0.77	(0.32–1.87)			0.64	(0.29–1.44)	0.79	(0.32–1.97)
Previous admissions:												
Yes					6.29	(3.44-11.50)	9.65	(3.41-27.27)	4.23	(2.15–8.32)	6.58	(3.50-12.38)
No					1	ref	1	ref	1	ref	1	ref
Pregancy BMI:												
Underweight											0.72	(0.21–2.48)
Normal											1	ref
Overweight											0.68	(0.42–1.11)

Obese						1.06	(0.68–1.65)
Observations (N):	37,621	37,621	37,621	15,466	22,155	33,236	
Clusters (Maternal ID):	25,344	25,344	25,344	12,377	16,910	23,471	

*Adjusted for year of birth. Analysis included 37,621 children.

†Adjusted for year of birth small for gestational age, large for gestational age, congenital malformation, sex, maternal age, parity, maternal smoking, maternal education level and maternal country of birth. Analysis included 37,621 children.

‡Adjusted for year of birth small for gestational age, large for gestational age, congenital malformation, sex, maternal age, parity, maternal smoking, maternal education level, maternal country of birth and previous admissions. Analysis included 37,621 children.

§Included children born 1998 to 2003. Adjusted for year of birth small for gestational age, large for gestational age, congenital malformation, sex, maternal age, parity, maternal smoking, maternal education level, maternal country of birth and previous admissions. Analysis excluded children born 2004 to 2010, leaving 15,466 children.

¶Included children born 2004 to 2010. Adjusted for year of birth small for gestational age, large for gestational age, congenital malformation, sex, maternal age, parity, maternal smoking, maternal education level, maternal country of birth and previous admissions. Analysis excluded children born 1998 to 2003, leaving 22,155 children.

#Adjusted for year of birth small for gestational age, large for gestational age, congenital malformation, sex, maternal age, parity, maternal smoking, maternal education level, maternal country of birth, previous admissions and pregnancy BMI. Analysis excluded children with missing data on pregnancy BMI, leaving 33,236 children.

Incidence rate ratios were estimated using negative binomial regression models. Correlation between siblings was accounted for using generalized estimating equations with robust standard errors. CI, confidence intervals. BF, breastfeeding. EBF, exclusive breastfeeding. MO, months. SGA, small for gestation age. LGA, large for gestation age. BMI, body mass index.

For peer review only

STROBE Statement—Checklist of items that should be included in reports of ***cohort studies***

	Item No	Recommendation	Page No
**Title and abstract**	1	(*a*) Indicate the study's design with a commonly used term in the title or the abstract	2
		(*b*) Provide in the abstract an informative and balanced summary of what was done and what was found	3
**Introduction**			
Background/rationale	2	Explain the scientific background and rationale for the investigation being reported	5
Objectives	3	State specific objectives, including any prespecified hypotheses	5
**Methods**			
Study design	4	Present key elements of study design early in the paper	6
Setting	5	Describe the setting, locations, and relevant dates, including periods of recruitment, exposure, follow-up, and data collection	6-8
Participants	6	(*a*) Give the eligibility criteria, and the sources and methods of selection of participants. Describe methods of follow-up	6
		(*b*) For matched studies, give matching criteria and number of exposed and unexposed	
Variables	7	Clearly define all outcomes, exposures, predictors, potential confounders, and effect modifiers. Give diagnostic criteria, if applicable	6-8
Data sources/ measurement	8*	For each variable of interest, give sources of data and details of methods of assessment (measurement). Describe comparability of assessment methods if there is more than one group	6-7
Bias	9	Describe any efforts to address potential sources of bias	8
Study size	10	Explain how the study size was arrived at	-
Quantitative variables	11	Explain how quantitative variables were handled in the analyses. If applicable, describe which groupings were chosen and why	7
Statistical methods	12	(*a*) Describe all statistical methods, including those used to control for confounding	8
		(*b*) Describe any methods used to examine subgroups and interactions	
		(*c*) Explain how missing data were addressed	
		(*d*) If applicable, explain how loss to follow-up was addressed	
		(*e*) Describe any sensitivity analyses	
**Results**			
Participants	13*	(a) Report numbers of individuals at each stage of study—eg numbers potentially eligible, examined for eligibility, confirmed eligible, included in the study, completing follow-up, and analysed	8
		(b) Give reasons for non-participation at each stage	
		(c) Consider use of a flow diagram	
Descriptive data	14*	(a) Give characteristics of study participants (eg demographic, clinical, social) and information on exposures and potential confounders	8 + Tabell 1
		(b) Indicate number of participants with missing data for each variable of interest	Tabell 1
		(c) Summarise follow-up time (eg, average and total amount)	8
Outcome data	15*	Report numbers of outcome events or summary measures over time	8

For peer review only

Main results	16	(*a*) Give unadjusted estimates and, if applicable, confounder-adjusted estimates and their precision (eg, 95% confidence interval). Make clear which confounders were adjusted for and why they were included<br>(*b*) Report category boundaries when continuous variables were categorized<br>(*c*) If relevant, consider translating estimates of relative risk into absolute risk for a meaningful time period	10<br>Appendix B and C
Other analyses	17	Report other analyses done—eg analyses of subgroups and interactions, and sensitivity analyses	Appendix B and C
**Discussion**			
Key results	18	Summarise key results with reference to study objectives	10-11
Limitations	19	Discuss limitations of the study, taking into account sources of potential bias or imprecision. Discuss both direction and magnitude of any potential bias	12-13
Interpretation	20	Give a cautious overall interpretation of results considering objectives, limitations, multiplicity of analyses, results from similar studies, and other relevant evidence	11-13
Generalisability	21	Discuss the generalisability (external validity) of the study results	13
**Other information**			
Funding	22	Give the source of funding and the role of the funders for the present study and, if applicable, for the original study on which the present article is based	14

*Give information separately for exposed and unexposed groups.

Note: An Explanation and Elaboration article discusses each checklist item and gives methodological background and published examples of transparent reporting. The STROBE checklist is best used in conjunction with this article (freely available on the Web sites of PLoS Medicine at http://www.plosmedicine.org/, Annals of Internal Medicine at http://www.annals.org/, and Epidemiology at http://www.epidem.com/). Information on the STROBE Initiative is available at http://www.strobe-statement.org.

For peer review only

BMJ Open

Breastfeeding practice, breastfeeding policy and hospitalisations for infectious diseases in early and later childhood: a register-based study in Uppsala County, Sweden

Journal:	*BMJ Open*
Manuscript ID	bmjopen-2020-046583.R1
Article Type:	Original research
Date Submitted by the Author:	06-Apr-2021
Complete List of Authors:	Videholm, Samuel; Umeå universitet Klinisk vetenskap, unit of Pediatrics<br>Wallby, Thomas; Uppsala University Department of Womens and Childrens Health<br>Silfverdal, Sven-Arne; Umeå universitet Klinisk vetenskap, unit of Pediatrics
**Primary Subject Heading**:	Epidemiology
Secondary Subject Heading:	Public health, Nutrition and metabolism
Keywords:	INFECTIOUS DISEASES, Nutrition < TROPICAL MEDICINE, Public health < INFECTIOUS DISEASES, EPIDEMIOLOGY, Community child health < PAEDIATRICS

SCHOLARONE™
Manuscripts

I, the Submitting Author has the right to grant and does grant on behalf of all authors of the Work (as defined in the below author licence), an exclusive licence and/or a non-exclusive licence for contributions from authors who are: i) UK Crown employees; ii) where BMJ has agreed a CC-BY licence shall apply, and/or iii) in accordance with the terms applicable for US Federal Government officers or employees acting as part of their official duties; on a worldwide, perpetual, irrevocable, royalty-free basis to BMJ Publishing Group Ltd ("BMJ") its licensees and where the relevant Journal is co-owned by BMJ to the co-owners of the Journal, to publish the Work in this journal and any other BMJ products and to exploit all rights, as set out in our licence.

The Submitting Author accepts and understands that any supply made under these terms is made by BMJ to the Submitting Author unless you are acting as an employee on behalf of your employer or a postgraduate student of an affiliated institution which is paying any applicable article publishing charge ("APC") for Open Access articles. Where the Submitting Author wishes to make the Work available on an Open Access basis (and intends to pay the relevant APC), the terms of reuse of such Open Access shall be governed by a Creative Commons licence – details of these licences and which Creative Commons licence will apply to this Work are set out in our licence referred to above.

Other than as permitted in any relevant BMJ Author's Self Archiving Policies, I confirm this Work has not been accepted for publication elsewhere, is not being considered for publication elsewhere and does not duplicate material already published. I confirm all authors consent to publication of this Work and authorise the granting of this licence.

For peer review only

Breastfeeding practice, breastfeeding policy and hospitalisations for infectious diseases in early and later childhood: a register-based study in Uppsala County, Sweden

Samuel Videholm, MD[1], Thomas Wallby, PhD[2], Sven-Arne Silfverdal, MD, PhD, MPH[1]

[1]Department of Clinical Sciences, Pediatrics, Umeå University, Umeå, Sweden

[2]Department of Women's and Children's Health, Uppsala University, Uppsala, Sweden

Corresponding Author:

Name: Samuel Videholm

E-mail: samuel.videholm@umu.se

Telephone number: +46 907858919

Fax: +46 90123728

Postal address:

Department of Clinical Sciences, Pediatrics,

Umeå University

901 87 Umeå

Sweden

Keywords:

Infectious Diseases

Breastfeeding

Public Health

Epidemiology

Community Child Health

Word count: 2806

For peer review only - http://bmjopen.bmj.com/site/about/guidelines.xhtml

Abstract

Objective: To examine the association between breastfeeding practice and hospitalisations for infectious diseases in early and later childhood, i.e. to compare exclusive breastfeeding 4 to 5 months with exclusive breastfeeding 6 months or more. Thereby, provide evidence to inform breastfeeding policy.

Design: A register-based cohort study.

Setting: A cohort was created by combining the Swedish Medical Birth Register, the National Inpatient Register, the Cause of Death Register, the Total Population Register, the Longitudinal integration database for health insurance and labour market studies, with the Uppsala Preventive Child Health Care database.

Patients: 37 825 term and post-term singletons born to women who resided in Uppsala County (Sweden) between 1998 and 2010.

Main outcome measures: Number of hospitalisations for infectious diseases in early (<2 years) and later childhood (2–4 years).

Results: The risk of hospitalisations for infectious diseases decreased with duration of exclusive breastfeeding until 4 months of age. In early childhood, breastfeeding was associated with a decreased risk of enteric and respiratory infections. In comparison with exclusive breastfeeding 6 months or more, the strongest association was found between no breastfeeding and enteric infections (aIRR 3.32 [95% CI 2.14–5.14]). In later childhood, breastfeeding was associated with a lower risk of respiratory infections. In comparison with children exclusively breastfed 6 months or more, the highest risk was found in children who were not breastfed (aIRR 2.53 [95% CI 1.51–4.24]). The risk of hospitalisations for infectious diseases was comparable in children exclusively breastfed 4 to 5 months and children exclusively breastfed 6 months or more.

Conclusions: Our results support breastfeeding guidelines that recommend exclusive breastfeeding for at least 4 months.

For peer review only

For peer review only - http://bmjopen.bmj.com/site/about/guidelines.xhtml

Strengths and limitations of this study:

This register-based cohort study included almost all (<10% missing data) term and post-term singletons born in a geographical region over 13 years, which reduces the risk of selection bias.

Children were followed from birth until 5 years of age or censoring, which enabled us to examine the effect of breastfeeding in both early (<2 years) and later childhood (2–4 years).

Information on pregnancy, birth and sociodemographic characteristics were obtained from high-quality health and administrative registers, which allowed us to adjust analyses for several potential confounders including congenital malformation, large/small for gestational age, maternal age, maternal smoking, parity, maternal education level and maternal country of birth.

Infections were identified using de-identified hospital discharge data, cases could not be confirmed by information obtained from medical records.

Breastfeeding practice was not continuously collected, information on breastfeeding practice was only available at 1 week, 2, 4 and 6 months.

For peer review only

For peer review only - http://bmjopen.bmj.com/site/about/guidelines.xhtml

BACKGROUND

Infectious diseases cause large harm and suffering in childhood. Globally, infectious diseases are a leading cause of death in children under five years of age.[1] In high-income countries, infectious disease remains a major cause of hospitalisations in young children.[2 3] Breast milk contains IgA antibodies and oligosaccharides that prevent microbes to adhere to the mucosa, lactoferrin and lysozyme act directly on the microbes, and growth factors, nucleotides, cytokines stimulate maturation of the infant's immune system.[4 5] Numerous studies have shown that breastfeeding reduces the risk of infectious diseases in infancy.[4 6 7] A few studies have also reported that breastfeeding reduces the risk of infectious diseases after infancy.[8-10]

Although it is well known that breastfeeding protects young children in high-income countries against infections, the optimal breastfeeding practice is still under debate. The World Health Organization recommends exclusive breastfeeding until 6 months of age followed by breastfeeding along with complementary feeding.[11] The American Academy of Pediatrics recommends breastfeeding for at least 12 months with exclusive breastfeeding for about 6 months.[12] In contrast, the European Society for Paediatric Gastroenterology, Hepatology, and Nutrition recommend exclusive breastfeeding for at least 4 months with exclusive or predominant breastfeeding for approximately 6 months.[6] The aim of this study was to examine the association between breastfeeding practice and hospitalisations for infectious diseases in early (<2 years) and later childhood (2–4 years), i.e. to compare exclusive breastfeeding 4 to 5 months with exclusive breastfeeding 6 months or more.

METHODS

Patient and Public Involvement

This study was conducted without patient involvement.

Setting

For peer review only - http://bmjopen.bmj.com/site/about/guidelines.xhtml

In Sweden, all children are offered free primary and hospital care, a free preventive child health care program and a free general immunisation program; working parents are entitled to a generous parental leave scheme.[13] During the study period, Swedish guidelines for breastfeeding duration were consistent with the World Health Organization recommendations. Until 2002, exclusive breastfeeding was recommended until four to six months of age. Thereafter, exclusive breastfeeding was recommended for at least 6 months.[14]

Study population

This is a register-based cohort study of term and post-term (gestational age 37 weeks or more) singletons born to women who resided in Uppsala County between 1998 and 2010. A database was created by combining data from nationwide and local health and administrative registers. The Swedish Medical Birth Register includes information on prenatal, delivery and neonatal care, with coverage of 98–99 percent of all births.[15] The National Patient Register contains information on all hospitalisations including International Classification of Disease, Tenth Revision (ICD-10) codes for primary diagnosis.[16] The Longitudinal integration database for health insurance and labour market studies, includes information on maternal education.[17] The Cause of Death Register provides information on mortality. The Total Population Register contains information about migration and maternal country of birth. The Child Health Care Quality database in Uppsala contains data on breastfeeding, which was collected by child health nurses during free routine health checkups.[13] These registers were linked using the unique personal identification number assigned to every Swedish resident, by the Centre for Epidemiology at the Swedish National Board of Health and Welfare. All data were anonymised and de-identified prior to analysis.

Main Exposures

Breastfeeding was categorised according to intensity and duration. Information on current breastfeeding status (exclusive breastfeeding, partial breastfeeding or no breastfeeding) was collected at 1 week, 2, 4 and 6 months. Breastfeeding was categorised as no breastfeeding if “no

For peer review only - http://bmjopen.bmj.com/site/about/guidelines.xhtml

breastfeeding" was reported at 1 week. Thereafter, exclusive breastfeeding was considered to be present until "partial breastfeeding" or "no breastfeeding" was reported; breastfeeding was considered to be present until "no breastfeeding" was reported e.g. breastfeeding was categorised as "exclusive breastfeeding 4–5 months with breastfeeding ≥6 months" if "exclusive breastfeeding" was reported at 1 week, 2 months, 4 months and "partial breastfeeding" was reported at 6 months. Information on breastfeeding intensity and duration was combined into the following categories: No breastfeeding (No BF), exclusive breastfeeding <4 months with breastfeeding <6 months (EBF <4 MO with BF <6 MO), exclusive breastfeeding <4 months with breastfeeding ≥6 months (EBF <4 MO with BF ≥6 MO), exclusive breastfeeding 4–5 months with breastfeeding <6 months (EBF 4–5 MO with BF <6 MO), exclusive breastfeeding 4–5 months with breastfeeding ≥6 months (EBF 4–5 MO with BF ≥6 MO) and exclusive breastfeeding ≥6 months (EBF ≥6 MO). Before 2004, the Swedish definition of exclusive breastfeeding allowed ''small tastes'' that didn't replace a breast milk feed. In 2004 the WHO definition of exclusive breastfeeding was adopted in Sweden, and tastes were no longer accepted to fulfil this criterion.[18 19]

Covariates

Analyses were adjusted for several child and maternal characteristics. Information on small for gestational age (yes or no), large for gestational age (yes or no), congenital malformation (ICD-10 codes Q00-Q99) and sex (male or female), maternal age (≤19, 20–24, 25–29, 30–34, and ≥35), parity (1, 2, 3, and ≥4), maternal smoking at the first antenatal care visit (yes or no) and year of birth were retrieved from the Medical Birth Register. Maternal education level at year of birth (secondary school or less [≤9 years], upper secondary school [10–12 years], short postsecondary education [13–14 years] and long postsecondary education [≥15 years]) was obtained from the Longitudinal integration database for health insurance and labour market studies. Information on maternal country of birth (Sweden, Other Nordic, Other Europe and North America, Asia, Africa and Other) was retrieved from the Total Population Register.

For peer review only - http://bmjopen.bmj.com/site/about/guidelines.xhtml

Outcomes

The main outcome was overall number of hospitalisations with a principal diagnosis of infectious disease recorded in the National Patient Register. Secondary outcomes were number of hospitalisations for respiratory tract and enteric infections. Hospitalisations were recorded using ICD-10 codes. We used a previously developed coding scheme to identify infectious disease codes and group them into infectious disease categories (online supplementary appendix A).[3]

Statistical analysis

Crude and adjusted associations between breastfeeding practice and number of hospitalisations were estimated using negative binomial regression models. Log follow-up time (days) was used as an offset. Results were presented as adjusted incidence rate ratios (aIRRs) with 95% confidence intervals (CIs). Correlations between siblings were accounted for using generalized estimating equations with robust standard errors.[20] All analyses were adjusted for time trends (year of birth). The adjusted analyses were also controlled for small for gestational age, large for gestational age, congenital malformation, sex, maternal age, parity, maternal smoking, maternal education level and maternal country of birth. Crude and adjusted models were fitted for each outcome (overall, respiratory and enteric infections) and follow-up period (early childhood [<2 years] and later childhood [2–4 years]). In the later follow-up period, an additional adjusted model controlled for previous admissions (a binary variable indicating hospitalisation in the early follow-up period) was fitted for each outcome. All models were restricted to observations with complete data on breastfeeding and covariates. In sensitivity analyses, the respiratory category was divided into upper and lower respiratory infections, analyses were adjusted for maternal body mass index (BMI) during early pregnancy and analyses were stratified by year of birth (1998 to 2003 and 2004 to 2010). Maternal BMI was excluded from the original analyses due to a large proportion of missing data (12%). All statistical analyses were performed using Stata V.14 (Stata, 2015. Stata Statistical Software: Release 14. College Station, Texas, USA).

For peer review only - http://bmjopen.bmj.com/site/about/guidelines.xhtml

Ethics

The project was approved by the Regional Ethics Board in Umeå (reference number 2012-265-31M and 2017-399-32M).

RESULTS

The Swedish Medical Birth Register included 41 825 term and post-term singletons born to women who resided in Uppsala County between 1998 and 2010. We excluded children with missing data on breastfeeding (n=2127) or covariates (n=1873), leaving 37 825 children (90% of the original cohort). In the first follow-up period (early childhood), all children were followed from birth until 2 years of age or censoring due to death (n=10) or emigration (n=194). In the later follow-up period (later childhood), the remaining 37 621 children were followed from 2 until 5 years of age or censoring due to death (n=14) or emigration (n=573). The study included 187 763 person-years (PY) of follow up time, 75 513 PY in early childhood and 112 250 PY in later childhood. In total 4728 hospital admissions for infectious diseases were recorded during the study period. Readmissions on the same day recorded with the same infectious disease group were excluded (n=23) leaving 4705 admissions. In early childhood, the study included 1485 admissions for respiratory infections (799 admissions for upper respiratory infections and 686 admissions for lower respiratory infections), 762 admissions for enteric infections and 1080 admissions for other infections. In later childhood, the study included 630 admissions for respiratory infections (381 admissions for upper respiratory infections and 249 admissions for lower respiratory infections), 300 admissions for enteric infections and 448 admissions for other infections.

Table 1 presents maternal and child characteristics. Most mothers initiated breastfeeding, only 1.6% (n=613) of mothers reported no breastfeeding at one week. A majority of women exclusively breastfed for at least 4 months, exclusive breastfeeding 4–5 months with breastfeeding <6 months was reported by 3.9%, exclusive breastfeeding 4–5 months with breastfeeding ≥6 months was reported by 40% and exclusive breastfeeding ≥6 months was reported by 25%. Women over 25 years

For peer review only - http://bmjopen.bmj.com/site/about/guidelines.xhtml

of age and women with a postsecondary education, were more likely to report exclusive breastfeeding for 4 months or more. In contrast, women who reported smoking during pregnancy were more likely to report no breastfeeding at one week.

Figure 1 shows the association between breastfeeding categories and overall hospitalisations for infectious disease in early and later childhood. The risk of hospitalisations for infections decreased with duration of exclusive breastfeeding until 4 months of age. In comparison with children exclusively breastfed 6 months or more, the highest risk of hospitalisations for infectious diseases in early childhood was found in children who were not breastfed (aIRR 1.89 [95% CI 1.45–2.47]) and in children exclusive breastfed <4 months with breastfeeding <6 months (aIRR 1.41 [95% CI 1.25–1.59]). Similarly, the risk of hospitalisations for infectious diseases in later childhood was highest in children who were not breastfed (aIRR 1.52 [95% CI 1.00–2.33], controlled for previous admissions) and in children breastfed <6 months and exclusive breastfeeding <4 months (aIRR 1.43 [95% CI 1.16–1.75], controlled for previous admissions). The risk of hospitalisations for infectious diseases was comparable in children exclusively breastfed 4 to 5 months and children exclusively breastfed 6 months or more. Online supplementary appendix B includes full regression results including sensitivity analyses.

Figure 2 shows associations between breastfeeding categories and hospitalisations for respiratory tract and enteric infections in early and later childhood. In early childhood, the risk of both respiratory and enteric infections decreased with duration of exclusive breastfeeding until 4 months of age. In comparison with exclusive breastfeeding 6 months or more, the strongest association was found between no breastfeeding and enteric infections (aIRR 3.32 [95% CI 2.14–5.14]). In later childhood, the risk of respiratory infections decreased with duration of exclusive breastfeeding until 4 months of age. In comparison with children exclusively breastfed 6 months or more, the highest risk of respiratory infections was observed in children who were not breastfed (aIRR 2.53 [95% CI

For peer review only - http://bmjopen.bmj.com/site/about/guidelines.xhtml

1.51–4.24], controlled for previous admissions). Online supplementary appendix C includes full regression results including sensitivity analyses.

Sensitivity analyses showed no substantial changes in point estimates after adjusting for pregnancy BMI. Moreover, sensitivity analyses showed similar associations between breastfeeding categories and overall hospitalisations for infectious disease in the first (1998–2003) and second (2004–2010) birth cohort. In sex-stratified analyses, associations between breastfeeding categories and overall hospitalisations for infectious disease were similar in boys and girls (data not shown).

DISCUSSION

We found that the risk of overall hospitalisations for infectious diseases in early childhood (<2 years) and the risk of hospitalisations for respiratory infections in later childhood (2–4 years) decreased with duration of exclusive breastfeeding until 4 months of age. In comparison with exclusive breastfeeding 6 months or more, exclusive breastfeeding 4 to 5 months was not associated with a higher risk of enteric or respiratory infections.

There is strong evidence that breastfeeding protects infants from infectious diseases.[4 6 7] However, the optimal breastfeeding practice is under discussion. Our results are consistent with a Spanish study reporting that full breastfeeding until at least 4 months would prevent 56.4% (95% CI: 30.9%–69.4%) of non-perinatal infection in the first year of life.[21] In comparison with no breastfeeding, a Dutch study found that exclusive breastfeeding until 4 months followed by partially breastfeeding decreased the risk of physician-confirmed upper respiratory (adjusted odds ratio [aOR] 0.65 [95% CI: 0.51–0.83]) lower respiratory (aOR 0.50 [95% CI: 0.32–0.79]) and gastrointestinal infections (aOR 0.41 [95% CI: 0.26–0.64]) during the first 6 months. However, the authors concluded that exclusive breastfeeding until 6 months may reduce the risk of infectious diseases even more.[22] In contrast to our findings, a US study reported that full breastfeeding for 6 months was, in comparison with full breastfeeding for 4 months, associated with a decreased risk of respiratory tract infection during the first two years of life.[23] Moreover, observational analysis of the PROBIT trial found that exclusive

For peer review only - http://bmjopen.bmj.com/site/about/guidelines.xhtml

breastfeeding for 6 months was, compared with exclusive breastfeeding for 3 months and partial breast-feeding thereafter, associated with a decreased risk of gastrointestinal episodes; no significant association was found for gastrointestinal hospitalisations.[7] The Dutch and US studies included less severe infections, whereas our study only included infections requiring hospital care.

There is increasing evidence that breastfeeding also reduces the risk of respiratory infections after infancy. The mechanism is still unknown, however, it has been suggested that breastmilk influence the development of the immune system.[8 24] Our findings are similar to those reported in a recent Japanese study. In comparison with no breastfeeding, exclusive breastfeeding at 6–7 months of age was associated with a reduced risk of hospitalisations for respiratory tract infections (aOR 0.76 [95% CI: 0.58–0.99]) between 30 and 42 months of age, whereas no association was found between breastfeeding and hospitalisations for diarrhea.[9] In a Dutch cohort study, breastfeeding for at least 6 months was in comparison with no breastfeeding associated with a decreased risk lower respiratory tract infections (aOR 0.71 [95% CI: 0.51–0.98]) between infancy and 4 years of age.[10] Moreover, a recent US study of children aged 6 years, found that any breastfeeding for 9 months or more was in comparison with any breastfeeding for >0 to < 3 months associated with a decreased risk of ear (aOR 0.69 [95% CI: 0.48–0.98]), throat (aOR 0.68 [95% CI: 0.47–0.98]), and sinus (aOR 0.47 [95% CI: 0.30–0.72]) infections, but not with cold/upper respiratory tract infections and pneumonia.[8] Overall, these findings are consistent with our results, that breastfeeding reduces the risk of respiratory infections after infancy.

Our study has several strengths. First, the use of high-quality health and administrative registers allowed us to adjust analyses for potential confounders including congenital malformation, large/small for gestational age, maternal age, maternal smoking, maternal education level and maternal country of birth. Secondly, the long follow up period (until 5 years of age or censoring) enabled us to examine the effect of breastfeeding in both early and later childhood. Additionally, this allowed analyses in later childhood to be adjusted for hospitalisations in early childhood. Finally, our

For peer review only - http://bmjopen.bmj.com/site/about/guidelines.xhtml

study included almost all children (<10% missing data) in one county, thereby reducing the risk of selection bias. However, our study has several weaknesses. In early childhood, infections episodes may influence breastfeeding practice leading to reverse causation. In later childhood, confidence intervals were wide due to the small number of events. Therefore, the associations between breastfeeding and infections in later childhood need to be interpreted with caution. Information on current breastfeeding status was not continuously collected. Compared to studies with complete information on breastfeeding practice since birth e.g. collected through daily recordings, our study is likely to overestimate the proportion of exclusively breastfed children.[25] Moreover, Sweden has family-friendly policies that enable breastfeeding including a generous parental leave scheme.[13] Consequently, our findings may not be generalised to other populations with less family-friendly policies and lower breastfeeding rates. Finally, due to the observational nature of this study, we cannot rule out the risk of unmeasured or residual confounding.

CONCLUSIONS

Our study found that the risk of overall hospitalisations for infectious diseases in early childhood and the risk of hospitalisations for respiratory infections in later childhood decreased with duration of exclusive breastfeeding until 4 months of age. Additionally, the risk of hospitalisations for infectious diseases was comparable in children exclusively breastfed 4 to 5 months and children exclusively breastfed 6 months or more. Thereby, it supports the current European Society for Paediatric Gastroenterology, Hepatology, and Nutrition guidelines, recommending exclusive breastfeeding for at least 4 months with exclusive or predominant breastfeeding for approximately 6 months. Moreover, it adds to the growing body of evidence suggesting a protective effect of breastfeeding on respiratory infections after infancy.

For peer review only - http://bmjopen.bmj.com/site/about/guidelines.xhtml
For peer review only

Contributors: SV conceptualised and designed the study together with SAS, carried out analyses and drafted the initial manuscript. TW supervised the collection of breastfeeding data, contributed to the study design, reviewed and revised the manuscript. SAS conceptualised and designed the study together with SV, coordinated and supervised the database, reviewed and revised the manuscript.

Funding: This study was supported by the Unit of Research, Development, and Education, Östersund Hospital, Östersund, Sweden (JLL-930202 [to SV]); ALF Umeå University, Umeå, Sweden (RV-933162 [to SAS]).

Competing interests: We have no conflict of interest to declare.

Patient and Public Involvement: This study was conducted without patient involvement.

Patient consent: Patient informed consent was not needed because the data were collected retrospective and anonymised prior to analysis.

Ethics approval: The project was approved by the Regional Ethics Board in Umeå (reference number 2012-265-31M and 2017-399-32M).

Acknowledgement: The authors would like to thank Anna Lindam, PhD at the Unit of Research, Development, and Education, Östersund Hospital, Sweden, for assistance with statistical analysis and Håkan Sjöberg, MEc at Statistics Sweden, Örebro, Sweden, for help in compiling the dataset.

Data availability statement: We used deidentified register data obtained from third parties. It includes sensitive information and some access restrictions may apply. Interested researchers need to obtain data directly from the National Board of Health and Welfare in Sweden (socialstyrelsen@socialstyrelsen.se), from Statistics Sweden (scb@scb.se) and from the Child Health Service Unit in Uppsala (thomas.wallby@kbh.uu.se).

Children included in the study were identified in the Medical Birth Register, data on hospitalisations were obtained from the Swedish National Patient Register and data on deaths were obtained from the Cause of Death Register. All of these registers are maintained by the National Board of Health

For peer review only - http://bmjopen.bmj.com/site/about/guidelines.xhtml

and Welfare in Sweden. Data on maternal education were obtained from the Longitudinal Integration Database for Health Insurance and Labour market Studies and data on migration were obtained from The Total Population Register, both registers are maintained by Statistics Sweden. Data on breastfeeding was obtained from The Child Health Care Quality database, which is maintained by the Child Health Service Unit in Uppsala.

For peer review only

For peer review only - http://bmjopen.bmj.com/site/about/guidelines.xhtml

REFERENCES

1 Collaborators GBDCoD. Global, regional, and national age-sex-specific mortality for 282 causes of death in 195 countries and territories, 1980-2017: a systematic analysis for the Global Burden of Disease Study 2017. *Lancet* 2018;392:1736-88.
2 Yorita KL, Holman RC, Sejvar JJ, Steiner CA, Schonberger LB. Infectious disease hospitalizations among infants in the United States. *Pediatrics* 2008;121:244-52.
3 Baker MG, Barnard LT, Kvalsvig A*, et al.* Increasing incidence of serious infectious diseases and inequalities in New Zealand: a national epidemiological study. *Lancet* 2012;379:1112-9.
4 Duijts L, Ramadhani MK, Moll HA. Breastfeeding protects against infectious diseases during infancy in industrialized countries. A systematic review. *Maternal & child nutrition* 2009;5:199-210.
5 Horta BL, Victora, C. G. . Short-term eff ects of breastfeeding: a systematic review of the benefi ts of breastfeeding on diarhoea and pneumonia mortality. Geneva: World Health Organization. 2013.
6 Fewtrell M, Bronsky J, Campoy C*, et al.* Complementary Feeding: A Position Paper by the European Society for Paediatric Gastroenterology, Hepatology, and Nutrition (ESPGHAN) Committee on Nutrition. *J Pediatr Gastroenterol Nutr* 2017;64:119-32.
7 Kramer MS, Kakuma R. Optimal duration of exclusive breastfeeding. *Cochrane Database Syst Rev* 2012:CD003517.
8 Li R, Dee D, Li CM, Hoffman HJ, Grummer-Strawn LM. Breastfeeding and risk of infections at 6 years. *Pediatrics* 2014;134 Suppl 1:S13-20.
9 Yamakawa M, Yorifuji T, Kato T*, et al.* Long-Term Effects of Breastfeeding on Children's Hospitalization for Respiratory Tract Infections and Diarrhea in Early Childhood in Japan. *Matern Child Health J* 2015;19:1956-65.
10 Tromp I, Kiefte-de Jong J, Raat H*, et al.* Breastfeeding and the risk of respiratory tract infections after infancy: The Generation R Study. *PloS one* 2017;12:e0172763.
11 World Health Organization. The Optimal Duration of Exclusive Breastfeeding: Report of an Expert Consultation. 2001.
12 Pediatrics AAo. Breastfeeding and the use of human milk. *Pediatrics* 2012;129.
13 Wettergren B, Blennow M, Hjern A, Soder O, Ludvigsson JF. Child Health Systems in Sweden. *The Journal of pediatrics* 2016;177S:S187-S202.
14 The National Board of Health and Welfare in Sweden. Statistics on Breastfeeding 2014. Available at: https://www.socialstyrelsen.se/globalassets/sharepoint-dokument/artikelkatalog/statistik/2016-9-20.pdf (accessed September 7, 2020).
15 The National Board of Health and Welfare in Sweden. The Swedish Medical Birth Register - A summary of content and quality; 2003.
16 Ludvigsson JF, Andersson E, Ekbom A*, et al.* External review and validation of the Swedish national inpatient register. *BMC public health* 2011;11:450.
17 Ludvigsson JF, Svedberg P, Olen O, Bruze G, Neovius M. The longitudinal integrated database for health insurance and labour market studies (LISA) and its use in medical research. *European journal of epidemiology* 2019;34:423-37.
18 . The National Board of Health and Welfare in Sweden. Kvalitetsdeklaration Statistik om amning 2016. Available at: https://www.socialstyrelsen.se/globalassets/sharepoint-dokument/artikelkatalog/statistik/2018-9-2-kvalitetsdeklaration.pdf (accessed September 7, 2020).
19 . The National Board of Health and Welfare in Sweden. Statistics on breastfeeding 2017. Available at: https://www.socialstyrelsen.se/globalassets/sharepoint-dokument/artikelkatalog/statistik/2019-9-6379.pdf (accessed September 7, 2020).
20 Hanley JA, Negassa A, Edwardes MD, Forrester JE. Statistical analysis of correlated data using generalized estimating equations: an orientation. *American journal of epidemiology* 2003;157:364-75.
21 Paricio Talayero JM, Lizan-Garcia M, Otero Puime A*, et al.* Full breastfeeding and hospitalization as a result of infections in the first year of life. *Pediatrics* 2006;118:e92-9.

For peer review only - http://bmjopen.bmj.com/site/about/guidelines.xhtml

22 Duijts L, Jaddoe VW, Hofman A, Moll HA. Prolonged and exclusive breastfeeding reduces the risk of infectious diseases in infancy. *Pediatrics* 2010;126:e18-25.
23 Chantry CJ, Howard CR, Auinger P. Full breastfeeding duration and associated decrease in respiratory tract infection in US children. *Pediatrics* 2006;117:425-32.
24 Hanson LA, Silfverdal SA. The mother's immune system is a balanced threat to the foetus, turning to protection of the neonate. *Acta paediatrica* 2009;98:221-8.
25 Aarts C, Kylberg E, Hornell A, Hofvander Y, Gebre-Medhin M, Greiner T. How exclusive is exclusive breastfeeding? A comparison of data since birth with current status data. *International journal of epidemiology* 2000;29:1041-6.

For peer review only

For peer review only - http://bmjopen.bmj.com/site/about/guidelines.xhtml

Table 1. Child and maternal characteristics and their association with breastfeeding categories

	Not BF	EBF <4 MO with BF <6 MO	EBF <4 MO with BF ≥6 MO	EBF 4–5 MO with BF <6 MO	EBF 4–5 MO with BF ≥6 MO	EBF ≥6 MO	All included	Excluded
	% (n)	% (n)	% (n)	% (n)	% (n)	n	n	
All	1.6 (613)	19 (7335)	11 (3998)	3.9 (1488)	40 (15007)	25 (9384)	37825	4000
SGA								
Yes	1.5 (8)	27 (144)	16 (86)	4.1 (22)	32 (170)	20 (106)	536	85
No	1.6 (605)	19 (7191)	11 (3912)	3.9 (1466)	40 (14837)	25 (9278)	37289	3816
LGA								
Yes	2.5 (43)	22 (385)	11 (189)	3.5 (62)	35 (614)	26 (457)	1750	172
No	1.6 (570)	19 (6950)	11 (3809)	4.0 (1426)	40 (14393)	25 (8927)	36075	3729
Congenital malformation*								
Yes	4.0 (32)	25 (196)	9.7 (77)	3.6 (29)	35 (277)	23 (186)	797	3891
No	1.6 (581)	19 (7139)	11 (3921)	3.9 (1459)	40 (14730)	25 (9198)	37028	109
Sex								
Male	1.6 (319)	20 (3926)	11 (2077)	4.1 (795)	40 (7751)	24 (4731)	19599	2088
Female	1.6 (294)	19 (3409)	11 (1921)	3.8 (693)	40 (7256)	26 (4653)	18226	1912
Maternal age								
≤19	2.5 (10)	47 (187)	7.5 (30)	8.2 (33)	24 (98)	11 (43)	401	146
20-–24	1.6 (67)	33 (1402)	9.0 (378)	6.7x (282)	32 (1362)	17 (723)	4214	607
25–29	1.7 (198)	21 (2366)	9.8 (1136)	4.7 (545)	40 (4582)	24 (2727)	11554	1167
30–34	1.6 (222)	15 (2108)	11 (1482)	3.4 (470)	43 (5876)	27 (3682)	13840	1305
≥35	1.5 (116)	16 (1272)	12 (972)	2.0 (158)	40 (3089)	28 (2209)	7816	775
Parity								
1	1.1 (185)	22 (3510)	12 (1878)	3.9 (642)	39 (6384)	23 (3724)	16323	1821
2	1.9 (268)	18 (2486)	9.3 (1301)	4.3 (603)	41 (5738)	26 (3584)	13980	1336
3	2.1 (110)	17 (902)	10 (541)	3.1 (165)	41 (2177)	27 (1440)	5335	535
≥4	2.3 (50)	20 (437)	13 (278)	3.6 (78)	32 (708)	29 (636)	2187	308

For peer review only

Maternal smoking								
No smoking	1.5 (521)	18 (6136)	11 (3744)	3.8 (1337)	41 (14362)	26 (9061)	35161	2366
Smoking	3.5 (92)	45 (1199)	9.5 (254)	5.7 (151)	24 (645)	12 (323)	2664	255
Maternal education†								
≤9	2.9 (102)	36 (1273)	11 (406)	5.4 (193)	29 (1014)	16 (561)	3549	419
10–12	2.2 (338)	25 (3942)	10 (1590)	5.4 (838)	35 (5516)	22 (3378)	15602	1217
13–14	1.1 (56)	15 (725)	10 (500)	3.2 (161)	42 (2071)	30 (1472)	4985	491
≥14	0.9 (117)	10 (1395)	11 (1502)	2.2 (296)	47 (6406)	29 (3973)	13689	1217
Maternal country of birth								
Sweden	1.6 (526)	19 (6231)	9.9 (3168)	4.1 (1313)	40 (12853)	25 (7977)	32068	2693
Other Nordic	0.8 (6)	17 (134)	8.8 (70)	2.4 (19)	41 (326)	30 (238)	793	128
Other Europe and North America	1.8 (27)	20 (303)	12 (185)	3.2 (48)	37 (564)	25 (382)	1509	339
Asia	1.3 (33)	22 (538)	16 (399)	3.0 (75)	36 (895)	21 (528)	2468	540
Africa	1.9 (12)	11 (67)	20 (126)	3.1 (19)	38 (233)	26 (163)	620	224
Other	2.5 (9)	17 (62)	14 (50)	3.8 (14)	37 (136)	26 (96)	367	73

Children with missing data on breastfeeding (n=2127) or covariates (n=1873) were excluded leaving 37825 children.

*ICD-10 codes: Q00-Q99.

†Maternal education level at year of birth, categorised as 9 years or less, 10–12 years, 13–14 years or 15 years or more.

BF, breastfeeding. EBF, exclusive breastfeeding. MO, Months. SGA, small for gestation age. LGA, large for gestation age.

For peer review only - http://bmjopen.bmj.com/site/about/guidelines.xhtml

Figure 1. Crude analyses (blue circles), adjusted analyses (red squares) and adjusted analyses controlled for previous admissions (green triangles) of the associations between breastfeeding categories and overall hospitalisations for infectious disease, in early childhood (<2 years) and later childhood (2–4 years). Incidence rate ratios are presented on a logarithmic scale. Vertical lines represent 95% confidence intervals around the point estimates. BF, breastfeeding. EBF exclusive breastfeeding. MO, months.

Figure 2. Crude analyses (blue circles), adjusted analyses (red squares) and adjusted analyses controlled for previous admissions (green triangles) of the associations between breastfeeding categories, and hospitalisations for respiratory tract and enteric infections, in early childhood (<2 years) and later childhood (2–4 years). Incidence rate ratios are presented on a logarithmic scale. Vertical lines represent 95% confidence intervals around the point estimates. BF, breastfeeding. EBF exclusive breastfeeding. MO, months.

For peer review only

For peer review only - http://bmjopen.bmj.com/site/about/guidelines.xhtml

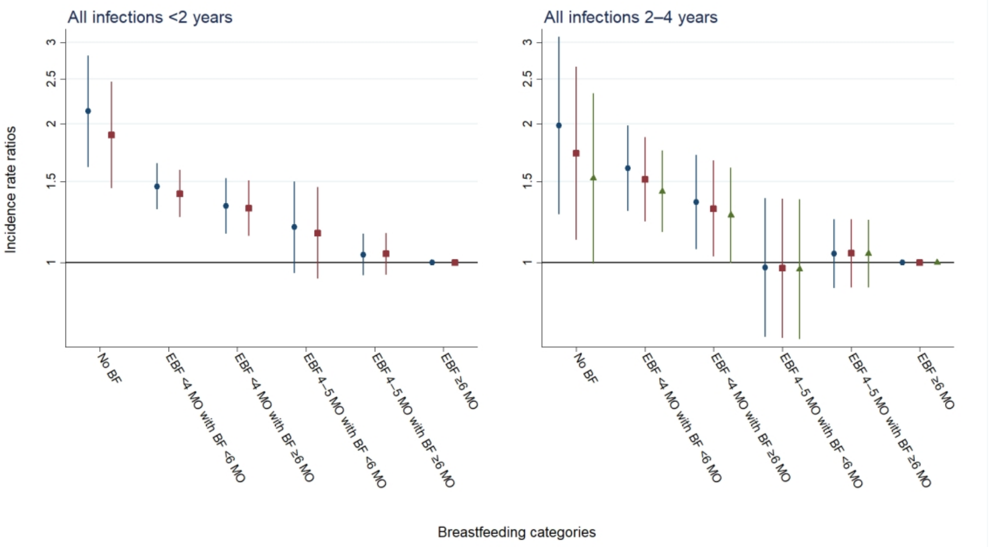

Crude analyses (blue circles), adjusted analyses (red squares) and adjusted analyses controlled for previous admissions (green triangles) of the associations between breastfeeding categories and overall hospitalisations for infectious disease, in early childhood (<2 years) and later childhood (2–4 years). Incidence rate ratios are presented on a logarithmic scale. Vertical lines represent 95% confidence intervals around the point estimates. BF, breastfeeding. EBF exclusive breastfeeding. MO, months.

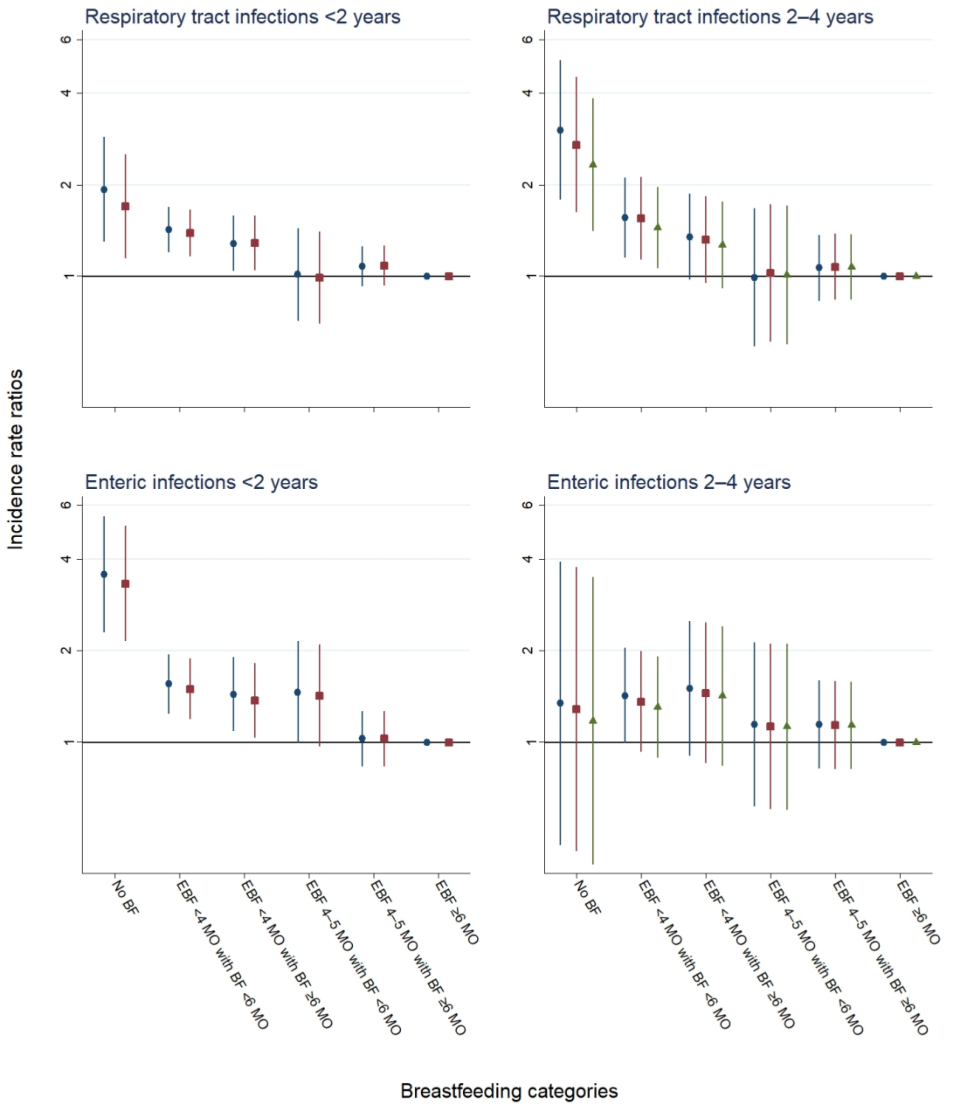

rude analyses (blue circles), adjusted analyses (red squares) and adjusted analyses controlled for previous admissions (green triangles) of the associations between breastfeeding categories, and hospitalisations for respiratory tract and enteric infections, in early childhood (<2 years) and later childhood (2–4 years). Incidence rate ratios are presented on a logarithmic scale. Vertical lines represent 95% confidence intervals around the point estimates. BF, breastfeeding. EBF exclusive breastfeeding. MO, months

Appendix A

Table A: Infectious disease categories and associated ICD-10 codes

Category	Group	ICD codes
Enteric infections	Enteric infections	A00, A01, A020, A029, A03, A04, A05, A06, A07,A08
	Enteric symptoms	A09, I880, K528, K529, R11
Upper respiratory tract infections	Upper RTI	J00, J01, J02, J03, J04, J05, J06, J32, J340, J36, J37, J390, J391
	Ear infections	H600, H601, H602, H603, H608, H609, H62, H65, H66, H67, H680, H70, H730, H750, H830, H940
Lower respiratory tract infections	Acute lower RTI	A481, A482, B59, J09, J10, J11, J12, J13, J14, J15, J16, J17, J18, J20, J21, J22
	Chronic lower RTI	J40, J41, J42, J440, J47, J85, J86, J988
	Tuberculosis	A15, A16, A17, A18, A19, N740, N741, J65
Other infections	Sepsis	A391, A392, A393, A394, A395, A398, A399 A40, A41, A483, A021, A327
	HIV/AIDS	B20, B21. B22, B23, B24
	STI	A50, A51, A52, A53, A54, A55, A56, A57, A58, A59, A60, A63, A64, N290
	CNS viral infections	A801, A802, A803, A804, A809, A811, A812, A818, A819, A82, A83, A84, A85, A86, A87, A88, A89
	CNS general infections	G00, G01, G02, G030, G039, G04, G05, G06, G07, G08, G09, G610, A321, A390
	Eye infections	B30, H000, H03, H043, H050, H100, H102, H103, H109, H130, H131, H160, H190, H191, H192, H220, H440, H451
	Oral infections	K02, K044, K046, K050, K052, K053, K113, K122
	Heart and Circulatory infections	B332, I00, I01, I02, I05, I06, I07, I08, I09, I301, I33, I38, I39, I400, I410, I411, I412, I430, I716, I790, I791
	Gastrointestinal tract infections	K230, K231, K25, K26, K27, K28, K293, K294, K295, K35, K36, K37, K61, K630, K632, K650, K678, K908, K930
	Hepatic infections	K750, K770, K830
	Viral Hepatitis	B15, B16, B17, B18, B19

For peer review only

Kidney infections	N00, N05, N10, N136, N151
Urinary tract infections	N300, N341, N351, N37, N390
Reproductive system infections, male	N410, N411, N412, N413, N431, N45, N410, N411, N412, N413, N431, N45, N481, N482, N490, N49, N51
Reproductive system infections, female	N70, N71, N72, N73, N74, N751, N764, N87
Skin infections, typical	A46, L00, L01, L02, L03, L04, L050, L08
Skin infections, other	B86, T009, T633, T634, T793
Breast infections	N61
Osteomyelitis	M462, M463, M464, M465
Joint infections	M00, M01
Connective tissue infections	M021, M023, M03, M600, M630, M631, M632, M650, M651, M680, M710, M711, M896
Neoplasms from infection	C11, C161, C162, C163, C164, C165, C166, C168, C169, C210, C211, C220, C46, C53, D002, D013, D06
Postoperative infections	T802, T814, T826, T827, T835, T836, T845, T846, T847, T857, T874
Adverse effect of ID treatment	R761, R762, T36, T37, T485, T487, T490, T495, T496, T499, T788, T789, T880, T881, T887
Other Bacterial infections	A20, A21, A22, A23, A24, A25, A26, A27, A28, A30, A31, A320, A328, A329, A33, A34, A35, A36, A37, A38, A42, A43, A44, A480, A484, A488, A49, A65, A66, A67, A68, A69, A70, A71, A74, A75, A77, A78, A79, B95, B96
Other Viral infections	A90, A91, A92, A93, A94, A95, A96, A98, A99, B00, B01, B02, B03, B04, B05, B06, B07, A08, B09, B25, B26, B27, B33, B34, B97
Other Mycoses	B35, B36, B37, B38, B39, B40, B41, B42, B43, B44, B45, B46, B47, B48, B49
Other Protozoan infections	B50, B51, B52, B53, B54, B55, B56, B57, B58, B60, B64
Other ID	B65, B66, B67, B68, B69, B70, B71, B72, B73, B74, B75, B76, B77, B78, B79, B80, B81, B82, B83, B85, B86, B87, B88, B89, B94, B99, E033, E321, F024, F071, I88, T64
Perinatal infections	P002, P027, P23, P35, P36, P37, P38, P39

ICD-10, International Classification of Disease, Tenth Revision. RTI, respiratory tract infections. CNS, central nervous system. ID, infectious diseases.

For peer review only

Appendix B

Table B: Regression results for all infections, early childhood (<2 years)

	Crude[*](95% CI)		Adjusted[†](95% CI)		1998-2003[‡](95% CI)		2004-2010[§](95% CI)		BMI[¶](95% CI)	
Breastfeeding:										
No BF	2.13	(1.61–2.81)	1.89	(1.45–2.47)	2.25	(1.51–3.35)	1.64	(1.17–2.32)	1.81	(1.35–2.42)
EBF <4 MO with BF <6 MO	1.46	(1.30–1.64)	1.41	(1.25–1.59)	1.34	(1.13–1.59)	1.48	(1.25–1.74)	1.40	(1.23–1.59)
EBF <4 MO with BF ≥6 MO	1.33	(1.16–1.52)	1.31	(1.14–1.50)	1.26	(1.02–1.56)	1.35	(1.12–1.63)	1.29	(1.11–1.49)
EBF 4–5 MO with BF <6 MO	1.19	(0.95–1.50)	1.16	(0.92–1.46)	1.21	(0.86–1.71)	1.11	(0.84–1.47)	1.09	(0.84–1.40)
EBF 4–5 MO with BF ≥6 MO	1.04	(0.94–1.15)	1.05	(0.94–1.16)	1.06	(0.92–1.23)	1.04	(0.89–1.20)	1.04	(0.93–1.16)
EBF ≥6 MO	1	ref	1	ref	1	ref	1	ref	1	ref
SGA:										
Yes			1.58	(1.14–2.19)	1.59	(0.98–2.57)	1.55	(0.99–2.43)	1.60	(1.12–2.28)
No			1	ref	1	ref	1	ref	1	ref
LGA:										
Yes			1.22	(1.02–1.47)	1.40	(1.06–1.84)	1.08	(0.86–1.36)	1.21	(0.99–1.47)
No			1	ref	1	ref	1	ref	1	ref
Congenital malformation:										
Yes			2.25	(1.82–2.79)	2.18	(1.64–2.90)	2.32	(1.71–3.17)	2.27	(1.82–2.84)
No			1	ref	1	ref	1	ref	1	ref
Sex:										
Male			1	ref	1	ref	1	ref	1	ref
Female			0.89	(0.82–0.96)	0.95	(0.85–1.07)	0.83	(0.75–0.93)	0.88	(0.81–0.96)
Maternal age (years):										
≤19			1.00	(0.71–1.42)	1.03	(0.65–1.63)	0	(0.59–1.63)	1.08	(0.75–1.55)
20-24			1	ref	1	ref	1	ref	1	ref
25-29			1.00	(0.86–1.15)	1.06	(0.85–1.32)	0.96	(0.79–1.17)	1.01	(0.86–1.18)
30-34			0.95	(0.82–1.10)	1.01	(0.80–1.28)	0.91	(0.75–1.11)	0.95	(0.81–1.11)
≥35			0.88	(0.74–1.05)	1.01	(0.77–1.32)	0.80	(0.64–1.00)	0.89	(0.74–1.07)
Parity:										
1			1	ref	1	ref	1	ref	1	ref
2			1.18	(1.07–1.29)	1.06	(0.93–1.22)	1.29	(1.14–1.46)	1.17	(1.06–1.30)
3			1.17	(1.03–1.33)	1.04	(0.86–1.25)	1.30	(1.10–1.55)	1.17	(1.03–1.34)
≥4			1.42	(1.18–1.71)	1.23	(0.95–1.61)	1.62	(1.25–2.10)	1.47	(1.21–1.79)

For peer review only

Maternal smoking:										
No smoking			1	ref	1	ref	1	ref	1	ref
Smoking			1.04	(0.89–1.21)	0.99	(0.80–1.23)	1.10	(0.89–1.37)	1.04	(0.89–1.22)
Maternal education (years):										
≤9			1.07	(0.91–1.25)	1.09	(0.87–1.37)	1.03	(0.83–1.29)	1.05	(0.89–1.24)
10-12			1.03	(0.93–1.13)	1.01	(0.86–1.17)	1.04	(0.91–1.18)	1.04	(0.93–1.16)
13-14			1.01	(0.88–1.15)	1.00	(0.83–1.21)	1.00	(0.83–1.21)	1.02	(0.88–1.17)
≥15			1	ref	1	ref	1	ref	1	ref
Maternal country of birth:										
Sweden			1	ref	1	ref	1	ref	1	ref
Other Nordic			1.06	(0.82–1.37)	1.09	(0.78–1.54)	1.03	(0.70–1.52)	1.07	(0.82–1.40)
Europe and North America			1.04	(0.86–1.25)	1.15	(0.87–1.51)	0.96	(0.74–1.25)	1.08	(0.88–1.33)
Asia			0.94	(0.79–1.12)	0.88	(0.67–1.15)	0.98	(0.79–1.23)	0.93	(0.77–1.13)
Africa			1.41	(1.08–1.84)	1.35	(0.93–1.94)	1.45	(1.01–2.10)	1.48	(1.12–1.95)
Other			0.92	(0.58–1.45)	1.00	(0.48–2.10)	0.84	(0.49–1.45)	0.84	(0.54–1.30)
Year of birth:										
1998	1	ref	1	ref	1	ref			1	ref
1999	0.80	(0.64–0.99)	0.81	(0.66–1.01)	0.81	(0.66–1.00)			0.79	(0.63–0.99)
2000	0.99	(0.82–1.21)	1.02	(0.84–1.24)	1.01	(0.83–1.22)			1.03	(0.84–1.26)
2001	0.89	(0.74–1.09)	0.93	(0.77–1.13)	0.93	(0.76–1.12)			0.94	(0.76–1.16)
2002	0.87	(0.72–1.06)	0.90	(0.74–1.09)	0.89	(0.73–1.08)			0.92	(0.75–1.12)
2003	0.79	(0.65–0.96)	0.82	(0.68–0.99)	0.81	(0.67–0.98)			0.78	(0.63–0.95)
2004	0.84	(0.70–1.02)	0.89	(0.73–1.07)			1	ref	0.94	(0.77–1.15)
2005	0.84	(0.70–1.02)	0.88	(0.73–1.06)			0.99	(0.83–1.20)	0.90	(0.74–1.10)
2006	0.73	(0.60–0.89)	0.77	(0.64–0.93)			0.87	(0.73–1.05)	0.76	(0.62–0.93)
2007	0.69	(0.57–0.84)	0.73	(0.60–0.88)			0.82	(0.68–0.99)	0.71	(0.58–0.87)
2008	0.58	(0.47–0.71)	0.61	(0.50–0.75)			0.69	(0.57–0.84)	0.61	(0.49–0.75)
2009	0.59	(0.48–0.73)	0.62	(0.51–0.77)			0.70	(0.57–0.87)	0.62	(0.50–0.77)
2010	0.55	(0.44–0.67)	0.59	(0.48–0.72)			0.66	(0.54–0.81)	0.58	(0.47–0.72)
Pregnancy BMI:										
Underweight									1.09	(0.82–1.44)
Normal									1	ref
Overweight									0.98	(0.89–1.09)
Obese									1.12	(0.98–1.28)
Observations (N):	37,825		37,825		15,553		22,272		33,422	

For peer review only

Clusters (Maternal ID):	25,487	25,487	12,445	16,998	23,605

*Adjusted for year of birth. Analysis included 37,825 children.

†Adjusted for year of birth small for gestational age, large for gestational age, congenital malformation, sex, maternal age, parity, maternal smoking, maternal education level and maternal country of birth. Analysis included 37,825 children.

‡Included children born 1998 to 2003. Adjusted for year of birth small for gestational age, large for gestational age, congenital malformation, sex, maternal age, parity, maternal smoking, maternal education level and maternal country of birth. Analysis excluded children born 2004 to 2010, leaving 15,553 children.

§Included children born 2004 to 2010. Adjusted for year of birth small for gestational age, large for gestational age, congenital malformation, sex, maternal age, parity, maternal smoking, maternal education level and maternal country of birth. Analysis excluded children born 1998 to 2003, leaving 22,272 children.

¶Adjusted for year of birth small for gestational age, large for gestational age, congenital malformation, sex, maternal age, parity, maternal smoking, maternal education level, maternal country of birth and pregnancy BMI. Analysis excluded children with missing data on pregnancy BMI, leaving 33,422 children.

Incidence rate ratios were estimated using negative binomial regression models. Correlation between siblings was accounted for using generalized estimating equations with robust standard errors. CI, confidence intervals. BF, breastfeeding. EBF, exclusive breastfeeding. MO, months. SGA, small for gestation age. LGA, large for gestation age. BMI, body mass index.

For peer review only

Table C: Regression results for all infections, later childhood (2–4 years)

	Crude[*]	(95% CI)	Adjusted[†]	(95% CI)	Adjusted 2[‡]	(95% CI)	1998-2003[§]	(95% CI)	2004-2010[¶]	(95% CI)	BMI[#]	(95% CI)
Breastfeeding:												
No BF	1.98	(1.27–3.09)	1.73	(1.12–2.66)	1.52	(1.00–2.33)	2.51	(1.54–4.09)	0.82	(0.37–1.82)	1.54	(0.98–2.40)
EBF <4 MO with BF <6 MO	1.60	(1.29–1.98)	1.52	(1.23–1.87)	1.43	(1.16–1.75)	1.29	(0.97–1.71)	1.54	(1.13–2.08)	1.46	(1.17–1.82)
EBF <4 MO with BF ≥6 MO	1.35	(1.07–1.71)	1.31	(1.03–1.66)	1.27	(1.00–1.61)	1.39	(1.01–1.91)	1.22	(0.86–1.73)	1.32	(1.03–1.69)
EBF 4–5 MO with BF <6 MO	0.98	(0.69–1.38)	0.97	(0.69–1.38)	0.97	(0.68–1.37)	0.90	(0.59–1.38)	1.08	(0.61–1.89)	1.01	(0.70–1.47)
EBF 4–5 MO with BF ≥6 MO	1.05	(0.88–1.24)	1.05	(0.88–1.24)	1.05	(0.88–1.24)	1.07	(0.86–1.34)	1.04	(0.81–1.35)	1.04	(0.86–1.24)
EBF ≥6 MO	1	ref	1	ref	1	ref	1	ref	1	ref	1	ref
SGA:												
Yes			2.23	(1.43–3.46)	2.12	(1.37–3.29)	1.68	(0.88–3.23)	2.49	(1.41–4.40)	2.26	(1.45–3.53)
No			1	ref	1	ref	1	ref	1	ref	1	ref
LGA:												
Yes			1.21	(0.91–1.60)	1.17	(0.89–1.54)	1.03	(0.68–1.53)	1.33	(0.92–1.93)	1.08	(0.80–1.45)
No			1	ref	1	ref	1	ref	1	ref	1	ref
Congenital malformation:												
Yes			3.44	(2.36–5.03)	2.89	(2.03–4.11)	2.37	(1.66–3.39)	3.42	(1.97–5.95)	2.96	(2.04–4.29)
No			1	ref	1	ref	1	ref	1	ref	1	ref
Sex												
Male			1	ref	1	ref	1	ref	1	ref	1	ref
Female			0.90	(0.78–1.04)	0.91	(0.79–1.05)	0.94	(0.78–1.12)	0.87	(0.71–1.07)	0.92	(0.79–1.07)
Maternal age (years):												
≤19			0.64	(0.33–1.24)	0.65	(0.33–1.26)	0.67	(0.27–1.53)	0.67	(0.24–1.88)	0.67	(0.33–1.35)
20–24			1	ref	1	ref	1	ref	1	ref	1	ref
25–29			0.98	(0.78–1.25)	0.98	(0.77–1.23)	0.96	(0.71–1.28)	0.99	(0.70–1.39)	1.00	(0.78–1.27)
30–34			0.82	(0.65–1.04)	0.82	(0.65–1.04)	1.02	(0.75–1.39)	0.66	(0.47–0.93)	0.81	(0.63–1.04)
≥35			0.96	(0.73–1.25)	0.98	(0.75–1.27)	1.22	(0.86–1.74)	0.81	(0.56–1.18)	0.97	(0.73–1.29)
Parity:												
1			1	ref	1	ref	1	ref	1	ref	1	ref
2			0.98	(0.84–1.14)	0.96	(0.83–1.12)	0.90	(0.73–1.12)	1.04	(0.84–1.30)	0.98	(0.83–1.15)
3			0.99	(0.80–1.23)	0.97	(0.78–1.20)	0.85	(0.63–1.15)	1.11	(0.82–1.50)	0.97	(0.78–1.23)
≥4			0.99	(0.67–1.48)	0.94	(0.64–1.36)	0.60	(0.39–0.92)	1.33	(0.81–2.18)	0.96	(0.65–1.43)
Maternal smoking:												
No smoking			1	ref	1	ref	1	ref	1	ref	1	ref

For peer review only

Smoking			1.04	(0.80–1.37)	1.04	(0.80–1.34)	0.92	(0.68–1.25)	1.16	(0.78–1.73)	0.96	(0.73–1.27)
Maternal education (years):												
≤9			1.15	(0.87–1.53)	1.13	(0.86–1.49)	1.24	(0.89–1.73)	0.99	(0.65–1.53)	1.19	(0.89–1.57)
10–12			0.90	(0.76–1.07)	0.90	(0.76–1.07)	0.98	(0.77–1.26)	0.81	(0.64–1.03)	0.91	(0.75–1.09)
13–14			1.11	(0.91–1.37)	1.12	(0.92–1.37)	1.10	(0.82–1.46)	1.13	(0.84–1.51)	1.10	(0.89–1.37)
≥15			1	ref	1	ref	1	ref	1	ref	1	ref
Maternal country of birth:												
Sweden			1	ref	1	ref	1	ref	1	ref	1	ref
Other Nordic			0.91	(0.47–1.78)	0.89	(0.46–1.72)	0.66	(0.33–1.31)	1.20	(0.43–3.37)	0.90	(0.44–1.84)
Europe and North America			0.99	(0.72–1.37)	0.98	(0.71–1.35)	1.09	(0.68–1.72)	0.90	(0.58–1.38)	0.86	(0.60–1.24)
Asia			1.04	(0.77–1.40)	1.05	(0.78–1.40)	0.88	(0.60–1.27)	1.17	(0.77–1.76)	1.12	(0.82–1.51)
Africa			1.23	(0.70–2.18)	1.14	(0.66–1.97)	1.19	(0.67–2.12)	1.12	(0.48–2.61)	1.14	(0.64–2.02)
Other			1.05	(0.54–2.05)	1.03	(0.56–1.89)	1.33	(0.58–3.04)	0.76	(0.32–1.80)	0.90	(0.49–1.66)
Year of birth:												
1998	1	ref	1	ref	1	ref			1	ref	1	ref
1999	1.01	(0.74–1.38)	1.05	(0.74–1.38)	1.10	(0.80–1.50)	1.07	(0.79–1.46)			1.18	(0.85–1.62)
2000	0.99	(0.72–1.36)	1.04	(0.72–1.36)	1.03	(0.76–1.41)	1.02	(0.75–1.39)			1.00	(0.72–1.39)
2001	0.93	(0.67–1.29)	0.99	(0.67–1.29)	0.99	(0.72–1.36)	0.97	(0.71–1.32)			0.91	(0.64–1.30)
2002	0.97	(0.71–1.31)	1.01	(0.71–1.31)	1.03	(0.76–1.39)	1.00	(0.74–1.35)			1.05	(0.76–1.44)
2003	0.94	(0.69–1.29)	0.98	(0.69–1.29)	1.01	(0.74–1.37)	0.97	(0.72–1.33)			1.06	(0.76–1.48)
2004	0.72	(0.52–1.00)	0.77	(0.52–1.00)	0.78	(0.56–1.07)	1	ref			0.88	(0.63–1.23)
2005	0.79	(0.55–1.12)	0.83	(0.55–1.12)	0.83	(0.59–1.17)			1.07	(0.76–1.50)	0.77	(0.54–1.11)
2006	0.56	(0.40–0.80)	0.60	(0.40–0.80)	0.62	(0.44–0.87)			0.80	(0.57–1.12)	0.62	(0.43–0.90)
2007	0.70	(0.50–1.00)	0.74	(0.50–1.00)	0.78	(0.55–1.10)			1.01	(0.72–1.44)	0.79	(0.55–1.14)
2008	0.66	(0.46–0.95)	0.71	(0.46–0.95)	0.76	(0.53–1.09)			1.00	(0.70–1.43)	0.79	(0.54–1.15)
2009	0.68	(0.46–1.02)	0.72	(0.46–1.02)	0.76	(0.52–1.13)			0.98	(0.68–1.42)	0.77	(0.52–1.16)
2010	0.53	(0.38–0.74)	0.58	(0.38–0.74)	0.63	(0.45–0.87)			0.83	(0.59–1.15)	0.65	(0.46–0.91)
Previous admissions:												
Yes					3.39	(2.78–4.12)	2.86	(2.24–3.65)	3.94	(2.94–5.28)	3.46	(2.81–4.26)
No					1	ref	1	ref	1	ref	1	ref
Pregnancy BMI:												
Underweight											0.74	(0.45–1.22)
Normal											1	ref
Overweight											1.01	(0.84–1.20)
Obese											1.03	(0.83–1.29)

For peer review only

Observations (N):	37,621	37,621	37,621	15,466	22,155	33,236
Clusters (Maternal ID):	25,344	25,344	25,344	12,377	16,910	23,471

*Adjusted for year of birth. Analysis included 37,621 children.

†Adjusted for year of birth small for gestational age, large for gestational age, congenital malformation, sex, maternal age, parity, maternal smoking, maternal education level and maternal country of birth. Analysis included 37,621 children.

‡Adjusted for year of birth small for gestational age, large for gestational age, congenital malformation, sex, maternal age, parity, maternal smoking, maternal education level, maternal country of birth and previous admissions. Analysis included 37,621 children.

§Included children born 1998 to 2003. Adjusted for year of birth small for gestational age, large for gestational age, congenital malformation, sex, maternal age, parity, maternal smoking, maternal education level, maternal country of birth and previous admissions. Analysis excluded children born 2004 to 2010, leaving 15,466 children.

¶Included children born 2004 to 2010. Adjusted for year of birth small for gestational age, large for gestational age, congenital malformation, sex, maternal age, parity, maternal smoking, maternal education level, maternal country of birth and previous admissions. Analysis excluded children born 1998 to 2003, leaving 22,155 children.

#Adjusted for year of birth small for gestational age, large for gestational age, congenital malformation, sex, maternal age, parity, maternal smoking, maternal education level, maternal country of birth, previous admissions and pregnancy BMI. Analysis excluded children with missing data on pregnancy BMI, leaving 33,236 children.

Incidence rate ratios were estimated using negative binomial regression models. Correlation between siblings was accounted for using generalized estimating equations with robust standard errors. BF, breastfeeding. EBF exclusive breastfeeding. MO, months. SGA, small for gestation age. LGA, large for gestation age. BMI, body mass index.

For peer review only

Appendix C

Table D: Regression results for respiratory infections, early childhood (<2 years)

	Crude[*]	(95% CI)	Adjusted[†]	(95% CI)	1998-2003[‡]	(95% CI)	2004-2010[§]	(95% CI)	BMI[¶]	(95% CI)
Breastfeeding :										
No BF	1.93	(1.30–2.87)	1.70	(1.15–2.52)	2.18	(1.21–3.93)	1.37	(0.82–2.29)	1.69	(1.11–2.57)
EBF <4 MO with BF <6 MO	1.43	(1.20–1.69)	1.39	(1.17–1.65)	1.38	(1.07–1.78)	1.40	(1.10–1.79)	1.32	(1.10–1.60)
EBF <4 MO with BF ≥6 MO	1.28	(1.04–1.58)	1.29	(1.05–1.59)	1.29	(0.93–1.78)	1.28	(0.98–1.69)	1.21	(0.97–1.51)
EBF 4–5 MO with BF <6 MO	1.02	(0.72–1.44)	0.99	(0.70–1.40)	1.04	(0.62–1.75)	0.95	(0.61–1.49)	0.88	(0.59–1.30)
EBF 4–5 MO with BF ≥6 MO	1.08	(0.93–1.26)	1.09	(0.93–1.26)	1.09	(0.88–1.36)	1.08	(0.88–1.34)	1.04	(0.89–1.22)
EBF ≥6 MO	1	ref	1	ref	1	ref	1	ref	1	ref
SGA:										
Yes			1.85	(1.15–2.97)	1.78	(0.99–3.21)	1.89	(0.94–3.81)	1.92	(1.16–3.20)
No			1	ref	1	ref	1	ref	1	ref
LGA:										
Yes			1.29	(1.02–1.64)	1.50	(1.06–2.10)	1.14	(0.82–1.58)	1.27	(0.98–1.64)
No			1	ref	1	ref	1	ref	1	ref
Congenital malformation:										
Yes			2.35	(1.81–3.05)	2.63	(1.89–3.65)	1.97	(1.29–3.01)	2.36	(1.80–3.11)
No			1	ref	1	ref	1	ref	1	ref
Sex										
Male			1	ref	1	ref	1	ref	1	ref
Female			0.70	(0.62–0.79)	0.78	(0.65–0.93)	0.64	(0.55–0.75)	0.69	(0.61–0.78)
Maternal age (years):										
≤19			0.99	(0.55–1.79)	0.70	(0.28–1.76)	0	(0.61–2.80)	1.10	(0.60–2.04)
20-24			1	ref	1	ref	1	ref	1	ref
25-29			1.05	(0.85–1.30)	1.10	(0.80–1.53)	1.02	(0.77–1.36)	1.06	(0.84–1.34)
30-34			1.05	(0.84–1.31)	1.03	(0.73–1.45)	1.08	(0.81–1.43)	1.06	(0.84–1.35)
≥35			0.97	(0.76–1.25)	1.11	(0.75–1.65)	0.89	(0.64–1.23)	1.00	(0.76–1.31)
Parity										
1			1	ref	1	ref	1	ref	1	ref
2			1.47	(1.28–1.68)	1.31	(1.06–1.61)	1.61	(1.34–1.93)	1.44	(1.24–1.67)
3			1.37	(1.13–1.66)	1.11	(0.81–1.51)	1.63	(1.28–2.07)	1.36	(1.11–1.66)
≥4			1.63	(1.25–2.13)	1.51	(1.01–2.24)	1.75	(1.22–2.50)	1.64	(1.24–2.17)

For peer review only

Maternal smoking:										
No smoking			1	ref	1	ref	1	ref	1	ref
Smoking			1.17	(0.93–1.46)	1.18	(0.86–1.64)	1.14	(0.83–1.55)	1.15	(0.90–1.47)
Maternal education (years):										
≤9			1.00	(0.79–1.26)	0.99	(0.69–1.41)	1.01	(0.74–1.38)	0.99	(0.77–1.27)
10-12			0.98	(0.85–1.13)	0.98	(0.78–1.22)	0.99	(0.83–1.19)	0.99	(0.85–1.15)
13-14			0.93	(0.77–1.13)	0.93	(0.70–1.23)	0.94	(0.72–1.23)	0.94	(0.77–1.15)
≥15			1	ref	1	ref	1	ref	1	ref
Maternal country of birth:										
Sweden			1	ref	1	ref	1	ref	1	ref
Other Nordic			1.03	(0.72–1.48)	1.04	(0.62–1.74)	1.02	(0.61–1.71)	1.05	(0.72–1.52)
Europe and North America			0.68	(0.49–0.95)	0.72	(0.45–1.15)	0.67	(0.42–1.06)	0.75	(0.53–1.06)
Asia			0.81	(0.62–1.06)	0.73	(0.48–1.12)	0.86	(0.61–1.21)	0.84	(0.63–1.12)
Africa			1.17	(0.78–1.74)	0.87	(0.46–1.66)	1.36	(0.82–2.26)	1.25	(0.82–1.90)
Other			0.85	(0.43–1.66)	0.84	(0.24–2.93)	0.85	(0.41–1.77)	0.65	(0.33–1.28)
Year of birth:										
1998	1	ref	1	ref	1	ref			1	ref
1999	0.82	(0.60–1.13)	0.84	(0.62–1.16)	0.84	(0.61–1.15)			0.84	(0.60–1.17)
2000	0.85	(0.62–1.16)	0.88	(0.65–1.20)	0.87	(0.64–1.18)			0.95	(0.69–1.31)
2001	0.94	(0.72–1.24)	0.99	(0.75–1.30)	0.98	(0.75–1.30)			0.97	(0.72–1.30)
2002	0.87	(0.66–1.15)	0.91	(0.69–1.20)	0.90	(0.68–1.19)			0.90	(0.67–1.22)
2003	0.73	(0.55–0.97)	0.77	(0.58–1.02)	0.76	(0.57–1.01)			0.74	(0.54–1.00)
2004	0.71	(0.54–0.93)	0.75	(0.57–0.98)			1	ref	0.81	(0.61–1.08)
2005	0.91	(0.69–1.19)	0.94	(0.72–1.23)			1.26	(0.97–1.66)	1.01	(0.76–1.33)
2006	0.74	(0.56–0.97)	0.78	(0.59–1.03)			1.04	(0.79–1.38)	0.74	(0.55–1.00)
2007	0.85	(0.65–1.11)	0.90	(0.69–1.17)			1.20	(0.92–1.57)	0.89	(0.67–1.18)
2008	0.58	(0.44–0.77)	0.62	(0.47–0.83)			0.83	(0.63–1.11)	0.60	(0.44–0.81)
2009	0.71	(0.54–0.95)	0.76	(0.57–1.01)			1.02	(0.77–1.36)	0.77	(0.57–1.03)
2010	0.64	(0.48–0.85)	0.70	(0.53–0.92)			0.93	(0.70–1.24)	0.67	(0.50–0.91)
Pregancy BMI:										
Underweight									1.02	(0.67–1.56)
Normal									1	ref
Overweight									0.94	(0.81–1.09)
Obese									1.16	(0.96–1.41)
Observations (N):	37,825		37,825		15,553		22,272		33,422	

For peer review only

Clusters (Maternal ID):	25,487	25,487	12,445	16,998	23,605

*Adjusted for year of birth. Analysis included 37,825 children.

†Adjusted for year of birth small for gestational age, large for gestational age, congenital malformation, sex, maternal age, parity, maternal smoking, maternal education level and maternal country of birth. Analysis included 37,825 children.

‡Included children born 1998 to 2003. Adjusted for year of birth small for gestational age, large for gestational age, congenital malformation, sex, maternal age, parity, maternal smoking, maternal education level and maternal country of birth. Analysis excluded children born 2004 to 2010, leaving 15,553 children.

§Included children born 2004 to 2010. Adjusted for year of birth small for gestational age, large for gestational age, congenital malformation, sex, maternal age, parity, maternal smoking, maternal education level and maternal country of birth. Analysis excluded children born 1998 to 2003, leaving 22,272 children.

¶Adjusted for year of birth small for gestational age, large for gestational age, congenital malformation, sex, maternal age, parity, maternal smoking, maternal education level, maternal country of birth and pregnancy BMI. Analysis excluded children with missing data on pregnancy BMI, leaving 33,422 children.

Incidence rate ratios were estimated using negative binomial regression models. Correlation between siblings was accounted for using generalized estimating equations with robust standard errors. CI, confidence intervals. BF, breastfeeding. EBF, exclusive breastfeeding. MO, months. SGA, small for gestation age. LGA, large for gestation age. BMI, body mass index.

For peer review only

Table E: Regression results for respiratory infections, later childhood (2–4 years)

	Crude[*](95% CI)		Adjusted[†](95% CI)		Adjusted 2[‡](95% CI)		1998-2003[§](95% CI)		2004-2010[¶](95% CI)		BMI[#](95% CI)	
Breastfeeding:												
No BF	3.03	(1.78–5.13)	2.70	(1.62–4.50)	2.53	(1.51–4.24)	4.60	(2.49–8.48)	1.06	(0.41–2.76)	2.60	(1.52–4.45)
EBF <4 MO with BF <6 MO	1.56	(1.15–2.11)	1.55	(1.13–2.12)	1.47	(1.08–2.01)	1.58	(1.04–2.40)	1.37	(0.87–2.15)	1.50	(1.07–2.10)
EBF <4 MO with BF ≥6 MO	1.35	(0.98–1.87)	1.32	(0.95–1.83)	1.28	(0.92–1.77)	1.44	(0.89–2.33)	1.18	(0.77–1.83)	1.35	(0.96–1.90)
EBF 4–5 MO with BF <6 MO	0.99	(0.59–1.67)	1.03	(0.61–1.73)	1.03	(0.61–1.73)	0.86	(0.43–1.73)	1.24	(0.59–2.61)	1.11	(0.65–1.91)
EBF 4–5 MO with BF ≥6 MO	1.07	(0.83–1.37)	1.08	(0.84–1.38)	1.07	(0.84–1.38)	1.10	(0.78–1.56)	1.04	(0.73–1.48)	1.08	(0.83–1.40)
EBF ≥6 MO	1	ref	1	ref	1	ref	1	ref	1	ref	1	ref
SGA												
Yes			2.26	(1.26–4.04)	2.26	(1.25–4.07)	1.45	(0.57–3.68)	3.10	(1.50–6.43)	2.44	(1.32–4.50)
No			1	ref	1	ref	1	ref	1	ref	1	ref
LGA												
Yes			1.17	(0.79–1.74)	1.16	(0.78–1.72)	1.05	(0.57–1.92)	1.24	(0.75–2.05)	1.15	(0.76–1.75)
No			1	ref	1	ref	1	ref	1	ref	1	ref
Congenital malformation:												
Yes			3.75	(2.59–5.43)	3.22	(2.24–4.62)	2.82	(1.71–4.66)	3.44	(2.07–5.73)	3.11	(2.11–4.58)
No			1	ref	1	ref	1	ref	1	ref	1	ref
Sex												
Male			1	ref	1	ref	1	ref	1	ref	1	ref
Female			0.79	(0.65–0.95)	0.82	(0.67–0.99)	0.91	(0.69–1.19)	0.73	(0.56–0.95)	0.84	(0.68–1.02)
Maternal age (years):												
≤19			0.58	(0.21–1.61)	0.61	(0.22–1.69)	0.72	(0.13–2.29)	0.70	(0.16–3.06)	0.72	(0.25–2.04)
20-24			1	ref	1	ref	1	ref	1	ref	1	ref
25-29			1.19	(0.85–1.66)	1.19	(0.85–1.66)	1.17	(0.72–1.88)	1.20	(0.75–1.91)	1.24	(0.87–1.78)
30-34			1.06	(0.76–1.48)	1.06	(0.76–1.48)	1.51	(0.94–2.45)	0.75	(0.47–1.19)	1.14	(0.79–1.63)
≥35			1.39	(0.95–2.03)	1.39	(0.95–2.03)	1.88	(1.10–3.22)	1.05	(0.62–1.77)	1.46	(0.97–2.19)
Parity:												
1			1	ref	1	ref	1	ref	1	ref	1	ref
2			1.04	(0.83–1.29)	1.01	(0.81–1.25)	0.85	(0.62–1.16)	1.22	(0.91–1.64)	1.01	(0.80–1.27)
3			0.88	(0.63–1.23)	0.86	(0.62–1.19)	0.56	(0.35–0.92)	1.25	(0.81–1.93)	0.83	(0.58–1.19)
≥4			0.92	(0.60–1.41)	0.88	(0.58–1.36)	0.62	(0.35–1.12)	1.24	(0.67–2.30)	0.94	(0.61–1.47)
Maternal smoking:												

For peer review only

No smoking			1	ref	1	ref	1	ref	1	ref	1	ref
Smoking			0.91	(0.64–1.31)	0.89	(0.63–1.28)	0.93	(0.61–1.44)	0.84	(0.45–1.54)	0.78	(0.52–1.15)
Maternal education (years):												
≤9			1.12	(0.77–1.63)	1.11	(0.76–1.61)	1.36	(0.85–2.17)	0.90	(0.49–1.66)	1.18	(0.80–1.73)
10-12			0.96	(0.76–1.21)	0.96	(0.76–1.22)	1.15	(0.82–1.64)	0.82	(0.60–1.13)	1.02	(0.79–1.31)
13-14			1.13	(0.84–1.51)	1.14	(0.86–1.52)	1.05	(0.69–1.59)	1.28	(0.87–1.90)	1.20	(0.88–1.62)
≥15			1	ref	1	ref	1	ref	1	ref	1	ref
Maternal country of birth:												
Sweden			1	ref	1	ref	1	ref	1	ref	1	ref
Other Nordic			0.82	(0.44–1.52)	0.80	(0.43–1.48)	0.80	(0.33–1.91)	0.77	(0.32–1.86)	0.78	(0.40–1.50)
Europe and North America			0.58	(0.33–1.03)	0.60	(0.34–1.07)	0.74	(0.32–1.75)	0.50	(0.24–1.07)	0.48	(0.25–0.89)
Asia			1.05	(0.67–1.62)	1.08	(0.70–1.68)	1.05	(0.62–1.78)	1.04	(0.54–1.99)	1.15	(0.75–1.78)
Africa			1.15	(0.46–2.85)	1.10	(0.44–2.77)	1.21	(0.53–2.79)	1.00	(0.23–4.32)	1.10	(0.41–2.98)
Other			1.48	(0.58–3.80)	1.38	(0.59–3.20)	2.25	(0.89–5.65)	0.61	(0.15–2.41)	1.00	(0.42–2.40)
Year of birth:												
1998	1	ref	1	ref	1	ref			1	ref	1	ref
1999	1.02	(0.63–1.64)	1.07	(0.63–1.64)	1.11	(0.69–1.78)	1.10	(0.68–1.76)			1.18	(0.72–1.94)
2000	1.18	(0.74–1.88)	1.25	(0.74–1.88)	1.27	(0.80–2.02)	1.25	(0.79–1.97)			1.32	(0.80–2.15)
2001	1.02	(0.63–1.66)	1.08	(0.63–1.66)	1.07	(0.67–1.71)	1.02	(0.64–1.62)			1.02	(0.61–1.71)
2002	0.85	(0.53–1.36)	0.88	(0.53–1.36)	0.89	(0.56–1.43)	0.87	(0.54–1.40)			0.84	(0.50–1.41)
2003	0.92	(0.57–1.49)	0.96	(0.57–1.49)	0.97	(0.60–1.56)	0.94	(0.58–1.52)			1.04	(0.62–1.74)
2004	0.94	(0.59–1.52)	1.00	(0.59–1.52)	1.04	(0.64–1.67)	1	ref			1.16	(0.69–1.93)
2005	0.76	(0.47–1.23)	0.79	(0.47–1.23)	0.79	(0.49–1.27)			0.76	(0.48–1.21)	0.76	(0.46–1.27)
2006	0.72	(0.43–1.22)	0.77	(0.43–1.22)	0.78	(0.47–1.31)			0.75	(0.47–1.21)	0.80	(0.46–1.40)
2007	0.86	(0.54–1.37)	0.91	(0.54–1.37)	0.92	(0.58–1.46)			0.90	(0.57–1.41)	1.00	(0.62–1.62)
2008	0.57	(0.33–0.96)	0.60	(0.33–0.96)	0.64	(0.38–1.08)			0.62	(0.38–1.03)	0.66	(0.38–1.15)
2009	0.89	(0.55–1.42)	0.96	(0.55–1.42)	0.99	(0.62–1.59)			0.98	(0.61–1.55)	0.97	(0.59–1.61)
2010	0.56	(0.34–0.91)	0.62	(0.34–0.91)	0.64	(0.40–1.05)			0.64	(0.40–1.03)	0.69	(0.41–1.14)
Previous admissions:												
Yes					4.48	(3.28–6.11)	4.68	(3.14–6.98)	4.20	(2.63–6.71)	4.47	(3.20–6.25)
No					1	ref	1	ref	1	ref	1	ref
Pregancy BMI:												
Underweight											0.55	(0.22–1.36)
Normal											1	ref
Overweight											0.85	(0.66–1.10)

For peer review only

Obese						1.05	(0.78–1.43)
Observations (N):	37,621	37,621	37,621	15,466	22,155	33,236	
Clusters (Maternal ID):	25,344	25,344	25,344	12,377	16,910	23,471	

*Adjusted for year of birth. Analysis included 37,621 children.

†Adjusted for year of birth small for gestational age, large for gestational age, congenital malformation, sex, maternal age, parity, maternal smoking, maternal education level and maternal country of birth. Analysis included 37,621 children.

‡Adjusted for year of birth small for gestational age, large for gestational age, congenital malformation, sex, maternal age, parity, maternal smoking, maternal education level, maternal country of birth and previous admissions. Analysis included 37,621 children.

§Included children born 1998 to 2003. Adjusted for year of birth small for gestational age, large for gestational age, congenital malformation, sex, maternal age, parity, maternal smoking, maternal education level, maternal country of birth and previous admissions. Analysis excluded children born 2004 to 2010, leaving 15,466 children.

¶Included children born 2004 to 2010. Adjusted for year of birth small for gestational age, large for gestational age, congenital malformation, sex, maternal age, parity, maternal smoking, maternal education level, maternal country of birth and previous admissions. Analysis excluded children born 1998 to 2003, leaving 22,155 children.

#Adjusted for year of birth small for gestational age, large for gestational age, congenital malformation, sex, maternal age, parity, maternal smoking, maternal education level, maternal country of birth, previous admissions and pregnancy BMI. Analysis excluded children with missing data on pregnancy BMI, leaving 33,236 children.

Incidence rate ratios were estimated using negative binomial regression models. Correlation between siblings was accounted for using generalized estimating equations with robust standard errors. CI, confidence intervals. BF, breastfeeding. EBF, exclusive breastfeeding. MO, months. SGA, small for gestation age. LGA, large for gestation age. BMI, body mass index.

For peer review only

Table F: Regression results for enteric infections, early childhood (<2 years)

	Crude[*](95% CI)		Adjusted[†](95% CI)		1998-2003[‡](95% CI)		2004-2010[§](95% CI)		BMI[¶](95% CI)	
Breastfeeding :										
No BF	3.56	(2.30–5.52)	3.32	(2.14–5.14)	3.54	(1.85–6.78)	3.34	(1.85–6.01)	3.17	(1.99–5.05)
EBF <4 MO with BF <6 MO	1.55	(1.24–1.94)	1.50	(1.19–1.89)	1.50	(1.09–2.07)	1.56	(1.11–2.20)	1.59	(1.24–2.05)
EBF <4 MO with BF ≥6 MO	1.44	(1.09–1.91)	1.37	(1.03–1.82)	1.26	(0.82–1.94)	1.52	(1.03–2.25)	1.47	(1.09–1.98)
EBF 4–5 MO with BF <6 MO	1.46	(0.99–2.15)	1.43	(0.97–2.10)	0.99	(0.53–1.86)	2.01	(1.22–3.32)	1.38	(0.89–2.13)
EBF 4–5 MO with BF ≥6 MO	1.03	(0.83–1.27)	1.03	(0.83–1.27)	1.07	(0.81–1.40)	1.03	(0.74–1.43)	1.07	(0.85–1.34)
EBF ≥6 MO	1	ref	1	ref	1	ref	1	ref	1	ref
SGA:										
Yes			1.47	(0.90–2.40)	1.34	(0.63–2.83)	1.58	(0.82–3.03)	1.69	(1.04–2.76)
No			1	ref	1	ref	1	ref	1	ref
LGA:										
Yes			1.01	(0.70–1.44)	1.15	(0.71–1.86)	0.87	(0.51–1.46)	0.91	(0.61–1.35)
No			1	ref	1	ref	1	ref	1	ref
Congenital malformation:										
Yes			2.16	(1.47–3.17)	1.70	(1.02–2.84)	2.87	(1.66–4.95)	2.30	(1.54–3.41)
No			1	ref	1	ref	1	ref	1	ref
Sex										
Male			1	ref	1	ref	1	ref	1	ref
Female			1.05	(0.90–1.23)	0.95	(0.76–1.18)	1.15	(0.92–1.44)	1.02	(0.86–1.20)
Maternal age (years):										
≤19			0.64	(0.29–1.41)	0.76	(0.26–2.18)	0	(0.17–1.75)	0.68	(0.29–1.59)
20-24			1	ref	1	ref	1	ref	1	ref
25-29			1.04	(0.79–1.36)	1.34	(0.93–1.93)	0.83	(0.56–1.22)	1.02	(0.76–1.36)
30-34			0.89	(0.67–1.19)	1.11	(0.74–1.65)	0.75	(0.50–1.12)	0.87	(0.64–1.18)
≥35			0.84	(0.60–1.17)	1.10	(0.69–1.76)	0.68	(0.44–1.08)	0.82	(0.57–1.17)
Parity										
1			1	ref	1	ref	1	ref	1	ref
2			0.89	(0.74–1.07)	0.77	(0.59–1.00)	1.03	(0.79–1.33)	0.91	(0.75–1.11)
3			0.95	(0.73–1.22)	0.91	(0.63–1.29)	0.98	(0.68–1.41)	0.99	(0.75–1.29)
≥4			1.20	(0.81–1.79)	0.80	(0.47–1.35)	1.65	(0.96–2.84)	1.37	(0.91–2.08)
Maternal smoking:										

For peer review only

No smoking			1	ref	1	ref	1	ref	1	ref
Smoking			0.97	(0.71–1.32)	0.60	(0.37–0.97)	1.46	(0.98–2.17)	1.04	(0.76–1.43)
Maternal education (years):										
≤9			1.13	(0.84–1.53)	1.28	(0.84–1.98)	0.99	(0.65–1.50)	1.03	(0.74–1.43)
10-12			0.98	(0.80–1.19)	0.94	(0.71–1.25)	0.96	(0.73–1.27)	0.99	(0.79–1.23)
13-14			0.98	(0.75–1.29)	0.88	(0.61–1.28)	1.02	(0.70–1.51)	0.94	(0.70–1.27)
≥15			1	ref	1	ref	1	ref	1	ref
Maternal country of birth:										
Sweden			1	ref	1	ref	1	ref	1	ref
Other Nordic			1.11	(0.67–1.82)	0.99	(0.49–2.00)	1.24	(0.61–2.52)	1.09	(0.64–1.85)
Europe and North America			1.40	(0.99–1.99)	0.97	(0.56–1.68)	1.82	(1.16–2.85)	1.48	(1.02–2.14)
Asia			1.28	(0.95–1.71)	0.87	(0.54–1.41)	1.63	(1.12–2.37)	1.29	(0.94–1.78)
Africa			2.13	(1.34–3.38)	2.26	(1.22–4.18)	2.10	(1.11–3.96)	2.08	(1.28–3.38)
Other			1.15	(0.58–2.29)	0.90	(0.29–2.77)	1.35	(0.56–3.22)	1.01	(0.45–2.23)
Year of birth:										
1998	1	ref	1	ref	1	ref			1	ref
1999	0.99	(0.67–1.47)	1.01	(0.68–1.48)	1.01	(0.68–1.49)			0.85	(0.56–1.30)
2000	1.30	(0.90–1.86)	1.32	(0.92–1.89)	1.31	(0.91–1.89)			1.27	(0.87–1.84)
2001	0.83	(0.56–1.23)	0.86	(0.57–1.28)	0.85	(0.57–1.27)			0.80	(0.53–1.22)
2002	0.77	(0.52–1.14)	0.78	(0.53–1.16)	0.77	(0.52–1.14)			0.77	(0.51–1.15)
2003	1.13	(0.78–1.62)	1.14	(0.79–1.64)	1.11	(0.77–1.61)			0.89	(0.60–1.33)
2004	1.36	(0.94–1.95)	1.41	(0.98–2.03)			1	ref	1.42	(0.97–2.08)
2005	0.95	(0.65–1.38)	0.98	(0.67–1.44)			0.70	(0.49–1.00)	0.91	(0.61–1.35)
2006	0.68	(0.46–1.02)	0.71	(0.47–1.06)			0.51	(0.35–0.74)	0.67	(0.44–1.01)
2007	0.64	(0.43–0.97)	0.66	(0.44–1.00)			0.47	(0.32–0.70)	0.66	(0.44–1.01)
2008	0.53	(0.35–0.80)	0.54	(0.36–0.83)			0.39	(0.26–0.57)	0.54	(0.35–0.82)
2009	0.59	(0.38–0.90)	0.60	(0.39–0.92)			0.42	(0.28–0.64)	0.57	(0.37–0.89)
2010	0.45	(0.28–0.71)	0.46	(0.29–0.74)			0.33	(0.21–0.51)	0.45	(0.28–0.71)
Pregancy BMI:										
Underweight									0.88	(0.52–1.48)
Normal									1	ref
Overweight									0.85	(0.68–1.05)
Obese									1.10	(0.85–1.42)
Observations (N):	37,825		37,825		15,553		22,272		33,422	
Clusters (Maternal ID):	25,487		25,487		12,445		16,998		23,605	

For peer review only - http://bmjopen.bmj.com/site/about/guidelines.xhtml

*Adjusted for year of birth. Analysis included 37,825 children.

†Adjusted for year of birth small for gestational age, large for gestational age, congenital malformation, sex, maternal age, parity, maternal smoking, maternal education level and maternal country of birth. Analysis included 37,825 children.

‡Included children born 1998 to 2003. Adjusted for year of birth small for gestational age, large for gestational age, congenital malformation, sex, maternal age, parity, maternal smoking, maternal education level and maternal country of birth. Analysis excluded children born 2004 to 2010, leaving 15,553 children.

§Included children born 2004 to 2010. Adjusted for year of birth small for gestational age, large for gestational age, congenital malformation, sex, maternal age, parity, maternal smoking, maternal education level and maternal country of birth. Analysis excluded children born 1998 to 2003, leaving 22,272 children.

¶Adjusted for year of birth small for gestational age, large for gestational age, congenital malformation, sex, maternal age, parity, maternal smoking, maternal education level, maternal country of birth and pregnancy BMI. Analysis excluded children with missing data on pregnancy BMI, leaving 33,422 children.

Incidence rate ratios were estimated using negative binomial regression models. Correlation between siblings was accounted for using generalized estimating equations with robust standard errors. CI, confidence intervals. BF, breastfeeding. EBF, exclusive breastfeeding. MO, months. SGA, small for gestation age. LGA, large for gestation age. BMI, body mass index.

For peer review only

Table G: Regression results for enteric infections, later childhood (2–4 years)

	Crude* (95% CI)		Adjusted† (95% CI)		Adjusted 2‡ (95% CI)		1998-2003§ (95% CI)		2004-2010¶ (95% CI)		BMI# (95% CI)	
Breastfeeding:												
No BF	1.34	(0.46–3.91)	1.28	(0.44–3.75)	1.20	(0.41–3.55)	1.91	(0.64–5.73)	-	-	1.06	(0.30–3.70)
EBF <4 MO with BF <6 MO	1.42	(0.99–2.04)	1.36	(0.93–1.99)	1.35	(0.92–1.97)	1.00	(0.61–1.64)	2.77	(1.34–5.73)	1.46	(0.98–2.18)
EBF <4 MO with BF ≥6 MO	1.50	(0.90–2.50)	1.45	(0.86–2.47)	1.45	(0.85–2.47)	1.15	(0.65–2.03)	2.82	(1.02–7.84)	1.51	(0.86–2.66)
EBF 4–5 MO with BF <6 MO	1.15	(0.62–2.13)	1.13	(0.60–2.10)	1.12	(0.60–2.09)	0.98	(0.46–2.09)	1.84	(0.59–5.74)	1.06	(0.53–2.10)
EBF 4–5 MO with BF ≥6 MO	1.15	(0.82–1.60)	1.14	(0.82–1.59)	1.14	(0.82–1.59)	0.96	(0.64–1.43)	2.08	(1.07–4.04)	1.17	(0.82–1.67)
EBF ≥6 MO	1	ref	1	ref	1	ref	1	ref	1	ref	1	ref
SGA												
Yes			2.73	(1.11–6.70)	2.67	(1.08–6.60)	3.17	(0.94–10.60)	1.93	(0.55–6.79)	2.85	(1.16–6.97)
No			1	ref	1	ref	1	ref	1	ref	1	ref
LGA												
Yes			1.06	(0.61–1.82)	1.06	(0.61–1.82)	0.81	(0.38–1.73)	1.51	(0.69–3.29)	1.03	(0.58–1.81)
No			1	ref	1	ref	1	ref	1	ref	1	ref
Congenital malformation:												
Yes			1.97	(1.04–3.71)	1.93	(1.02–3.65)	2.01	(1.00–4.05)	1.56	(0.36–6.83)	2.06	(1.09–3.90)
No			1	ref	1	ref	1	ref	1	ref	1	ref
Sex												
Male			1	ref	1	ref	1	ref	1	ref	1	ref
Female			0.95	(0.73–1.23)	0.95	(0.73–1.23)	0.85	(0.61–1.17)	1.11	(0.73–1.70)	0.92	(0.70–1.20)
Maternal age (years):												
≤19			0.52	(0.15–1.75)	0.53	(0.16–1.77)	0.57	(0.11–2.20)	0.61	(0.08–4.61)	0.57	(0.17–1.95)
20-24			1	ref	1	ref	1	ref	1	ref	1	ref
25-29			0.73	(0.50–1.07)	0.73	(0.50–1.07)	0.61	(0.38–0.98)	1.01	(0.54–1.89)	0.74	(0.49–1.09)
30-34			0.63	(0.41–0.96)	0.63	(0.41–0.97)	0.56	(0.32–0.96)	0.79	(0.40–1.54)	0.62	(0.39–0.96)
≥35			0.56	(0.35–0.92)	0.57	(0.35–0.93)	0.62	(0.33–1.14)	0.53	(0.24–1.14)	0.58	(0.35–0.96)
Parity:												
1			1	ref	1	ref	1	ref	1	ref	1	ref
2			1.05	(0.76–1.45)	1.05	(0.76–1.45)	1.05	(0.70–1.58)	1.05	(0.63–1.76)	1.05	(0.75–1.47)
3			1.07	(0.71–1.61)	1.07	(0.71–1.61)	1.07	(0.62–1.86)	1.11	(0.61–2.00)	1.05	(0.68–1.60)
≥4			1.04	(0.57–1.91)	1.04	(0.57–1.90)	0.83	(0.38–1.84)	1.42	(0.59–3.46)	0.95	(0.50–1.82)
Maternal smoking:												

For peer review only

No smoking			1	ref	1	ref	1	ref	1	ref	1	ref
Smoking			1.04	(0.67–1.61)	1.05	(0.68–1.63)	0.66	(0.35–1.22)	2.02	(1.12–3.66)	1.07	(0.69–1.68)
Maternal education (years):												
≤9			1.05	(0.64–1.72)	1.04	(0.63–1.70)	1.08	(0.58–2.03)	0.91	(0.42–1.95)	1.00	(0.60–1.68)
10-12			0.71	(0.50–1.00)	0.71	(0.50–1.00)	0.62	(0.40–0.97)	0.85	(0.51–1.42)	0.66	(0.45–0.95)
13-14			0.78	(0.52–1.18)	0.78	(0.52–1.19)	0.72	(0.43–1.21)	0.81	(0.41–1.61)	0.71	(0.45–1.10)
≥15			1	ref	1	ref	1	ref	1	ref	1	ref
Maternal country of birth:												
Sweden			1	ref	1	ref	1	ref	1	ref	1	ref
Other Nordic			2.15	(0.71–6.47)	2.14	(0.71–6.46)	1.02	(0.30–3.46)	4.88	(1.06–22.56)	2.31	(0.76–7.04)
Europe and North America			1.68	(1.00–2.84)	1.66	(0.98–2.80)	1.96	(1.04–3.72)	1.12	(0.46–2.72)	1.56	(0.87–2.79)
Asia			0.95	(0.57–1.57)	0.94	(0.57–1.57)	0.60	(0.26–1.39)	1.43	(0.75–2.74)	1.06	(0.63–1.76)
Africa			1.95	(0.89–4.28)	1.90	(0.87–4.18)	2.08	(0.84–5.19)	1.72	(0.40–7.32)	1.89	(0.80–4.45)
Other			0.74	(0.18–3.00)	0.74	(0.18–3.01)	0.58	(0.08–4.19)	1.02	(0.14–7.40)	0.82	(0.20–3.35)
Year of birth:												
1998	1	ref	1	ref	1	ref			1	ref	1	ref
1999	0.85	(0.50–1.47)	0.85	(0.50–1.47)	0.85	(0.49–1.46)	0.85	(0.49–1.46)			0.88	(0.50–1.53)
2000	0.56	(0.32–0.96)	0.56	(0.32–0.96)	0.55	(0.32–0.95)	0.55	(0.32–0.95)			0.52	(0.29–0.92)
2001	0.60	(0.33–1.09)	0.61	(0.33–1.09)	0.62	(0.34–1.12)	0.61	(0.34–1.10)			0.58	(0.30–1.11)
2002	0.89	(0.54–1.48)	0.90	(0.54–1.48)	0.91	(0.55–1.50)	0.88	(0.53–1.45)			0.99	(0.59–1.64)
2003	0.75	(0.46–1.23)	0.75	(0.46–1.23)	0.74	(0.46–1.21)	0.73	(0.45–1.18)			0.76	(0.46–1.27)
2004	0.36	(0.20–0.64)	0.36	(0.20–0.64)	0.36	(0.20–0.64)	1	ref			0.41	(0.23–0.75)
2005	0.43	(0.24–0.75)	0.43	(0.24–0.75)	0.43	(0.24–0.76)			1.27	(0.66–2.46)	0.43	(0.24–0.77)
2006	0.36	(0.20–0.64)	0.36	(0.20–0.64)	0.37	(0.21–0.65)			1.08	(0.55–2.12)	0.35	(0.19–0.64)
2007	0.28	(0.11–0.73)	0.28	(0.11–0.73)	0.28	(0.11–0.72)			0.84	(0.31–2.30)	0.27	(0.10–0.73)
2008	0.33	(0.18–0.59)	0.32	(0.18–0.59)	0.33	(0.18–0.60)			0.98	(0.49–1.94)	0.32	(0.17–0.59)
2009	0.25	(0.13–0.50)	0.24	(0.13–0.50)	0.25	(0.12–0.50)			0.75	(0.34–1.63)	0.23	(0.11–0.48)
2010	0.27	(0.15–0.50)	0.26	(0.15–0.50)	0.27	(0.15–0.50)			0.83	(0.40–1.70)	0.23	(0.12–0.45)
Previous admissions:												
Yes					3.03	(1.83–5.01)	1.96	(0.94–4.08)	5.18	(2.56–10.49)	2.87	(1.68–4.91)
No					1	ref	1	ref	1	ref	1	ref
Pregancy BMI:												
Underweight											0.76	(0.31–1.87)
Normal											1	ref
Overweight											1.22	(0.87–1.71)

For peer review only

Obese						1.17	(0.78–1.75)
Observations (N):	37,621	37,621	37,621	15,466	22,155	33,236	
Clusters (Maternal ID):	25,344	25,344	25,344	12,377	16,910	23,471	

*Adjusted for year of birth. Analysis included 37,621 children.

†Adjusted for year of birth small for gestational age, large for gestational age, congenital malformation, sex, maternal age, parity, maternal smoking, maternal education level and maternal country of birth. Analysis included 37,621 children.

‡Adjusted for year of birth small for gestational age, large for gestational age, congenital malformation, sex, maternal age, parity, maternal smoking, maternal education level, maternal country of birth and previous admissions. Analysis included 37,621 children.

§Included children born 1998 to 2003. Adjusted for year of birth small for gestational age, large for gestational age, congenital malformation, sex, maternal age, parity, maternal smoking, maternal education level, maternal country of birth and previous admissions. Analysis excluded children born 2004 to 2010, leaving 15,466 children.

¶Included children born 2004 to 2010. Adjusted for year of birth small for gestational age, large for gestational age, congenital malformation, sex, maternal age, parity, maternal smoking, maternal education level, maternal country of birth and previous admissions. Analysis excluded children born 1998 to 2003, leaving 22,155 children.

#Adjusted for year of birth small for gestational age, large for gestational age, congenital malformation, sex, maternal age, parity, maternal smoking, maternal education level, maternal country of birth, previous admissions and pregnancy BMI. Analysis excluded children with missing data on pregnancy BMI, leaving 33,236 children.

Incidence rate ratios were estimated using negative binomial regression models. Correlation between siblings was accounted for using generalized estimating equations with robust standard errors. CI, confidence intervals. BF, breastfeeding. EBF, exclusive breastfeeding. MO, months. SGA, small for gestation age. LGA, large for gestation age. BMI, body mass index. The estimate for “No BF” in 2004-2010 is not presented due to few events.

For peer review only

Table H: Regression results for upper respiratory infections, early childhood (<2 years)

	Crude[*]	(95% CI)	Adjusted[†]	(95% CI)	1998-2003[‡]	(95% CI)	2004-2010[§]	(95% CI)	BMI[¶]	(95% CI)
Breastfeeding :										
No BF	1.48	(0.88–2.49)	1.31	(0.77–2.22)	1.19	(0.56–2.54)	1.54	(0.74–3.21)	1.24	(0.70–2.18)
EBF <4 MO with BF <6 MO	1.48	(1.19–1.84)	1.40	(1.12–1.75)	1.30	(0.97–1.74)	1.60	(1.11–2.30)	1.36	(1.07–1.74)
EBF <4 MO with BF ≥6 MO	1.22	(0.92–1.62)	1.21	(0.91–1.61)	1.21	(0.80–1.82)	1.28	(0.84–1.96)	1.15	(0.85–1.56)
EBF 4–5 MO with BF <6 MO	0.97	(0.63–1.50)	0.92	(0.60–1.42)	1.01	(0.59–1.73)	0.78	(0.37–1.65)	0.75	(0.46–1.22)
EBF 4–5 MO with BF ≥6 MO	1.18	(0.97–1.44)	1.17	(0.96–1.43)	1.07	(0.82–1.38)	1.36	(0.98–1.90)	1.13	(0.91–1.40)
EBF ≥6 MO	1	ref	1	ref	1	ref	1	ref	1	ref
SGA:										
Yes			1.72	(1.06–2.80)	1.97	(1.04–3.73)	1.47	(0.68–3.14)	1.64	(0.95–2.81)
No			1	ref	1	ref	1	ref	1	ref
LGA:										
Yes			1.31	(0.97–1.75)	1.50	(1.03–2.18)	1.09	(0.67–1.76)	1.24	(0.90–1.71)
No			1	ref	1	ref	1	ref	1	ref
Congenital malformation:										
Yes			2.45	(1.78–3.38)	2.33	(1.54–3.52)	2.63	(1.58–4.37)	2.54	(1.81–3.56)
No			1	ref	1	ref	1	ref	1	ref
Sex										
Male			1	ref	1	ref	1	ref	1	ref
Female			0.74	(0.64–0.86)	0.78	(0.63–0.96)	0.70	(0.56–0.87)	0.74	(0.63–0.87)
Maternal age (years):										
≤19			0.72	(0.33–1.58)	0.54	(0.16–1.77)	0	(0.33–2.75)	0.84	(0.38–1.85)
20-24			1	ref	1	ref	1	ref	1	ref
25-29			1.04	(0.80–1.36)	1.01	(0.70–1.44)	1.10	(0.75–1.61)	1.04	(0.79–1.37)
30-34			0.99	(0.75–1.30)	0.86	(0.58–1.26)	1.16	(0.78–1.71)	0.97	(0.72–1.30)
≥35			0.93	(0.68–1.28)	1.02	(0.65–1.58)	0.84	(0.53–1.33)	0.94	(0.67–1.31)
Parity										
1			1	ref	1	ref	1	ref	1	ref
2			1.15	(0.97–1.37)	1.22	(0.96–1.54)	1.09	(0.85–1.39)	1.15	(0.95–1.38)
3			1.01	(0.78–1.32)	0.98	(0.68–1.43)	1.07	(0.74–1.54)	1.03	(0.79–1.36)
≥4			1.08	(0.77–1.51)	1.06	(0.67–1.67)	1.11	(0.68–1.83)	1.02	(0.71–1.45)
Maternal smoking:										

For peer review only

For peer review only - http://bmjopen.bmj.com/site/about/guidelines.xhtml

No smoking			1	ref	1	ref	1	ref	1	ref
Smoking			0.95	(0.70–1.29)	1.01	(0.68–1.50)	0.86	(0.53–1.38)	0.95	(0.69–1.31)
Maternal education (years):										
≤9			1.18	(0.87–1.60)	1.03	(0.66–1.61)	1.38	(0.92–2.06)	1.18	(0.85–1.64)
10-12			1.07	(0.89–1.29)	0.98	(0.75–1.27)	1.18	(0.91–1.53)	1.12	(0.91–1.37)
13-14			0.98	(0.76–1.26)	0.92	(0.65–1.29)	1.03	(0.71–1.50)	0.96	(0.73–1.25)
≥15			1	ref	1	ref	1	ref	1	ref
Maternal country of birth:										
Sweden			1	ref	1	ref	1	ref	1	ref
Other Nordic countries			0.94	(0.58–1.52)	0.96	(0.52–1.76)	0.89	(0.40–1.96)	1.07	(0.66–1.73)
Europe and North America			0.82	(0.54–1.23)	0.96	(0.58–1.61)	0.67	(0.34–1.32)	0.89	(0.58–1.36)
Asia			0.78	(0.55–1.10)	0.73	(0.44–1.21)	0.81	(0.51–1.30)	0.86	(0.60–1.23)
Africa			0.91	(0.52–1.61)	0.76	(0.32–1.79)	1.03	(0.49–2.20)	0.98	(0.54–1.77)
Other			0.53	(0.16–1.72)	0.50	(0.07–3.39)	0.55	(0.14–2.22)	0.46	(0.15–1.44)
Year of birth:										
1998	1	ref	1	ref	1	ref			1	ref
1999	0.71	(0.50–1.01)	0.73	(0.51–1.04)	0.72	(0.51–1.03)			0.68	(0.47–0.99)
2000	0.67	(0.46–0.97)	0.69	(0.48–1.00)	0.68	(0.47–0.99)			0.77	(0.53–1.11)
2001	0.78	(0.57–1.07)	0.83	(0.60–1.14)	0.82	(0.59–1.12)			0.80	(0.57–1.12)
2002	0.74	(0.54–1.01)	0.77	(0.56–1.06)	0.75	(0.55–1.04)			0.75	(0.53–1.05)
2003	0.54	(0.38–0.76)	0.57	(0.40–0.80)	0.56	(0.39–0.79)			0.53	(0.37–0.78)
2004	0.47	(0.33–0.67)	0.50	(0.35–0.71)			1	ref	0.54	(0.38–0.78)
2005	0.51	(0.35–0.72)	0.53	(0.37–0.76)			1.07	(0.73–1.57)	0.57	(0.39–0.82)
2006	0.54	(0.38–0.76)	0.57	(0.40–0.81)			1.13	(0.78–1.66)	0.53	(0.37–0.78)
2007	0.45	(0.31–0.63)	0.48	(0.34–0.67)			0.95	(0.65–1.39)	0.50	(0.35–0.71)
2008	0.32	(0.22–0.46)	0.34	(0.23–0.50)			0.68	(0.45–1.03)	0.32	(0.21–0.48)
2009	0.36	(0.25–0.52)	0.38	(0.26–0.56)			0.77	(0.51–1.15)	0.39	(0.27–0.58)
2010	0.29	(0.20–0.43)	0.32	(0.22–0.48)			0.66	(0.43–1.00)	0.31	(0.20–0.46)
Pregancy BMI:										
Underweight									0.80	(0.43–1.47)
Normal									1	ref
Overweight									0.92	(0.75–1.11)
Obese									1.11	(0.86–1.44)
Observations (N):	37,825		37,825		15,553		22,272		33,422	
Clusters (Maternal ID):	25,487		25,487		12,445		16,998		23,605	

For peer review only

*Adjusted for year of birth. Analysis included 37,825 children.

†Adjusted for year of birth small for gestational age, large for gestational age, congenital malformation, sex, maternal age, parity, maternal smoking, maternal education level and maternal country of birth. Analysis included 37,825 children.

‡Included children born 1998 to 2003. Adjusted for year of birth small for gestational age, large for gestational age, congenital malformation, sex, maternal age, parity, maternal smoking, maternal education level and maternal country of birth. Analysis excluded children born 2004 to 2010, leaving 15,553 children.

§Included children born 2004 to 2010. Adjusted for year of birth small for gestational age, large for gestational age, congenital malformation, sex, maternal age, parity, maternal smoking, maternal education level and maternal country of birth. Analysis excluded children born 1998 to 2003, leaving 22,272 children.

¶Adjusted for year of birth small for gestational age, large for gestational age, congenital malformation, sex, maternal age, parity, maternal smoking, maternal education level, maternal country of birth and pregnancy BMI. Analysis excluded children with missing data on pregnancy BMI, leaving 33,422 children.

Incidence rate ratios were estimated using negative binomial regression models. Correlation between siblings was accounted for using generalized estimating equations with robust standard errors. CI, confidence intervals. BF, breastfeeding. EBF, exclusive breastfeeding. MO, months. SGA, small for gestation age. LGA, large for gestation age. BMI, body mass index.

For peer review only

Table I: Regression results for lower respiratory infections, early childhood (<2 years)

	Crude*(95% CI)		Adjusted†(95% CI)		1998-2003‡(95% CI)		2004-2010§(95% CI)		BMI¶(95% CI)	
Breastfeeding :										
No BF	2.39	(1.38–4.13)	2.08	(1.21–3.59)	4.03	(1.79–9.09)	1.25	(0.64–2.45)	2.14	(1.21–3.77)
EBF <4 MO with BF <6 MO	1.36	(1.06–1.76)	1.39	(1.07–1.79)	1.57	(1.01–2.44)	1.28	(0.94–1.76)	1.29	(0.97–1.70)
EBF <4 MO with BF ≥6 MO	1.34	(0.99–1.81)	1.38	(1.02–1.86)	1.48	(0.86–2.57)	1.29	(0.90–1.84)	1.27	(0.92–1.75)
EBF 4–5 MO with BF <6 MO	1.07	(0.63–1.79)	1.09	(0.65–1.81)	1.12	(0.41–3.07)	1.07	(0.61–1.87)	1.04	(0.60–1.82)
EBF 4–5 MO with BF ≥6 MO	0.97	(0.77–1.22)	0.99	(0.79–1.25)	1.15	(0.77–1.72)	0.90	(0.69–1.19)	0.95	(0.75–1.20)
EBF ≥6 MO	1	ref	1	ref	1	ref	1	ref	1	ref
SGA:										
Yes			2.02	(0.96–4.25)	1.52	(0.42–5.57)	2.28	(0.94–5.56)	2.29	(1.09–4.84)
No			1	ref	1	ref	1	ref	1	ref
LGA:										
Yes			1.29	(0.90–1.84)	1.50	(0.83–2.72)	1.18	(0.76–1.83)	1.31	(0.90–1.91)
No			1	ref	1	ref	1	ref	1	ref
Congenital malformation:										
Yes			2.29	(1.50–3.51)	3.26	(1.92–5.54)	1.43	(0.72–2.84)	2.21	(1.40–3.48)
No			1	ref	1	ref	1	ref	1	ref
Sex										
Male			1	ref	1	ref	1	ref	1	ref
Female			0.65	(0.55–0.78)	0.78	(0.56–1.08)	0.59	(0.48–0.73)	0.64	(0.53–0.77)
Maternal age (years):										
≤19			1.48	(0.61–3.58)	1.19	(0.27–5.26)	0	(0.58–5.07)	1.56	(0.59–4.12)
20-24			1	ref	1	ref	1	ref	1	ref
25-29			1.07	(0.77–1.50)	1.38	(0.74–2.56)	0.96	(0.65–1.44)	1.12	(0.78–1.61)
30-34			1.15	(0.82–1.62)	1.52	(0.80–2.87)	1.02	(0.68–1.51)	1.21	(0.83–1.76)
≥35			1.04	(0.71–1.51)	1.40	(0.69–2.84)	0.90	(0.58–1.41)	1.10	(0.73–1.67)
Parity										
1			1	ref	1	ref	1	ref	1	ref
2			2.01	(1.62–2.49)	1.54	(1.03–2.31)	2.29	(1.79–2.94)	1.94	(1.54–2.44)
3			1.99	(1.51–2.62)	1.40	(0.82–2.41)	2.37	(1.74–3.23)	1.91	(1.42–2.56)
≥4			2.60	(1.75–3.87)	2.55	(1.32–4.89)	2.58	(1.58–4.21)	2.71	(1.80–4.08)
Maternal smoking:										

For peer review only

No smoking			1	ref	1	ref	1	ref	1	ref
Smoking			1.47	(1.07–2.02)	1.53	(0.91–2.59)	1.40	(0.94–2.08)	1.43	(1.00–2.03)
Maternal education (years):										
≤9			0.83	(0.58–1.18)	0.91	(0.50–1.63)	0.79	(0.51–1.24)	0.80	(0.55–1.17)
10-12			0.89	(0.73–1.09)	0.96	(0.64–1.45)	0.87	(0.69–1.10)	0.86	(0.69–1.07)
13-14			0.89	(0.68–1.18)	0.94	(0.58–1.51)	0.88	(0.62–1.26)	0.93	(0.70–1.25)
≥15			1	ref	1	ref	1	ref	1	ref
Maternal country of birth:										
Sweden			1	ref	1	ref	1	ref	1	ref
Other Nordic countries			1.15	(0.67–1.95)	1.18	(0.50–2.77)	1.13	(0.57–2.24)	1.03	(0.59–1.82)
Europe and North America			0.54	(0.30–0.96)	0.24	(0.06–0.97)	0.67	(0.36–1.25)	0.61	(0.34–1.08)
Asia			0.86	(0.59–1.26)	0.75	(0.36–1.57)	0.90	(0.59–1.39)	0.82	(0.53–1.26)
Africa			1.44	(0.88–2.36)	1.08	(0.39–2.97)	1.62	(0.92–2.86)	1.53	(0.91–2.55)
Other			1.20	(0.61–2.40)	1.60	(0.52–4.93)	1.08	(0.45–2.56)	0.84	(0.35–1.99)
Year of birth:										
1998	1	ref	1	ref	1	ref			1	ref
1999	1.21	(0.66–2.23)	1.26	(0.68–2.31)	1.27	(0.69–2.33)			1.44	(0.77–2.68)
2000	1.49	(0.85–2.64)	1.55	(0.88–2.73)	1.54	(0.87–2.70)			1.65	(0.90–3.02)
2001	1.53	(0.90–2.59)	1.60	(0.94–2.72)	1.59	(0.94–2.71)			1.62	(0.91–2.87)
2002	1.36	(0.80–2.33)	1.42	(0.83–2.43)	1.41	(0.82–2.41)			1.47	(0.82–2.63)
2003	1.41	(0.84–2.37)	1.50	(0.89–2.52)	1.51	(0.89–2.54)			1.49	(0.86–2.59)
2004	1.56	(0.97–2.53)	1.64	(1.02–2.65)			1	ref	1.80	(1.08–3.00)
2005	2.32	(1.45–3.72)	2.42	(1.51–3.87)			1.48	(1.01–2.16)	2.64	(1.61–4.34)
2006	1.45	(0.88–2.37)	1.52	(0.93–2.48)			0.93	(0.62–1.40)	1.51	(0.89–2.56)
2007	2.29	(1.44–3.64)	2.41	(1.52–3.83)			1.46	(1.01–2.11)	2.34	(1.42–3.86)
2008	1.54	(0.94–2.50)	1.63	(1.00–2.65)			1.00	(0.67–1.48)	1.65	(0.99–2.75)
2009	1.98	(1.22–3.22)	2.10	(1.31–3.38)			1.28	(0.87–1.89)	2.14	(1.29–3.53)
2010	1.87	(1.17–2.98)	2.03	(1.27–3.24)			1.22	(0.84–1.78)	2.03	(1.23–3.34)
Pregancy BMI:										
Underweight									1.33	(0.75–2.37)
Normal									1	ref
Overweight									0.97	(0.78–1.22)
Obese									1.22	(0.93–1.61)
Observations (N):	37,825		37,825		15,553		22,272		33,422	
Clusters (Maternal ID):	25,487		25,487		12,445		16,998		23,605	

For peer review only

*Adjusted for year of birth. Analysis included 37,825 children.

†Adjusted for year of birth small for gestational age, large for gestational age, congenital malformation, sex, maternal age, parity, maternal smoking, maternal education level and maternal country of birth. Analysis included 37,825 children.

‡Included children born 1998 to 2003. Adjusted for year of birth small for gestational age, large for gestational age, congenital malformation, sex, maternal age, parity, maternal smoking, maternal education level and maternal country of birth. Analysis excluded children born 2004 to 2010, leaving 15,553 children.

§Included children born 2004 to 2010. Adjusted for year of birth small for gestational age, large for gestational age, congenital malformation, sex, maternal age, parity, maternal smoking, maternal education level and maternal country of birth. Analysis excluded children born 1998 to 2003, leaving 22,272 children.

¶Adjusted for year of birth small for gestational age, large for gestational age, congenital malformation, sex, maternal age, parity, maternal smoking, maternal education level, maternal country of birth and pregnancy BMI. Analysis excluded children with missing data on pregnancy BMI, leaving 33,422 children.

Incidence rate ratios were estimated using negative binomial regression models. Correlation between siblings was accounted for using generalized estimating equations with robust standard errors. CI, confidence intervals. BF, breastfeeding. EBF, exclusive breastfeeding. MO, months. SGA, small for gestation age. LGA, large for gestation age. BMI, body mass index.

For peer review only

Table J: Regression results for upper respiratory infections, later childhood (2–4 years)

	Crude*	(95% CI)	Adjusted†	(95% CI)	Adjusted 2‡	(95% CI)	1998-2003§	(95% CI)	2004-2010¶	(95% CI)	BMI#	(95% CI)
Breastfeeding:												
No BF	2.50	(1.30–4.81)	2.25	(1.17–4.33)	2.25	(1.17–4.34)	4.04	(1.82–8.94)	0.93	(0.29–3.00)	2.16	(1.11–4.19)
EBF <4 MO with BF <6 MO	1.38	(0.97–1.95)	1.30	(0.91–1.86)	1.27	(0.88–1.81)	1.65	(1.01–2.68)	0.93	(0.55–1.55)	1.31	(0.89–1.94)
EBF <4 MO with BF ≥6 MO	1.47	(0.99–2.18)	1.45	(0.97–2.15)	1.42	(0.96–2.11)	1.41	(0.78–2.54)	1.35	(0.79–2.29)	1.52	(1.00–2.30)
EBF 4–5 MO with BF <6 MO	1.35	(0.76–2.41)	1.33	(0.74–2.37)	1.34	(0.75–2.41)	0.87	(0.37–2.04)	1.90	(0.86–4.19)	1.45	(0.79–2.68)
EBF 4–5 MO with BF ≥6 MO	1.22	(0.90–1.64)	1.22	(0.90–1.64)	1.21	(0.90–1.63)	1.23	(0.82–1.85)	1.15	(0.74–1.77)	1.23	(0.89–1.69)
EBF ≥6 MO	1	ref	1	ref	1	ref	1	ref	1	ref	1	ref
SGA												
Yes			1.22	(0.58–2.59)	1.19	(0.56–2.53)	1.27	(0.48–3.35)	1.13	(0.36–3.54)	1.20	(0.53–2.71)
No			1	ref	1	ref	1	ref	1	ref	1	ref
LGA												
Yes			1.33	(0.84–2.09)	1.29	(0.82–2.04)	1.19	(0.59–2.37)	1.42	(0.79–2.56)	1.27	(0.80–2.03)
No			1	ref	1	ref	1	ref	1	ref	1	ref
Congenital malformation:												
Yes			2.73	(1.72–4.35)	2.47	(1.56–3.91)	2.33	(1.26–4.28)	2.52	(1.26–5.05)	2.45	(1.49–4.03)
No			1	ref	1	ref	1	ref	1	ref	1	ref
Sex												
Male			1	ref	1	ref	1	ref	1	ref	1	ref
Female			0.67	(0.53–0.84)	0.68	(0.54–0.86)	0.76	(0.55–1.06)	0.61	(0.44–0.84)	0.70	(0.55–0.90)
Maternal age (years):												
≤19			0.38	(0.09–1.57)	0.40	(0.10–1.64)	0.49	(0.04–2.08)	0.60	(0.08–4.60)	0.49	(0.12–2.03)
20-24			1	ref	1	ref	1	ref	1	ref	1	ref
25-29			1.04	(0.71–1.51)	1.04	(0.71–1.52)	0.93	(0.54–1.59)	1.20	(0.69–2.09)	1.08	(0.72–1.63)
30-34			0.92	(0.63–1.36)	0.94	(0.63–1.39)	1.11	(0.65–1.90)	0.81	(0.46–1.43)	1.01	(0.66–1.54)
≥35			1.15	(0.74–1.77)	1.16	(0.75–1.80)	1.23	(0.68–2.22)	1.10	(0.58–2.12)	1.23	(0.77–1.96)
Parity:												
1			1	ref	1	ref	1	ref	1	ref	1	ref
2			1.04	(0.81–1.34)	1.03	(0.80–1.33)	0.93	(0.64–1.33)	1.17	(0.83–1.66)	1.07	(0.81–1.40)
3			0.81	(0.56–1.17)	0.80	(0.56–1.16)	0.57	(0.34–0.97)	1.10	(0.66–1.84)	0.79	(0.53–1.18)
≥4			0.98	(0.59–1.64)	0.98	(0.58–1.63)	0.81	(0.41–1.62)	1.17	(0.54–2.52)	1.09	(0.65–1.85)
Maternal smoking:												

For peer review only

No smoking			1	ref	1	ref	1	ref	1	ref	1	ref
Smoking			1.19	(0.79–1.78)	1.20	(0.80–1.80)	1.15	(0.69–1.89)	1.21	(0.62–2.37)	1.04	(0.66–1.62)
Maternal education (years):												
≤9			1.25	(0.84–1.87)	1.24	(0.83–1.85)	1.51	(0.87–2.61)	0.97	(0.51–1.84)	1.30	(0.85–2.00)
10-12			0.97	(0.73–1.27)	0.97	(0.73–1.27)	1.07	(0.71–1.63)	0.90	(0.62–1.30)	1.04	(0.77–1.41)
13-14			1.23	(0.87–1.72)	1.24	(0.88–1.73)	1.12	(0.68–1.85)	1.44	(0.92–2.27)	1.35	(0.94–1.92)
≥15			1	ref	1	ref	1	ref	1	ref	1	ref
Maternal country of birth:												
Sweden			1	ref	1	ref	1	ref	1	ref	1	ref
Other Nordic countries			0.39	(0.13–1.22)	0.39	(0.12–1.20)	0.47	(0.12–1.89)	0.29	(0.04–2.07)	0.42	(0.14–1.31)
Europe and North America			0.82	(0.44–1.53)	0.83	(0.45–1.55)	0.92	(0.36–2.35)	0.75	(0.33–1.71)	0.62	(0.31–1.26)
Asia			0.84	(0.53–1.34)	0.87	(0.54–1.38)	0.87	(0.46–1.64)	0.87	(0.45–1.68)	0.88	(0.55–1.42)
Africa			0.62	(0.23–1.64)	0.60	(0.23–1.58)	0.70	(0.19–2.60)	0.55	(0.13–2.24)	0.66	(0.25–1.73)
Other			1.37	(0.58–3.27)	1.39	(0.59–3.26)	2.37	(0.93–6.02)	0.54	(0.08–3.79)	1.27	(0.48–3.36)
Year of birth:												
1998	1	ref	1	ref	1	ref			1	ref	1	ref
1999	1.05	(0.61–1.80)	1.09	(0.61–1.80)	1.13	(0.66–1.92)	1.12	(0.66–1.91)			1.20	(0.68–2.10)
2000	1.15	(0.68–1.96)	1.20	(0.68–1.96)	1.23	(0.73–2.09)	1.22	(0.72–2.07)			1.28	(0.73–2.25)
2001	0.94	(0.56–1.59)	0.99	(0.56–1.59)	1.00	(0.60–1.68)	0.98	(0.59–1.64)			1.00	(0.57–1.74)
2002	0.69	(0.40–1.19)	0.71	(0.40–1.19)	0.73	(0.42–1.27)	0.73	(0.42–1.26)			0.64	(0.35–1.18)
2003	0.77	(0.42–1.39)	0.81	(0.42–1.39)	0.84	(0.46–1.52)	0.82	(0.45–1.49)			0.94	(0.50–1.79)
2004	0.83	(0.49–1.41)	0.88	(0.49–1.41)	0.93	(0.55–1.58)	1	ref			1.05	(0.60–1.84)
2005	0.60	(0.34–1.06)	0.63	(0.34–1.06)	0.66	(0.37–1.17)			0.71	(0.41–1.23)	0.68	(0.37–1.24)
2006	0.69	(0.40–1.20)	0.73	(0.40–1.20)	0.76	(0.44–1.32)			0.81	(0.49–1.36)	0.82	(0.46–1.46)
2007	0.74	(0.43–1.28)	0.80	(0.43–1.28)	0.84	(0.49–1.45)			0.91	(0.55–1.53)	0.92	(0.52–1.62)
2008	0.38	(0.20–0.72)	0.40	(0.20–0.72)	0.43	(0.23–0.82)			0.48	(0.26–0.89)	0.48	(0.25–0.93)
2009	0.63	(0.36–1.09)	0.68	(0.36–1.09)	0.73	(0.42–1.27)			0.81	(0.48–1.37)	0.66	(0.36–1.19)
2010	0.48	(0.27–0.86)	0.53	(0.27–0.86)	0.57	(0.32–1.01)			0.63	(0.36–1.10)	0.61	(0.34–1.12)
Previous admissions:												
Yes					4.52	(3.02–6.78)	4.27	(2.66–6.88)	4.94	(2.47–9.89)	4.16	(2.63–6.57)
No					1	ref	1	ref	1	ref	1	ref
Pregancy BMI:												
Underweight											0.44	(0.14–1.34)
Normal											1	ref
Overweight											0.95	(0.73–1.25)

For peer review only

Obese						1.04	(0.71–1.53)
Observations (N):	37,621	37,621	37,621	15,466	22,155	33,236	
Clusters (Maternal ID):	25,344	25,344	25,344	12,377	16,910	23,471	

*Adjusted for year of birth. Analysis included 37,621 children.

†Adjusted for year of birth small for gestational age, large for gestational age, congenital malformation, sex, maternal age, parity, maternal smoking, maternal education level and maternal country of birth. Analysis included 37,621 children.

‡Adjusted for year of birth small for gestational age, large for gestational age, congenital malformation, sex, maternal age, parity, maternal smoking, maternal education level, maternal country of birth and previous admissions. Analysis included 37,621 children.

§Included children born 1998 to 2003. Adjusted for year of birth small for gestational age, large for gestational age, congenital malformation, sex, maternal age, parity, maternal smoking, maternal education level, maternal country of birth and previous admissions. Analysis excluded children born 2004 to 2010, leaving 15,466 children.

¶Included children born 2004 to 2010. Adjusted for year of birth small for gestational age, large for gestational age, congenital malformation, sex, maternal age, parity, maternal smoking, maternal education level, maternal country of birth and previous admissions. Analysis excluded children born 1998 to 2003, leaving 22,155 children.

#Adjusted for year of birth small for gestational age, large for gestational age, congenital malformation, sex, maternal age, parity, maternal smoking, maternal education level, maternal country of birth, previous admissions and pregnancy BMI. Analysis excluded children with missing data on pregnancy BMI, leaving 33,236 children.

Incidence rate ratios were estimated using negative binomial regression models. Correlation between siblings was accounted for using generalized estimating equations with robust standard errors. CI, confidence intervals. BF, breastfeeding. EBF, exclusive breastfeeding. MO, months. SGA, small for gestation age. LGA, large for gestation age. BMI, body mass index.

For peer review only

Table K: Regression results for lower respiratory infections, later childhood (2–4 years)

	Crude[*]	(95% CI)	Adjusted[†]	(95% CI)	Adjusted 2[‡]	(95% CI)	1998-2003[§]	(95% CI)	2004-2010[¶]	(95% CI)	BMI[#]	(95% CI)
Breastfeeding:												
No BF	3.70	(1.76–7.78)	3.50	(1.69–7.24)	3.10	(1.49–6.45)	5.43	(2.33-12.65)	1.28	(0.27–6.12)	3.33	(1.56–7.11)
EBF <4 MO with BF <6 MO	1.79	(1.12–2.86)	1.93	(1.18–3.15)	1.87	(1.15–3.06)	1.62	(0.76–3.44)	2.04	(1.06–3.91)	1.81	(1.07–3.08)
EBF <4 MO with BF ≥6 MO	1.18	(0.70–2.00)	1.15	(0.67–1.96)	1.13	(0.66–1.93)	1.54	(0.71–3.36)	0.96	(0.47–1.98)	1.15	(0.66–2.03)
EBF 4–5 MO with BF <6 MO	0.46	(0.16–1.29)	0.51	(0.18–1.42)	0.50	(0.18–1.40)	0.86	(0.25–2.91)	0.24	(0.03–1.78)	0.54	(0.19–1.53)
EBF 4–5 MO with BF ≥6 MO	0.85	(0.56–1.28)	0.87	(0.58–1.31)	0.88	(0.58–1.32)	0.90	(0.48–1.67)	0.88	(0.51–1.51)	0.86	(0.56–1.32)
EBF ≥6 MO	1	ref	1	ref	1	ref	1	ref	1	ref	1	ref
SGA												
Yes			3.89	(1.86–8.15)	3.86	(1.84–8.10)	1.93	(0.57–6.50)	5.43	(2.29-12.85)	4.17	(1.99–8.74)
No			1	ref	1	ref	1	ref	1	ref	1	ref
LGA												
Yes			0.99	(0.53–1.85)	0.99	(0.53–1.88)	0.87	(0.32–2.40)	1.04	(0.47–2.31)	1.01	(0.51–2.01)
No			1	ref	1	ref	1	ref	1	ref	1	ref
Congenital malformation:												
Yes			5.40	(3.31–8.81)	4.91	(2.96–8.12)	4.47	(2.07–9.66)	4.75	(2.45–9.21)	4.43	(2.62–7.49)
No			1	ref	1	ref	1	ref	1	ref	1	ref
Sex												
Male			1	ref	1	ref	1	ref	1	ref	1	ref
Female			1.00	(0.73–1.36)	1.03	(0.75–1.42)	1.25	(0.78–2.00)	0.87	(0.58–1.31)	1.04	(0.75–1.46)
Maternal age (years):												
≤19			1.10	(0.23–5.16)	1.12	(0.24–5.29)	1.29	(0.27-23.97)	0.82	(0.09–7.22)	1.29	(0.27–6.24)
20-24			1	ref	1	ref	1	ref	1	ref	1	ref
25-29			1.57	(0.87–2.83)	1.53	(0.85–2.76)	2.68	(0.91–7.84)	1.22	(0.60–2.50)	1.66	(0.89–3.09)
30-34			1.38	(0.77–2.46)	1.34	(0.75–2.39)	4.01	(1.37-11.79)	0.69	(0.34–1.36)	1.45	(0.79–2.66)
≥35			1.97	(1.03–3.76)	1.94	(1.02–3.70)	6.07	(1.86-19.81)	0.98	(0.46–2.10)	2.01	(1.02–3.95)
Parity:												
1			1	ref	1	ref	1	ref	1	ref	1	ref
2			1.03	(0.72–1.47)	1.00	(0.69–1.43)	0.76	(0.44–1.30)	1.33	(0.85–2.09)	0.96	(0.66–1.40)
3			1.01	(0.58–1.76)	0.97	(0.55–1.71)	0.55	(0.22–1.39)	1.59	(0.79–3.18)	0.95	(0.51–1.76)
≥4			0.83	(0.42–1.66)	0.79	(0.39–1.60)	0.44	(0.18–1.10)	1.32	(0.50–3.48)	0.79	(0.38–1.65)
Maternal smoking:												

For peer review only

No smoking			1	ref	1	ref	1	ref	1	ref	1	ref
Smoking			0.53	(0.28–1.00)	0.51	(0.27–0.96)	0.52	(0.22–1.22)	0.52	(0.21–1.31)	0.45	(0.23–0.89)
Maternal education (years):												
≤9			0.92	(0.48–1.76)	0.91	(0.47–1.75)	0.88	(0.37–2.11)	0.82	(0.33–2.07)	1.01	(0.51–2.01)
10-12			0.95	(0.65–1.39)	0.95	(0.65–1.39)	1.23	(0.68–2.22)	0.75	(0.46–1.23)	1.00	(0.66–1.50)
13-14			0.99	(0.62–1.59)	0.99	(0.62–1.59)	0.90	(0.45–1.82)	1.07	(0.57–2.03)	0.98	(0.60–1.61)
≥15			1	ref	1	ref	1	ref	1	ref	1	ref
Maternal country of birth:												
Sweden			1	ref	1	ref	1	ref	1	ref	1	ref
Other Nordic countries			1.58	(0.74–3.36)	1.55	(0.73–3.31)	1.42	(0.45–4.51)	1.69	(0.62–4.60)	1.43	(0.63–3.25)
Europe and North America			0.22	(0.05–0.87)	0.22	(0.06–0.90)	0.33	(0.05–2.45)	0.17	(0.02–1.23)	0.24	(0.06–0.98)
Sweden			1.40	(0.79–2.47)	1.41	(0.80–2.51)	1.40	(0.62–3.16)	1.30	(0.58–2.91)	1.57	(0.88–2.80)
Africa			2.05	(0.69–6.06)	2.02	(0.69–5.95)	2.34	(0.79–6.92)	1.77	(0.34–9.14)	1.87	(0.56–6.18)
Other			1.63	(0.37–7.15)	1.64	(0.37–7.30)	3.09	(0.51-18.82)	0.69	(0.10–4.95)	0.50	(0.07–3.61)
Year of birth:												
1998	1	ref	1	ref	1	ref			1	ref	1	ref
1999	0.93	(0.37–2.38)	0.99	(0.37–2.38)	0.99	(0.39–2.53)	0.99	(0.39–2.51)			1.06	(0.40–2.80)
2000	1.26	(0.50–3.13)	1.34	(0.50–3.13)	1.30	(0.53–3.22)	1.21	(0.50–2.91)			1.36	(0.53–3.50)
2001	1.20	(0.46–3.12)	1.27	(0.46–3.12)	1.25	(0.48–3.20)	1.14	(0.46–2.83)			1.06	(0.38–3.02)
2002	1.22	(0.51–2.93)	1.24	(0.51–2.93)	1.19	(0.50–2.86)	1.08	(0.45–2.59)			1.21	(0.48–3.03)
2003	1.29	(0.56–2.96)	1.30	(0.56–2.96)	1.24	(0.54–2.84)	1.13	(0.50–2.58)			1.23	(0.51–2.98)
2004	1.19	(0.47–3.01)	1.25	(0.47–3.01)	1.21	(0.48–3.05)	1	ref			1.31	(0.49–3.49)
2005	1.14	(0.48–2.70)	1.15	(0.48–2.70)	1.06	(0.45–2.49)			0.89	(0.42–1.87)	0.95	(0.39–2.32)
2006	0.80	(0.28–2.27)	0.83	(0.28–2.27)	0.81	(0.28–2.33)			0.67	(0.26–1.74)	0.77	(0.25–2.42)
2007	1.14	(0.49–2.62)	1.14	(0.49–2.62)	1.09	(0.47–2.53)			0.89	(0.43–1.87)	1.19	(0.50–2.83)
2008	1.00	(0.40–2.53)	1.04	(0.40–2.53)	1.02	(0.41–2.54)			0.85	(0.37–1.97)	0.99	(0.38–2.61)
2009	1.46	(0.64–3.32)	1.55	(0.64–3.32)	1.47	(0.65–3.36)			1.23	(0.59–2.59)	1.55	(0.66–3.63)
2010	0.74	(0.31–1.77)	0.83	(0.31–1.77)	0.77	(0.32–1.87)			0.64	(0.29–1.44)	0.79	(0.32–1.97)
Previous admissions:												
Yes					6.29	(3.44-11.50)	9.65	(3.41-27.27)	4.23	(2.15–8.32)	6.58	(3.50-12.38)
No					1	ref	1	ref	1	ref	1	ref
Pregancy BMI:												
Underweight											0.72	(0.21–2.48)
Normal											1	ref
Overweight											0.68	(0.42–1.11)

For peer review only

Obese						1.06	(0.68–1.65)
Observations (N):	37,621	37,621	37,621	15,466	22,155	33,236	
Clusters (Maternal ID):	25,344	25,344	25,344	12,377	16,910	23,471	

*Adjusted for year of birth. Analysis included 37,621 children.

†Adjusted for year of birth small for gestational age, large for gestational age, congenital malformation, sex, maternal age, parity, maternal smoking, maternal education level and maternal country of birth. Analysis included 37,621 children.

‡Adjusted for year of birth small for gestational age, large for gestational age, congenital malformation, sex, maternal age, parity, maternal smoking, maternal education level, maternal country of birth and previous admissions. Analysis included 37,621 children.

§Included children born 1998 to 2003. Adjusted for year of birth small for gestational age, large for gestational age, congenital malformation, sex, maternal age, parity, maternal smoking, maternal education level, maternal country of birth and previous admissions. Analysis excluded children born 2004 to 2010, leaving 15,466 children.

¶Included children born 2004 to 2010. Adjusted for year of birth small for gestational age, large for gestational age, congenital malformation, sex, maternal age, parity, maternal smoking, maternal education level, maternal country of birth and previous admissions. Analysis excluded children born 1998 to 2003, leaving 22,155 children.

#Adjusted for year of birth small for gestational age, large for gestational age, congenital malformation, sex, maternal age, parity, maternal smoking, maternal education level, maternal country of birth, previous admissions and pregnancy BMI. Analysis excluded children with missing data on pregnancy BMI, leaving 33,236 children.

Incidence rate ratios were estimated using negative binomial regression models. Correlation between siblings was accounted for using generalized estimating equations with robust standard errors. CI, confidence intervals. BF, breastfeeding. EBF, exclusive breastfeeding. MO, months. SGA, small for gestation age. LGA, large for gestation age. BMI, body mass index.

For peer review only

STROBE Statement—Checklist of items that should be included in reports of ***cohort studies***

	Item No	Recommendation	Page No
**Title and abstract**	1	(*a*) Indicate the study's design with a commonly used term in the title or the abstract	1-2
		(*b*) Provide in the abstract an informative and balanced summary of what was done and what was found	2
**Introduction**			
Background/rationale	2	Explain the scientific background and rationale for the investigation being reported	4
Objectives	3	State specific objectives, including any prespecified hypotheses	4
**Methods**			
Study design	4	Present key elements of study design early in the paper	5
Setting	5	Describe the setting, locations, and relevant dates, including periods of recruitment, exposure, follow-up, and data collection	5-7
Participants	6	(*a*) Give the eligibility criteria, and the sources and methods of selection of participants. Describe methods of follow-up	5-7
		(*b*) For matched studies, give matching criteria and number of exposed and unexposed	
Variables	7	Clearly define all outcomes, exposures, predictors, potential confounders, and effect modifiers. Give diagnostic criteria, if applicable	5-7
Data sources/ measurement	8*	For each variable of interest, give sources of data and details of methods of assessment (measurement). Describe comparability of assessment methods if there is more than one group	5-7
Bias	9	Describe any efforts to address potential sources of bias	7
Study size	10	Explain how the study size was arrived at	-
Quantitative variables	11	Explain how quantitative variables were handled in the analyses. If applicable, describe which groupings were chosen and why	7
Statistical methods	12	(*a*) Describe all statistical methods, including those used to control for confounding	7
		(*b*) Describe any methods used to examine subgroups and interactions	
		(*c*) Explain how missing data were addressed	
		(*d*) If applicable, explain how loss to follow-up was addressed	
		(*e*) Describe any sensitivity analyses	
**Results**			
Participants	13*	(a) Report numbers of individuals at each stage of study—eg numbers potentially eligible, examined for eligibility, confirmed eligible, included in the study, completing follow-up, and analysed	8
		(b) Give reasons for non-participation at each stage	
		(c) Consider use of a flow diagram	
Descriptive data	14*	(a) Give characteristics of study participants (eg demographic, clinical, social) and information on exposures and potential confounders	8 + Tabell 1
		(b) Indicate number of participants with missing data for each variable of interest	Tabell 1
		(c) Summarise follow-up time (eg, average and total amount)	8
Outcome data	15*	Report numbers of outcome events or summary measures over time	8

For peer review only

Main results	16	(*a*) Give unadjusted estimates and, if applicable, confounder-adjusted estimates and their precision (eg, 95% confidence interval). Make clear which confounders were adjusted for and why they were included<br>(*b*) Report category boundaries when continuous variables were categorized<br>(*c*) If relevant, consider translating estimates of relative risk into absolute risk for a meaningful time period	9<br><br>Appendix B and C
Other analyses	17	Report other analyses done—eg analyses of subgroups and interactions, and sensitivity analyses	Appendix B and C
**Discussion**			
Key results	18	Summarise key results with reference to study objectives	10
Limitations	19	Discuss limitations of the study, taking into account sources of potential bias or imprecision. Discuss both direction and magnitude of any potential bias	11-12
Interpretation	20	Give a cautious overall interpretation of results considering objectives, limitations, multiplicity of analyses, results from similar studies, and other relevant evidence	11-12
Generalisability	21	Discuss the generalisability (external validity) of the study results	12
**Other information**			
Funding	22	Give the source of funding and the role of the funders for the present study and, if applicable, for the original study on which the present article is based	13

*Give information separately for exposed and unexposed groups.

Note: An Explanation and Elaboration article discusses each checklist item and gives methodological background and published examples of transparent reporting. The STROBE checklist is best used in conjunction with this article (freely available on the Web sites of PLoS Medicine at http://www.plosmedicine.org/, Annals of Internal Medicine at http://www.annals.org/, and Epidemiology at http://www.epidem.com/). Information on the STROBE Initiative is available at http://www.strobe-statement.org.

For peer review only
